# Two *Fusarium* copper radical oxidases with high activity on aryl alcohols

**DOI:** 10.1186/s13068-021-01984-0

**Published:** 2021-06-16

**Authors:** Maria Cleveland, Mickael Lafond, Fan Roderick Xia, Ryan Chung, Paul Mulyk, Jason E. Hein, Harry Brumer

**Affiliations:** 1 Michael Smith Laboratories, University of British Columbia, 2185 East Mall, Vancouver, BC V6T 1Z4 Canada; 2 Department of Chemistry, University of British Columbia, 2036 Main Mall, Vancouver, BC V6T 1Z1 Canada; 3 BioProducts Institute, University of British Columbia, 2385 East Mall, Vancouver, BC V6T 1Z4 Canada; 4 Aix Marseille Univ, CNRS, Centrale Marseille, iSm2, Marseille, France; 5 Department of Biochemistry and Molecular Biology, University of British Columbia, 2350 Health Sciences Mall, Vancouver, BC V6T 1Z3 Canada; 6 Department of Botany, University of British Columbia, 3200 University Boulevard, Vancouver, BC V6T 1Z4 Canada

**Keywords:** Copper radical oxidase, Aryl alcohol oxidase, Galactose oxidase, Biocatalysis, Metalloenzyme

## Abstract

**Background:**

Biomass valorization has been suggested as a sustainable alternative to petroleum-based energy and commodities. In this context, the copper radical oxidases (CROs) from Auxiliary Activity Family 5/Subfamily 2 (AA5_2) are attractive biocatalysts for the selective oxidation of primary alcohols to aldehydes. Originally defined by the archetypal galactose 6-oxidase from *Fusarium graminearum*, fungal AA5_2 members have recently been shown to comprise a wide range of specificities for aromatic, aliphatic and furan-based alcohols. This suggests a broader substrate scope of native CROs for applications. However, only 10% of the annotated AA5_2 members have been characterized to date.

**Results:**

Here, we define two homologues from the filamentous fungi *Fusarium graminearum* and *F. oxysporum* as predominant aryl alcohol oxidases (AAOs) through recombinant production in *Pichia pastoris*, detailed kinetic characterization, and enzyme product analysis. Despite possessing generally similar active-site architectures to the archetypal *Fgr*GalOx, *Fgr*AAO and *Fox*AAO have weak activity on carbohydrates, but instead efficiently oxidize specific aryl alcohols. Notably, both *Fgr*AAO and *Fox*AAO oxidize hydroxymethyl furfural (HMF) directly to 5-formyl-2-furoic acid (FFCA), and desymmetrize the bioproduct glycerol to the uncommon L-isomer of glyceraldehyde.

**Conclusions:**

This work expands understanding of the catalytic diversity of CRO from AA5_2 to include unique representatives from *Fusarium* species that depart from the well-known galactose 6-oxidase activity of this family. Detailed enzymological analysis highlights the potential biotechnological applications of these orthologs in the production of renewable plastic polymer precursors and other chemicals.

**Supplementary Information:**

The online version contains supplementary material available at 10.1186/s13068-021-01984-0.

## Background

Our dependency on non-renewable resources and the associated environmental consequences pose as an existential crisis to human society [1, 2]. One avenue to reduce consumption of fossil petroleum is to utilize biomass as a renewable, alternative carbon source. In this regard, the development of integrated biorefineries that produce multiple products, including fuels, chemicals, and materials (collectively “bio-products”) would advance the goal to lower net carbon emissions [3, 4]. In particular, harnessing the inherent chemical functionality of biomass constituents to produce high-value bio-products through selective derivatization is currently envisioned as a better value proposition than complete saccharification and fermentation to commodity fuels [5].

In nature, terrestrial biomass is efficiently transformed and degraded by fungi via a plethora of specialized enzymes that could be harnessed in biocatalytic processes for the industrial production of bio-products [6–8]. In particular, fungi have a rich history as sources of hydrolytic enzymes for biomass saccharification [9–12]. Fungi also produce a diversity of redox enzymes, which act on a wide range of substrates [13]. Notably, there is increasing interest in the use of redox enzymes to replace chemical oxidants, many of which generate hazardous waste co-products. Indeed, bio-oxidation reactions account for one-sixth of the biocatalysis performed industrially, including hydroxylation, Baeyer–Villiger catalysis, alcohol and amine oxidation, and other transformations [7, 14]. Presently, the majority of these transformations are performed by FAD- and NAD(P)-dependent enzymes, which require complex organic cofactors, and in the latter case, cofactor recycling strategies [6].

In contrast, copper radical oxidases (CROs) [15], have received considerably less attention regarding their potential as industrial biocatalysts [7, 16]. CROs comprise Auxiliary Activity Family 5 in the Carbohydrate-Active Enzymes (CAZy) classification, and thus share similar tertiary structures and active sites containing a mononuclear copper center [15, 17–19]. AA5 has been further divided into two subfamilies based on molecular phylogeny. Subfamily 1 (AA5_1) comprises (methyl)glyoxal oxidases (E.C. 1.2.3.15) that oxidize select aldehydes, likely via the hydrated *gem*-diol, to the corresponding carboxylic acid with concomitant reduction of O_2_ to H_2_O_2_ [20]. Subfamily 2 (AA5_2) contains the founding galactose oxidases (GalOx, E.C.1.1.3.9) [21–25] as well as the more recently discovered general alcohol oxidases (AlcOx, E.C. 1.1.3.13) [18, 26] and aryl alcohol oxidases (AAO, E.C. 1.1.3.7) [19], all of which convert the primary alcohol of the substrate to the corresponding aldehyde in an analogous two electron oxidation. The specific biological roles of CROs are currently unknown, although they have been speculated to play a role in oxidative lignocellulose degradation and have been linked to fungal pathogenesis [15, 27].

The archetypal galactose oxidase from the phytopathogen *Fusarium graminearum* (*Fgr*GalOx) has been used in many biotechnological applications such as glycoprotein labeling [28–31], construction of lactose biosensors [32], chemo-enzymatic modification of galactose and galactosides [33], and complex polysaccharide modification for the development of functional materials [34–38]. As the only characterized member of AA5 for many years, *Fgr*GalOx has been the subject of many protein engineering and directed evolution experiments to broaden substrate scope to include activity on glucose [39, 40], fructose [41], mannose [42], N-acetylglucosamine [42], secondary alcohols [43], amino alcohols [44] and benzyl alcohols [45]. Other studies have been performed to increase the catalytic efficiency of *Fgr*GalOx [46–49]. Especially notable, a highly evolved variant of *Fgr*GalOx was central to the recent industrial development of a biocatalytic synthesis of the drug islatravir [50]. Thus, there is significant scope to expand the application space of CROs to meet the need for new oxidation catalysts dependent on substrate range [7, 16, 51, 52].

The diversity of CROs from AA5 remains largely unexplored, but features a breadth of sequences from fungal sources that can be mined for potentially new industrial applications. Of the hundreds of publicly available putative AA5 sequences currently in the CAZy database [13], only ten AA5_2 members from this subfamily have been biochemically characterized, with the majority (five) characterized as galactose or galactoside oxidases (four from *Fusarium* species) [53–56]. In this context, we have recently revealed novel non-carbohydrate-active CROs in AA5_2 from the phytopathogenic fungus *Colletotrichum graminicola*: *Cgr*AlcOx displays high activity on primary aliphatic, benzyl, and other unsaturated alcohols [18], while *Cgr*AAO is most active on a range of benzyl alcohols and is also active on hydroxymethylfurfural (HMF, EC 1.1.3.47) [19].

Intrigued by this broader catalytic potential of AA5_2, we have used molecular phylogeny to guide the selection of two distinct homologs from *F. graminearum* and *F. oxysporum* for recombinant expression and biochemical characterization. Notably, both enzymes had low activity on carbohydrates, but displayed high activity on aryl alcohols, such as veratryl alcohol, and have considerable activity on HMF and oxidized congeners. Detailed enzyme kinetic and product analysis, together with molecular modeling of substrate recognition, allowed us to rationalize this specificity profile with regard to the composition of the active site.

## Results and discussion

### Bioinformatics

**Fig. 1:**
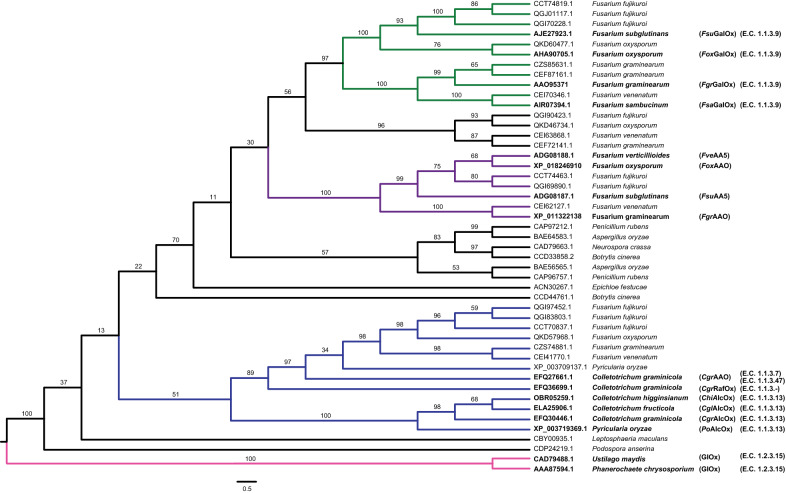
Molecular phylogeny of 45 AA5_2 members. The sequences of two AA5_1 (methyl)glyoxal oxidase catalytic modules [60] were included as an outgroup to root the tree (highlighted in pink). Clades containing characterized *Colletotrichum* enzymes are highlighted in blue, *Fgr*AAO and *Fox*AAO in purple and *Fgr*GalOx in green. GenBank Accessions are given for all sequences from the public CAZy database (February 2021) with the genus and species names of source organisms with E.C. numbers also listed. Data from previous studies comprise AAO95371 (*Fgr*GalOx) [17, 57, 58], AHA90705.1 (*Fox*GalOx) [54], AIR07394.1 (*Fsa*GalOx) [53], AJE27923 (*Fsu*GalOx) [56], ADG08188.1 and ADG08187.1 (*Fve*AA5 and *Fsu*AA5) [59], XP_011322138 and XP_018246910 (*Fgr*AAO and *Fox*AAO, this study), EFQ27661.1 (*Cgr*AAO) [19], EFQ30446 and ELA25906.1 (*Cgr*AlcOx and *Cgl*AlcOx) [18], EFQ36699.1 (*Cgr*RafOx) [55], OBR05259.1 (*Chi*AlcOx) and XP_003719369.1 (*Po*AlcOx) [26]

Building upon our previous discovery of unique substrate specificities among *Colletotrichum* AA5_2 homologs [18, 19], we were keen to explore alternative homologs of AA5_2 members from *Fusarium* species, from which a number of galactose oxidases have been described [53, 54, 56], including the archetypal *Fgr*GalOx [17, 25, 57, 58]. A molecular phylogeny was generated to guide target selection, using 45 AA5_2 catalytic modules selected from the CAZy database [13], including previously characterized homologs from *Fusarium* and *Colletotrichum* (Fig. 1). We observed that a homolog from *F. graminearum* (GenBank XP_011322138) grouped into a strongly supported clade that was notably distant from that containing *Fgr*GalOx. This clade is also distinct from the early diverging clade comprising the *Colletotrichum* AlcOxs and AAO. To our knowledge, there are no biochemically characterized members of this clade [59], thus warranting further study. We also selected the homolog from *F. oxysporum* (GenBank XP_018246910) for characterization, as a second example from this clade. Hereafter, these homologs will be referred to as *Fgr*AAO and *Fox*AAO, respectively, based on subsequent substrate specificity analysis (*vide infra*).

Concordant with this phylogeny, a protein sequence alignment of *Fgr*AAO and *Fox*AAO revealed that they are highly similar (91% pairwise identity, Additional file 1: Table S1). *Fgr*AAO and *Fox*AAO have *ca*. 60% pairwise identity versus the AA5 archetype *Fgr*GalOx [17, 61] and with characterized GalOx from *F. oxysporum, F. sambucinum* and *F. subglutinans* [53, 54, 56] (Fig. 1 and Additional file 1: Table S1). On the other hand, *Fgr*AAO and *Fox*AAO have low sequence similarity with the characterized *Colletotrichum* AA5 members, *Cgr*AlcOx and *Cgr*AAO (*ca.* 50% identity, Additional file 1: Table S1). Particularly notable, *Fgr*AAO and *Fox*AAO share high sequence identity (80%) with orthologs from *F. verticillioides* and *F. subglutinans*, which have been cloned but not characterized in recombinant form (Fig. 1 and Additional file 1: Table S1) [59].

**Fig. 2:**
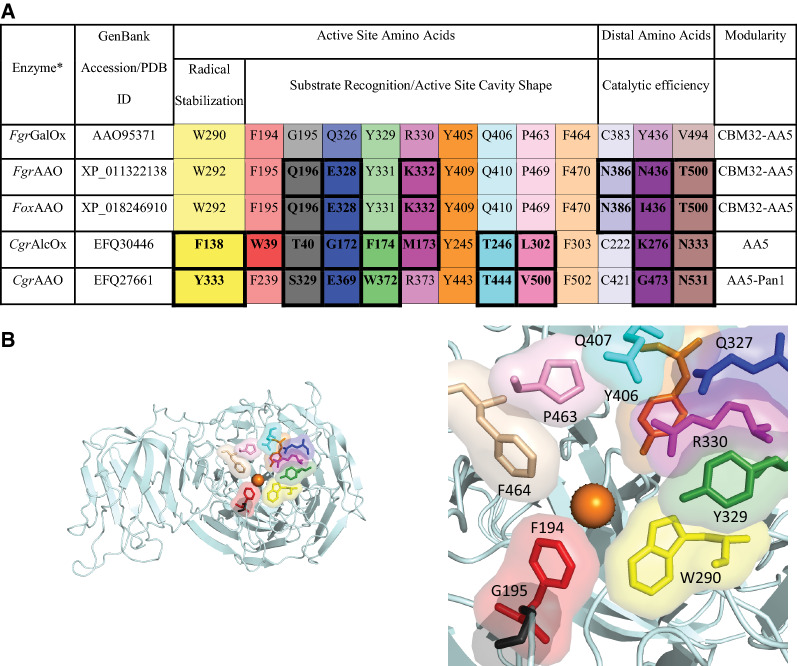
Active site amino acids in AA5_2 members. **A** Key amino acid residues in the two candidate enzymes compared to other characterized AA5_2 copper radical oxidases. Non-conserved amino acids in relation to *Fgr*GalOx are bolded. Fungal species: *Fgr*, *Fusarium graminearum; Fox*, *Fusarium oxysporum; Cgr, Colletotrichum graminicola.* **B** Active site of *Fgr*GalOx. Left panel—full protein, right panel—zoom of active site. Colors are coordinated with amino acid residues in Panel A

It is worth underscoring that the phylogeny shown in Fig. 1 was calculated based on AA5 catalytic modules only, and that *Fgr*AAO and *Fox*AAO have the same modular organization as *Fgr*GalOx, viz*.* an N-terminal carbohydrate-binding module (CBM32) [62] in tandem with the AA5_2 module. In contrast, *Cgr*AlcOx and *Cgr*AAO lack an N-terminal carbohydrate-binding module (Fig. 2). Detailed sequence analysis indicated that key active-site and secondary shell radical-stabilizing residues are largely conserved between *Fgr*GalOx, *Fgr*AAO and *Fox*AAO (Fig. 2). A notable exception is the Arg-to-Lys substitution in *Fgr*AAO and *Fox*AAO corresponding to K330 in *Fgr*GalOx. Site-directed mutation of this residue in *Fgr*GalOx has been shown to increase relative activities on fructose (in wild-type *Fgr*GalOx) [41] and mannose (in M_1_ variant) [42], thus highlighting a role in substrate specificity. Mutation of the active-site residues G195 and Q326 in *Fgr*GalOx has been shown to improve protein stability [33] and alter substrate specificity [42], respectively, while the distal residues C383, Y436, and V494 have been implicated in affecting catalytic efficiency [46–49]. *Fgr*AAO and *Fox*AAO are distinguished from the wild-type *Fgr*GalOx in these positions and differ from each other at position Y436 (Fig. 2).

### Protein production and substrate screening

cDNA corresponding to the mature, full-length *Fgr*AAO and *Fox*AAO proteins comprising the N-terminal CBM32 and C-terminal AA5_2 module (lacking the native signal peptide) were cloned into pPICZα-A vector in-frame with a *Saccharomyces cerevisiae* α-factor secretion signal peptide and a C-terminal hexa-histidine tag. Both proteins were successfully produced in *Pichia pastoris* X33 and purified by a two-step IMAC-SEC protocol, with typical yields of 1 mg of protein per 400 mL of expression medium. Notably, *Fox*AAO eluted as a single pure peak from the size exclusion column, whereas *Fgr*AAO eluted as two peaks, the second of which contained pure enzyme, as determined by SDS-PAGE (Additional file 1: Figure S2). Both protein production levels are significantly lower (200-fold) than recombinant wild-type *Fgr*GalOx [63] produced in *P. pastoris. Fgr*AAO and *Fox*AAO (2.5 mg L^−1^) produce similarly to *Fox*GalOx (10.6 mg L^−1^) [54] and had production values lower than wild-type *Fgr*GalOx and *Fgr*GalOx M_1_ constructs with histidine tags (120 mg L^−1^ and 110 mg L^−1^, respectively) [64], *Cgr*AlcOx (30–40 mg L^−1^) [18] and *Cgr*AAO (42.5 mg L^−1^) [19].

In light of the sequence similarity of *Fgr*AAO and *Fox*AAO to *Fgr*GalOx (Additional file 1: Table S1), galactose was selected as an initial substrate for optimum pH and temperature determination. Both recombinant enzymes were active in the pH range from 5 to 10, with bell-shaped pH-rate profiles and optimum pH values of *ca.* 7.5 in sodium phosphate buffer (Additional file 1: Figure S3). This pH optimum is similar to other characterized AA5_2 members [18, 19, 53, 54, 65]. *Fgr*AAO and *Fox*AAO were rapidly inactivated above 65 °C (Additional file 1: Figure S4). Additional stability assays indicated that both enzymes were stable at 30 °C over *ca.* 6 h, but lost a significant amount of activity with extended incubation at this and higher temperatures (Additional file 1: Figure S5). Overall, *Fgr*AAO and *Fox*AAO appear to have lower thermostability than other AA5_2 members [18, 54].

**Fig. 3:**
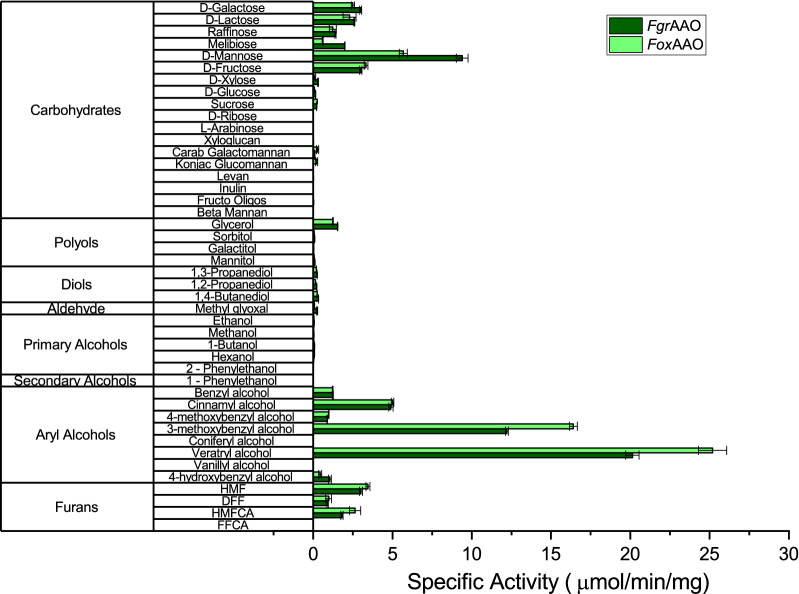
Initial activity screens of *Fgr*AAO and *Fox*AAO. Measurements were performed in triplicate at 25 °C in 50 mM sodium phosphate buffer, pH 7.5, using the HRP/ABTS assay. Activities were monitored using 300 mM for carbohydrates, polyols, diols and primary alcohols, 2.5 mg mL^−1^ for polysaccharides, 30 mM for benzyl alcohol and galactitol, 5 mM for methyl glyoxal, aryl alcohols and furans and 10 mM for secondary alcohols. Reactions were started with the addition of 6 pmol–1 nmole of purified enzyme (Additional file 1: Table S2)

*Fgr*AAO and *Fox*AAO were then screened against a broad panel of primary alcohols, including carbohydrates, alkanols, and aryl alcohols of biological and synthetic relevance (Fig. 3 and Additional file 1: Table S2). Concordant with our initial activity assays, galactose and the galactosylated di- and trisaccharides melibiose, lactose, and raffinose were competent substrates of *Fgr*AAO and *Fox*AAO, with specific activities up to 3 μmol min^−1^ mg^−1^ (Fig. 3 and Additional file 1: Table S2). In contrast, activity on xyloglucan or galactomannan, which bear terminal galactosyl (t-Gal) branch residues, was extremely limited. Strikingly, the highest monosaccharide activity was observed with mannose, while activity on the ketose, fructose, was comparable to that on galactose (Fig. 3 and Additional file 1: Table S2). Yet, very limited activity was detected on sucrose (α-d-glucopyranosyl-(1 → 2)-β-d-fructofuranoside) and β-(2 → 1) fructo-oligosaccharides.

Primary alcohols, including a range of alkanols, diols, and alditols were generally not substrates for *Fgr*AAO and *Fox*AAO, although glycerol was a competent substrate with a specific activity of 1.5 μmol min^−1^ mg^−1^ (one-half that of galactose). Although benzyl alcohol and cinnamyl alcohol were substrates for both enzymes, with cinnamyl alcohol preferred, the highest activities for *Fgr*AAO and *Fox*AAO were observed with the substituted aryl alcohols 3-methoxybenzyl alcohol (*m*-anisyl alcohol) and veratryl alcohol (3,4-dimethoxybenzyl alcohol). At the same time, 4-hydroxybenzyl alcohols (i.e., 4-hydroxybenzyl, vanillyl, and coniferyl alcohols) were not tolerated by either enzyme, and low activity on 4-methoxybenzyl alcohol (*p*-anisyl alcohol) was also observed. *Fgr*AAO and *Fox*AAO also exhibited activity on the furan substrates hydroxymethylfurfural (HMF), 5-(hydroxymethyl) furan-2-carboxylic acid (HMFCA) and 2,5-furandicarboxaldehyde (DFF) at levels similar to that of galactosyl substrates. This activity profile is notably distinct from that of the recently characterized *Cgr*AAO [19], as well as *Cgr*AlcOx [18]. In particular, *Cgr*AAO does not show a significant preference for the substitution pattern on the aromatic ring of benzyl alcohols [19]. The regioselectivity of *Fgr*AAO and *Fox*AAO is similar to the engineered *Fgr*GalOx variant M_3-5_, which has 1.4-fold higher activity on 3-methoxybenzyl alcohol than on 4-methoxybenzyl alcohol [45].

### Michaelis–Menten kinetics and product analysis

The results of our initial activity screen, in particular the predominant activity on 3-methoxybenzyl alcohol and veratryl alcohol, suggests that both enzymes may be best named as aryl alcohol oxidases (EC 1.1.3.7). However, this is not unequivocal as select carbohydrates and furans exhibited significant specific activity values at the substrate concentrations tested. To further define the substrate specificity of *Fgr*AAO and *Fox*AAO, Michaelis–Menten kinetic analysis was performed to determine *k*_cat_ and *K*_M_ values for the lead substrates (Additional file 1: Figure S6 and Table 1). In these cases, assays of *Fgr*AAO and *Fox*AAO were performed at 35 °C to balance optimal enzyme activity with short-term stability (*vide supra*).

**Table 1:** Substrate specificity of *Fgr*AAO and *Fox*AAO

Substrate	*Fgr*AAO			*Fox*AAO		
	*K*_*M*_ (mM)	*k*_cat_ (s^−1^)	*k*_cat_/*K*_*M*_ (M^−1^ s^−1^)	*K*_*M*_ (mM)	*k*_cat_ (s^−1^)	*k*_cat_/*K*_*M*_ (M^−1 ^s^−1^)
Carbohydrates						
Galactose	1700 ± 150	21 ± 1.0	12	1600 ± 150	23 ± 1.2	14
Galactose	450 ± 74	36 ± 1.2	79	620 ± 57	19 ± 0.35	31
Fructose	460 ± 91	9.7 ± 0.55	21	510 ± 82	16 ± 0.72	31
Polyols						
Glycerol	2200 ± 360	17 ± 1.2	7.7	2700 ± 403	24 ± 1.6	8.8
Aryl alcohols						
Benzyl alcohol	86 ± 12	37 ± 2.8	430	30 ± 19	31 ± 4.2	1000 (350 ± 20)^a^
Cinnamyl alcohol	n.d.^b^	n.d.^b^	3000	n.d.^b^	n.d.^b^	3200
4-Methoxybenzyl (*p*-anisyl) alcohol	59 ± 7.2	32 ± 1.8	540	51 ± 12	34 ± 2.9	290
3-Methoxybenzyl (*m*-anisyl) alcohol	6.5 ± 1.5	52 ± 1.5	8000	6.7 ± 0.59	34 ± 1.7	5100
3,4-Dimethoxybenzyl (veratryl) alcohol	3.7 ± 0.75	41 ± 4.0	11000	3.0 ± 0.24	25 ± 1.2	8300
Furans						
HMF	14 ± 2.6	29 ± 1.7	2100	17 ± 4.9	26 ± 2.6	1500
DFF	10 ± 2.9	3.6 ± 0.27	360	3.7 ± 1.0	1.4 ± 0.13	380
HMFCA	4.4 ± 0.90	3.8 ± 0.33	860	3.1 ± 0.31	5.2 ± 0.14	1700

Errors represent the standard deviation from the mean values

^a^*K*_M_, *k*_cat_, and *k*_cat_*/K*_M_ values calculated from fitting of the standard Michaelis–Menten equation to the data. Value in parentheses is the *k*_cat_*/K*_M_ value obtained from a linear fit to the data in the substrate range 1–50 mM. Fitting of the data with a modified Michaelis–Menten equation accounting for substrate inhibition was unsuccessful due to very high errors on individual parameters (see Additional file 1: Figure S6)

^b^Individual *K*_M_ and *k*_cat_ values not determinable due to substrate solubility limitations; *k*_cat_*/K*_M_ values obtained from slope of linear v_0_ versus [S] plots (Additional file 1: Figure S6)

This analysis revealed that the monosaccharides galactose, mannose, and fructose, and the triol glycerol, are in fact poor substrates for both *Fgr*AAO and *Fox*AAO. Although *k*_cat_ values for these substrates are comparable to those of benzyl alcohols and furans, *K*_M_ values are in the 0.5–2.0 M range. This indicates poor active-site affinity and selectivity for these polyhydroxylated compounds, with *k*_cat_/*K*_M_ values only in the range 10–80 M^−1^ s^−1^. Specifically, galactose was indeed a poor substrate for both *Fgr*AAO and *Fox*AAO, displaying ~ 1000-fold lower *k*_cat_/*K*_M_ values than the canonical *Fgr*GalOx [41], due to *ca.* 25-fold higher *K*_M_ values and *ca.* 50-fold lower *k*_cat_ values. In contrast, *k*_cat_/*K*_M_ values for competent benzyl alcohol substrates are in the range 10^3^–10^4^ M^−1^ s^−1^, while that of HMF is *ca*. 2000 M^−1^ s^−1^ for both enzymes. In these cases, this is largely due to *K*_M_ values in the millimolar range (Table 1).

### Aryl alcohol oxidation

*Benzyl alcohols* The much higher specificity constants of *Fgr*AAO and *Fox*AAO on benzyl and furan alcohols validates the designation of these enzymes as aryl alcohol oxidases (EC 1.1.3.7). Concordant with their grouping in the same phylogenetic clade (Fig. 1), both enzymes displayed similar catalytic parameters on those substrates for which detailed kinetics were performed, with the highest activities toward veratryl alcohol, 3-methoxybenzyl (*m*-anisyl) alcohol, and cinnamyl alcohol (Table 1). *k*_cat_/*K*_M_ values for these substrates were also similar to those observed for *Cgr*AAO [19] and *Fgr*GalOx [66], for which these are secondary substrates (Additional file 1: Table S4). Distinctly, the broad-spectrum aliphatic/aryl alcohol oxidase *Cgr*AlcOx [18] is *ca.* 2 orders of magnitude more efficient than *Fgr*AAO and *Fox*AAO on benzyl and cinnamyl alcohols, but does not oxidize other substrates of these enzymes, i.e., 4-methoxybenzyl alcohol (*p*-anisyl alcohol) and 4-hydroxybenzyl alcohol (Fig. 3 and Table 1). Together, specific activity and Michaelis–Menten kinetic data for *Fgr*AAO and *Fox*AAO indicate that these enzymes favor 3-methoxy substitutions on the benzyl ring, and strongly disfavor substrates with 4-hydroxy substitutions (Fig. 3 and Table 1).

In the absence of an enzyme–substrate complex structure, it is presently unclear how a balance of electronic (e.g., the Hammett substituent constants for 3-methoxybenzyl, 4-methoxybenzyl, and veratryl alcohols are + 0.12, −0.27, and −0.15, respectively [67]) and steric effects (e.g., specific recognition of the 3-methoxy group) dictate the observed specificities. Interestingly, the R330K mutant of *Fgr*GalOx showed a 63-fold decrease in activity on 3-methoxybenzyl alcohol compared to WT *Fgr*GalOx [41]. This result juxtaposes our current observations that *Fgr*AAO and *Fox*AAO natively possess an R330K mutation, yet exhibit a twofold increase in catalytic efficiency on 3-methoxybenzyl alcohol compared to WT *Fgr*GalOx. These differences might be explained by the additional Q326E substitution that may make the *Fgr*AAO and *Fox*AAO active sites more accessible to the methoxy-substituted substrate.

**Fig. 4:**
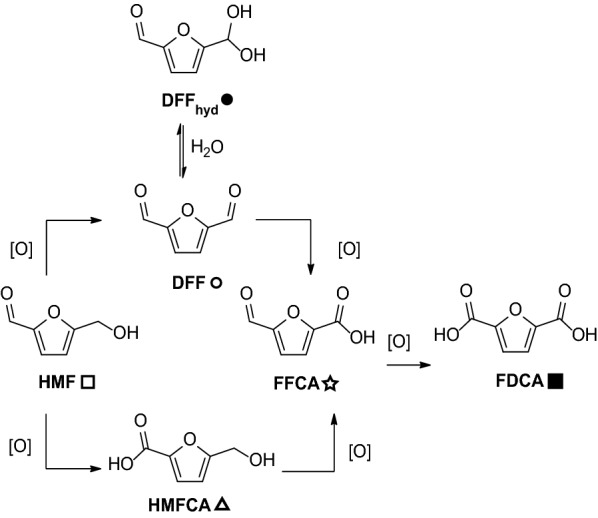
HMF oxidation. [O] represents a generic oxidant. The symbols next to each compound are used to denote the corresponding peaks in the NMR spectra comprising Additional file 1: Figures S7–S9

*Furans* The observation of demonstrable 5-(hydroxymethyl) furfural oxidation by *Fgr*AAO and *Fox*AAO is also consistent with the assignment of these enzymes as aryl alcohol oxidases (see EC 1.1.3.47 vs. EC 1.1.3.7). The conversion of HMF from biomass sources into the bis-functional polymer precursors DFF and FDCA is of significant contemporary interest [68, 69]. Select oxidases are capable of sequential oxidation of HMF to the various intermediates shown in Fig. 4 [14, 70–74]. Hence, we analyzed the reaction kinetics and products of *Fgr*AAO and *Fox*AAO in each of these steps.

*Fgr*AAO and *Fox*AAO display similar specificity constants on HMF to the eponymous bacterial HMF oxidase (a GMC superfamily oxidoreductase) [73], and exhibit higher specificity constants than other enzymes with HMF oxidase activity, including some glyoxal oxidases from AA5_1 (Additional file 1: Table S5) [70, 71, 74]. However, the recently reported *Cgr*AAO [19] has tenfold higher activity on HMF than both *Fgr*AAO and *Fox*AAO (Additional file 1: Table S5).

Product analysis by ^1^H-NMR spectroscopy following extended incubation with HMF revealed a product mixture of DFF and FFCA (Additional file 1: Figure S7), consistent with the initial formation of DFF and subsequent oxidation to FFCA, presumably via the aldehyde hydrate [20, 74]. Indeed, the conversion of DFF to FFCA was directly demonstrated by initial-rate kinetics and product analysis, although this oxidation step was considerably slower (Table 2 and Additional file 1: Figure S8). Furthermore, ^1^H NMR analysis following the incubation of *Fgr*AAO or *Fox*AAO with HMF at 1.5 h and 3 h did not indicate the formation of HMFCA, which argues against the lower pathway shown in Fig. 4. Neither *Fgr*AAO nor *Fox*AAO was able to oxidize FFCA to FDCA under the conditions tested, perhaps due to the low degree of hydration of FFCA compared with DFF [74]. Furfural was also not oxidized by either enzyme in overnight reactions. Interestingly, HMFCA was also a good substrate for *Fgr*AAO and *Fox*AAO, albeit with lower *k*_cat_ and *K*_M_ values than HMF, but with significant conversion to FFCA in extended incubations (Table 2 and Additional file 1: Figure S9). In total, the specificity profiles of *Fgr*AAO and *Fox*AAO are significantly different than those of other oxidases that are active on HMF and its derivatives [70–74], as summarized in Additional file 1: Table S5.

**Table 2:** Percent conversion of HMF, DFF and HMFCA by *Fgr*AAO and *Fox*AAO

Substrate	*Fgr*AAO	*Fox*AAO
	Products (% conversion)	Products (% conversion)
HMF	DFF (69%), FFCA (31%)	DFF (76%), FFCA (24%)
DFF	FFCA (49%)	FFCA (51%)
HMFCA	FFCA (84%)	FFCA (96%)
FFCA	0%	0%

It is important to note that *Fgr*AAO and *Fox*AAO are both able to convert HMF to FFCA, which is a step further than *Cgr*AAO [19]. The bacterial HMF oxidase is able to convert HMF fully to FFCA as well, but its use in industry may be hindered by its cofactor dependent mechanism [73, 75–77]. Full enzymatic conversion of HMF to FDCA is ideal, but *Fgr*AAO and *Fox*AAO show the possibility of an extension of current biocatalytic processes to FFCA with a single enzyme, instead of a complex co-factor dependent enzymatic cascade. Furthermore, FFCA itself has been used as precursor for biofuels, surfactants and resins [78].

### Saccharide oxidation

As indicated above, monosaccharides, and galactose in particular, are comparatively poor substrates for *Fgr*AAO and *Fox*AAO. Nonetheless, the observation of activity on galactose, mannose, and fructose deserves further comment. As listed in Table 1, the hexopyranoses galactose and mannose exhibit similar *k*_cat_ values, with the apparently greater selectivity for mannose originating from a ca. four-fold lower *K*_M_ value. The activity on fructose is likewise weak, but of particular interest as this 2-ketose presents two potentially oxidizable primary hydroxyl groups in the predominant furanose form.

*Galactose* Although the molecular basis for the specificity difference between *Fgr*AAO and *Fox*AAO and the archetypal *Fgr*GalOx is unclear in the absence of any three-dimensional substrate or product complexes of AA5 members [17–19, 41, 42, 47, 57, 79, 80], sequence analysis and molecular modeling provides some insight. In particular, there is literature precedent for docking ligand structures into models of AA5_2 variants for activity analysis [40, 81].

**Fig. 5:**
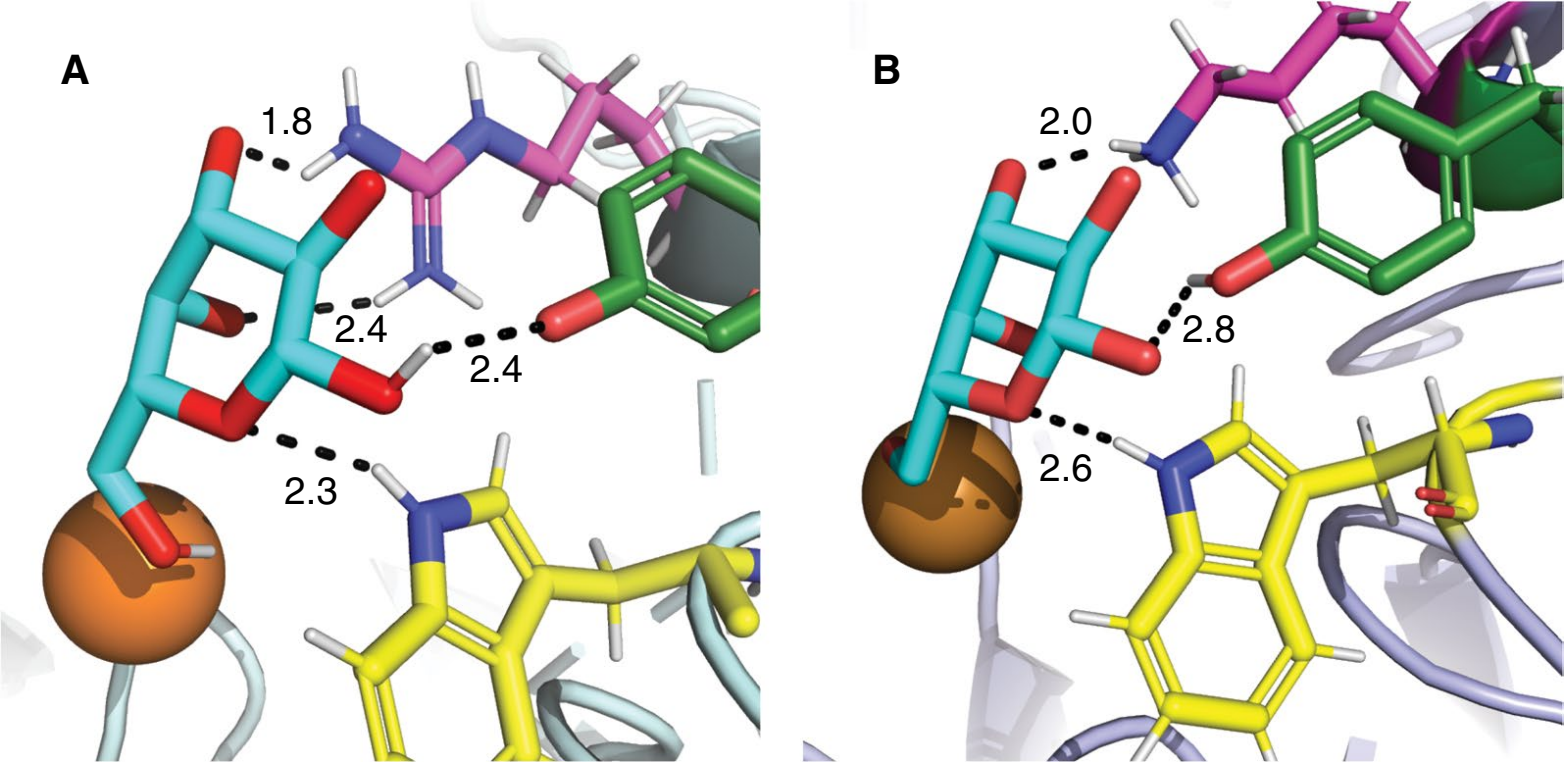
Molecular docking of galactose (cyan) in *Fgr*GalOx (PDB ID 1GOF) [17] (**A**) and a Phyre2 homology model of *Fgr*AAO (**B**) using AutoDock Vina as implemented in Chimera. Copper (dark orange), W290 (yellow), Y329 (green), R330/K330 (magenta). Ligand coordination is indicated, specifying the distances (black)

Foremost, substitution of R330 with lysine in *Fgr*GalOx has been shown to dramatically decrease catalytic efficiency towards galactose through a ninefold increase in *K*_M_ and a fivefold decrease in *k*_cat_ (Additional file 1: Table S3) [41]. This arginine, which is likewise replaced by lysine in *Fgr*AAO and *Fox*AAO (Fig. 2), has also been speculated to be involved in hydrogen bonding to the –OH groups at C3 and C4 of galactose by *Fgr*GalOx [39]. Molecular modeling (Fig. 5) supported this proposal and indicated that R330 is positioned in *Fgr*GalOx (PDB ID 1GOF) [17] to make two H-bonds with C3-OH and C4-OH (1.8 and 2.4 Å, respectively).

In contrast, when galactose is docked into a Phyre2 homology model of *Fgr*AAO (based on *Fgr*GalOx, PDB ID 2VZ1, as the template; 63% i.d., confidence value 100.0), only one potential H-bond is predicted between K330 and the C3-OH of galactose (2.0 Å). Although hypothetical, these structures implicate variation of this residue as a carbohydrate specificity determinant among AA5_2 members. Notably, *Cgr*AlcOx and *Cgr*AAO, which have similarly low activity on galactose as *Fgr*AAO and *Fox*AAO, also have a residue other than lysine at this position (Fig. 2). Furthermore, *Fgr*AAO and *Fox*AAO have catalytic efficiencies on galactose that are more similar to the *Fgr*GalOx mutant Des 3–2 (Q326E, Y329K and R330K) [40] (Additional file 1: Table S3), highlighting the importance of E326 and K330, which are both found natively in the homologs. The combination of mutations at distal positions (C383, Y436 and V494) might also contribute to the lower activity of *Fgr*AAO and *Fox*AAO, as these residues have been implicated in changing the catalytic efficiency towards galactose (Additional file 1: Table S3) [46–49].

*Mannose* The differences in catalytic constants between galactose and mannose are slight, and are thus difficult to rationalize. However, it is worth noting that this is the first report, to our knowledge, of an AA5_2 member that demonstrates a higher native catalytic efficiency on a non-galactosyl carbohydrate. The wild-type *Fgr*GalOx has no reported activity on mannose while the engineered variants M_1_ and M_3_ have relatively low specific activity compared to *Fgr*AAO and *Fox*AAO on this monosaccharide [42, 49]. Furthermore, the enzymes presented in this study have a twofold higher catalytic efficiently that the *Fgr*GalOx mutant H_1_ (Additional file 1: Table S3) [42]. These similarities could be caused by the analogous R330K substitution that is also present in the H_1_ [42] and M_3_ variants [42, 49]. Our attempts to model the binding of mannose to *Fgr*GalOx and *Fgr*AAO were inconclusive, precluding further discussion.

**Fig. 6:**
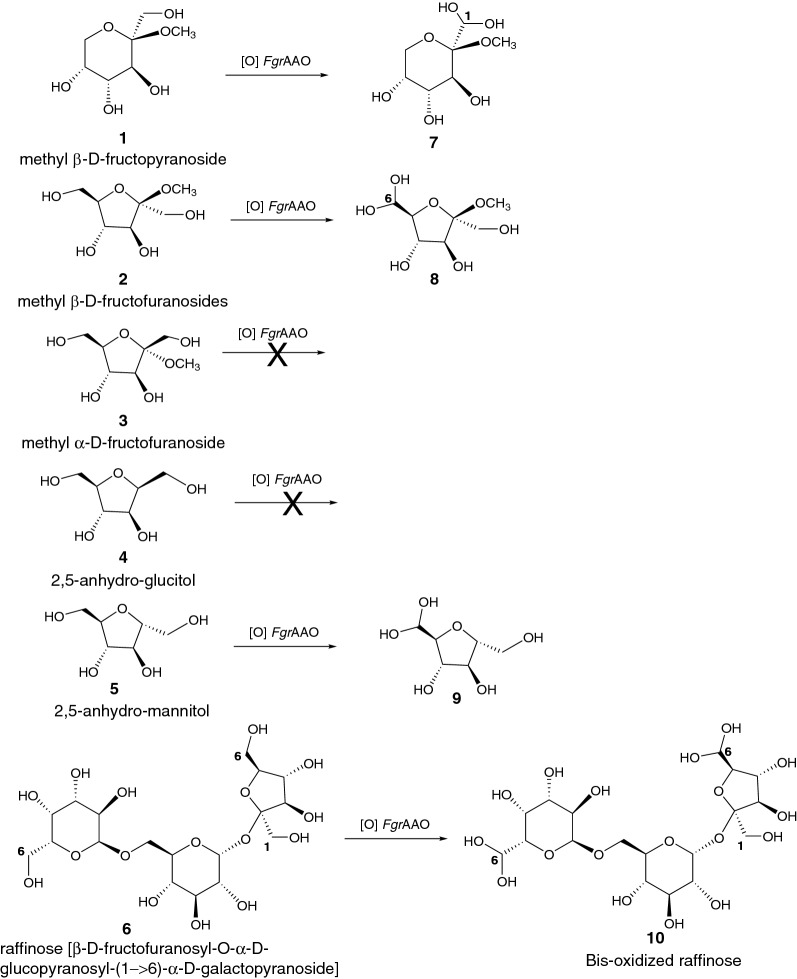
Summary of observed hydrated aldehyde (*gem*-diols) products from the oxidation of methyl fructosides, anhydro sugars and raffinose by *Fgr*AAO

*Fructose* The only other enzyme from AA5_2 with the ability to oxidize fructose is the engineered variant *Fgr*GalOx R330K [41], versus which *Fgr*AAO and *Fox*AAO have comparably low activities (Additional file 1: Table S3). Nonetheless, this raises the question of which of the primary hydroxyl groups of fructose might be oxidized by *Fgr*AAO and *Fox*AAO. Hence, we synthesized methyl β-d-fructopyranoside (**1**), methyl β-d-fructofuranoside (**2**) and methyl α-d-fructofuranoside (**3**) (Additional file 1: Figures S10–11) by Fisher glycosylation [82] to eliminate complications due to mutarotation of the free sugar (Fig. 6).

**Fig. 7:**
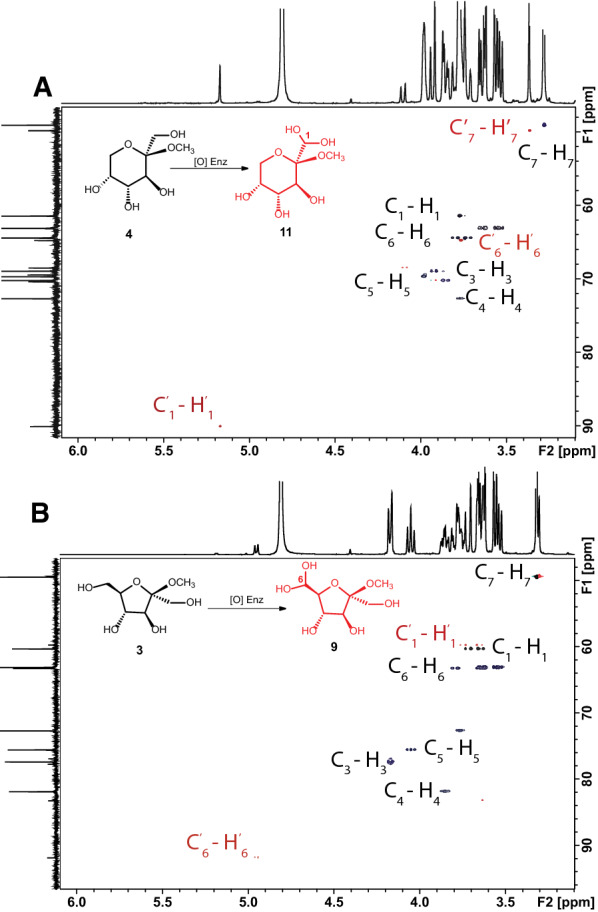
^1^H-^13^C HSQC NMR analysis of the oxidation of (**﻿A**) methyl β-D-fructopyranose (3) and (**B**) methyl β-D-fructofuranose (2) by *Fgr*AAO. Black crosspeaks correspond to ^1^J_C,H_-couplings in the substrates and the red crosspeaks correspond to ^1^J_C,H_-couplings in the products. Two carbon peaks at 63.1 ppm and 72.7 ppm were attributed to an impurity that is present in either the HRP, catalase or buffer solutions

1D (^1^H, ^13^C{^1^H}) and 2D NMR (HSQC and HMBC) spectroscopy revealed that the two enzymes produced identical oxidation products (Fig. 7, Additional file 1: Tables S6–S7 and Figures S12–17). The reaction of methyl β-d-fructopyranoside (**1**) with *Fgr*AAO and *Fox*AAO resulted in 19% and 17% conversion, respectively, as estimated by integration of ^1^H NMR spectra. The only product observed in both cases was the hydrated aldehyde (**7**), as confirmed by detailed 1D and 2D NMR analysis (Figs. 7, Additional file 1: S12 and S15A–17A). Specifically, no aldehyde signal was observed in the range 9–10 ppm in ^1^H NMR spectra, while a peak at 5.17 ppm corresponding to the C-H of a *gem*-diol [83] correlated to the quaternary carbon at C2, thus confirming the site of oxidation as the primary hydroxyl group of C1 (Additional file 1: Figures S15A and S17A). Turning our attention to the furanosides, methyl β-d-fructofuranoside (**2**) was converted to a single product (**8)** (Fig. 7, and Additional file 1: Figs. S13, S15B–17B) by *Fgr*AAO or *Fox*AAO with 16% and 11% conversion, respectively. In these cases, a distinct doublet was observed at 4.85 ppm, which was attributed to the C-H of a hydrated aldehyde. This doublet correlated to C5 in the HMBC spectrum, signifying that the primary hydroxyl of C6 was the site of oxidation of methyl β-d-fructofuranoside (Additional file 1: Figures S15B–17B).

On the other hand, the incubation of methyl α-d-fructofuranoside (**3**) with *Fgr*AAO or *Fox*AAO showed no indication of product formation (Additional file 1: Figures S14 and S15C–S17C). Thus, to further explore potential steric and stereochemical factors effecting enzymatic oxidation, 2,5-anhydro-d-glucitol (**4**) and 2,5-anhydro-d-mannitol (**5)** were tested as analogs of methyl α-d-fructofuranoside and methyl β-d-fructofuranoside, respectively. Notably, no oxidation was observed with 2,5-anhydro-glucitol, however 6% oxidation was observed for 2,5-anhydro-mannitol, which is concordant with our observations with the methyl fructofuranosides. The low degree of conversion of 2,5-anhydro-mannitol made full spectral assignment challenging, however, we were able to observe correlation peaks in the HSQC and HMBC (Additional file 1: Figure S18), which were consistent with oxidation of a single primary hydroxyl group to an aldehyde that was subsequently hydrated to the *gem*-diol (**9)**. In this context, it is important to note both primary hydroxyl groups of 2,5-anhydro-mannitol are equivalent, due to *C2* symmetry.

**Fig. 8:**
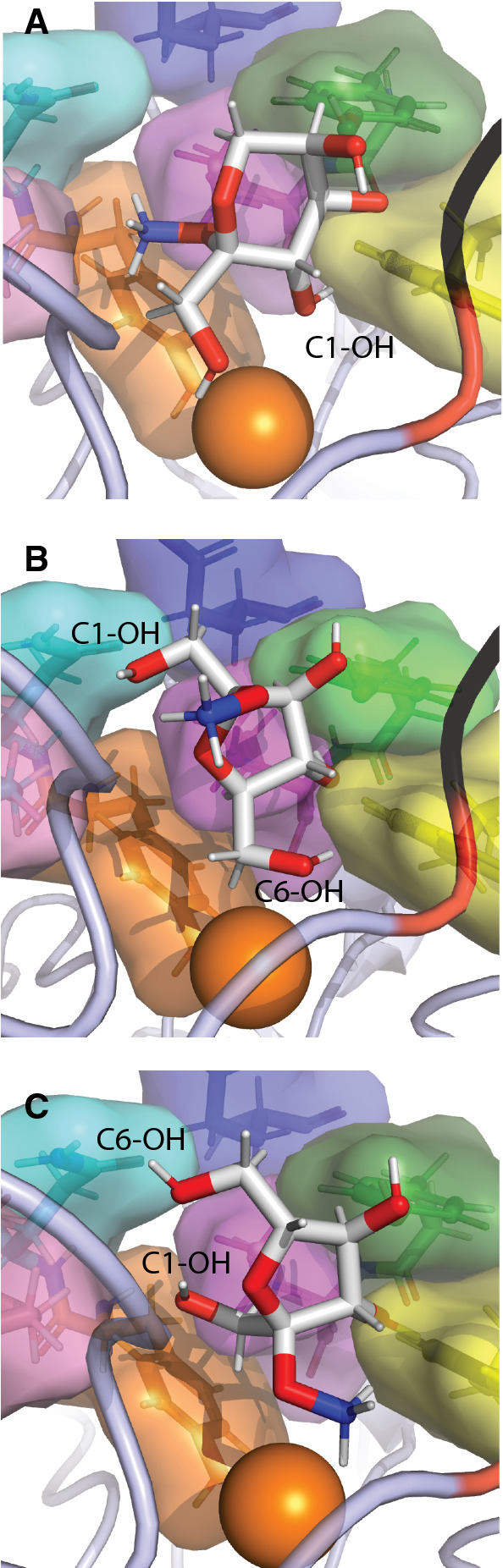
Molecular docking (AutoDock Vina as implemented in Chimera) of (**A**) methyl β-D-fructopyranoside (3), (**B**) methyl β-D-fructofuranoside (2) and (**C**) methyl α-D-fructofuranoside (1) in *Fgr*AAO. Copper (dark orange), methoxy group on sugar (dark blue), W290 (yellow), Y329 (green), K330 (magenta), E326 (blue), Y405 (orange), Q406 (cyan), P463 (pink), F194 (red), Q195 (black). C1-OH and C6-OH of each sugar are labeled

To assist in rationalizing these results, we modeled the binding of the three methyl fructosides (**1–3**) to a structural homology model of *Fgr*AAO. A structural model was obtained for methyl β-d-fructopyranoside (**1**) with C1–OH oriented towards the copper and the methoxy group positioned toward an active site pocket containing P463, Y405 and K330 (Fig. 8). However, in this model, Tyr272 was 3.7 Å away from the protons on the primary alcohol, suggesting a catalytically inactive pose. Using the same grid search area, a productive complex with methyl β-d-fructofuranoside (**2**) was obtained, in which the C6-OH was pointing towards the copper center and the anomeric methoxy group was pointing away from the substrate recognition residues and towards F464 and F194 (Fig. 8). In this model, the catalytic Tyr272 was located at a reasonable distance away from the abstractable hydrogen (2.0 Å). Most notably, the alternate structure, with the C1–OH oriented towards the copper, was not observed by modeling. Although hypothetical, these results are consistent with our experimental product analysis (Fig. 6). When docking was attempted with methyl α-d-fructofuranoside (**3**), only one model was produced with the methoxy pointed towards the copper center, which represents a non-productive binding mode and is consistent with experimental results (Fig. 8). Notably, this pose constitutes a flip of the furanose ring relative to that observed for methyl β-d-fructofuranoside, which places C-6 distal from the copper center. Together, these models suggest that binding modes that direct the anomeric methyl group into the protein are disfavored, thus rationalizing the observed specificity.

In light of the observed 6-*O*-oxidase activity on methyl β-d-fructofuranoside, as well as the oxidase activity observed on the classical GalOx substrate raffinose [β-d-fructofuranosyl-O-α-d-glucopyranosyl-(1 → 6)-α-d-galactopyranoside] (**6**) (Fig. 3), the potential for multiple oxidations of the trisaccharide was investigated by NMR spectroscopy. Thus ^1^H 1D-TOCSY, via selective irradiation of peaks at 6.19 ppm and 5.12 ppm that appeared following incubation with *Fgr*AAO, indicated oxidation and hydration of the C-6 hydroxyl groups of both galactose and fructose C6, respectively (full assignments of the proton and carbon spectra, for raffinose and the oxidation product using 1D (^1^H, ^13^C{^1^H}, TOCSY) and 2D (HSQC, HMBC and TOCSY) NMR, Additional file 1: Figures S19–S22, are provided in Table S8). Specifically, no peaks corresponding to oxidation of C1 on the fructosyl residue were observed. Based on the NMR analysis, 87% of unoxidized raffinose remained after incubation with *Fgr*AAO. Multiple products, including two mono-oxidized species and di-oxidized species (**10**), could be present, but due to low conversion no quantitative data could be obtained. The observation of the oxidation of both terminal primary hydroxyl groups in raffinose (Fig. 6) by *Fgr*AAO is consistent with the similar *k*_cat_/*K*_M_ values for the component monosaccharides, galactose and fructose. It would also appear that oxidation of C1 of the fructofuranosyl residue of raffinose is precluded sterically. The bis-oxidation of raffinose by *Fgr*AAO suggests a potential route to valorize raffinose as a chemical building-block, as has previously been explored with lactose and *Fgr*GalOx [33].

### Glycerol oxidation

Glycerol is a prochiral compound that is produced in significant quantities annually, including as a byproduct of the biodiesel industry [84]. Hence, there is significant interest in the valorization of glycerol by desymmetrization. It has been previously reported that the wild-type *Fgr*GalOx oxidizes glycerol stereoselectively to l-glyceraldehyde [85]. Moreover, *Fgr*GalOx variants have been integral to the desymmetrization of a key 2-alkynyl-glycerol derivative in the synthesis of a key antiviral drug [50]. On the other hand, we have also recently shown that *Cgr*AlcOx favors oxidation of glycerol to d-glyceraldehyde [86].

Here, comparative analysis indicates that *Fgr*AAO and *Fox*AAO both oxidize glycerol predominantly to produce L-glyceraldehyde, with enantiomeric ratios (*er*) of 93:7 and 91:9 (L-:D-), respectively (Additional file 1: Figures S23–S24). Under similar conditions, *Fgr*GalOx likewise favored the L-isomer, as expected, with an *er* of 96:4 (L-:D-), whereas the *er* for *Cgr*AlcOx was 10:90 (L-:D-). The similar stereoselectivities of the *Fusarium* AA5 CROs may be understood in terms of their conserved active-site motifs (Fig. 2). Nonetheless, the ability of these enzymes to selectively produce the unnatural and more valuable L-isomer may find biotechnological application. In this context, we note that *Fgr*GalOx is *ca.* twofold more active than *Fgr*AAO and *Fox*AAO.

## Conclusion

In summary, a detailed biochemical characterization of two orthologous CROs from *Fusarium graminearum* and *Fusarium oxysporum*, which segregate into a previously uncharacterized clade with AA5_2, revealed a distinct substrate specificity profile versus known GalOx [17, 25, 53–56], AlcOx [18, 26], and AAO [19] within the family. Despite strong conservation of active-site architecture compared to the archetypal *Fgr*GalOx, both *Fgr*AAO and *Fox*AAO have a novel native preference for mannose and fructose over galactose or galactosides, and a clear preference for aryl alcohols, specifically 3-methoxybenzyl alcohol and veratryl alcohol. Unlike *Cgr*AlcOx [18], *Fgr*AAO and *Fox*AAO are poorly active on unactivated alkanols, with the exception of the aforementioned saccharides and glycerol. While the nomenclature of oxidases is fraught with difficulty due to overlapping definitions of EC 1.1.3.7 (aryl alcohol oxidase), EC 1.1.3.13 (alcohol oxidase), and EC 1.1.3.47 (5-(hydroxymethyl) furfural oxidase), detailed kinetic data (Table 1 and Fig. 3) support denoting these *F. graminearum* and *F. oxysporum* CROs as aryl alcohol oxidases. High *K*_M_ values and low specificity constants (*k*_cat_/*K*_M_ values) do not suggest that galactose 6-oxidation (EC 1.1.3.9) is the natural activity of these enzymes. Finally, we note that the physiological substrate of these enzymes is currently unknown and may not be represented in our panel of compounds.

In this context, it is notable that *Fgr*AAO and *Fox*AAO group together with orthologs from *F. verticillioides* and *F. subglutinans* in a clade previously designated as “*gaoB*” (galactose oxidase B) [17] (Fig. 1). However, the *F. verticillioides* and *F. subglutinans* gene products have not been biochemically characterized [59]. Our kinetic data on *Fgr*AAO and *Fox*AAO, like data on the *Colletotrichum* homologs [18, 19, 26], indicate that caution is warranted in extending functional predictions in AA5_2 on the basis of bioinformatics alone. Indeed, our analysis would suggest that the *F. verticillioides* and *F. subglutinans* orthologs are likely to also be predominant aryl alcohol oxidases. Within this clade, an arginine to lysine substitution in the active site may be a key specificity determinant.

Finally, the ability of *Fgr*AAO and *Fox*AAO to oxidize HMF and derivatives, as well as glycerol, suggests possible applications of these CROs in biocatalysis. Like FAD-dependent oxidases, CROs use molecular oxygen as a co-substrate, thus avoiding the requirement for co-factor regeneration of NAD-dependent oxidases. At the same time, the facile production of AA5 CROs in *P. pastoris* and *E. coli* enables further tuning of specificity for biocatalytic applications [42, 49, 50, 64], which will be further informed by enzyme structure–function relationships, including those described here. Indeed, the replacement of chemical oxidants with potentially greener biocatalysts is a topic of considerable contemporary interest.

## Methods

### Chemicals and enzymes

Wild-type galactose oxidase from *Fusarium graminearum* and alcohol oxidase from *Colletotrichum graminicola* were produced in *Pichia pastoris* and purified as previously described [18, 64]. Ultrapure water was used for the preparation of all buffers and stock solutions unless stated otherwise. Catalase from bovine liver (2000–5000 units per mg protein, Sigma) and horseradish per-oxidase (*R*z > 3300 units per mg, Bio Basic Canada Inc.), obtained as lyophilized powders, were used as received. Other substrates and reagents were purchased from commercial sources (Sigma-Aldrich, VWR or Fisher) and used without further purification.

### Sequence and bioinformatic analysis

Sequences of *Fgr*AAO (GenBank XP_011322138) and *Fox*AAO (GenBank XP_018246910) were taken from the National Center for Biotechnology Information (NCBI). Percent identity analysis was performed using the full-length sequences of *Fgr*AAO, *Fox*AAO and other AA5_2 members from the *Fusarium* and *Colletotrichum* genus using MatGat [87]. An alignment using the online Genome Net CLUSTALW tool was performed with the two new enzymes presented in this study and compared to the characterized galactose oxidase from *Fusarium graminearum* (*Fgr*GalOx) (GenBank: P0CS93.1), alcohol oxidase (*Cgr*AlcOx) (GenBank: EFQ30446.1) and aryl oxidase (*Cgr*AAO) (GenBank: EFQ27661.1) from *Colletotrichum graminicola*. In addition, Phyre2 [88] was used to make three-dimensional homology models for *Fgr*AAO and *Fox*AAO. The structural models were aligned to the crystal structures of *Fgr*GalOx (PDBID 1GOF) [17] and *Cgr*AlcOx (PDBIID 5C86) [18] for spatial amino acid comparison to give further support to the alignment.

In addition, two AA5_1 sequences and 62 sequences of AA5_2 members from a variety of fungal species were extracted from the public CAZY database [13] (February 2021) and aligned using MUSCLE [89]. Where present, signal sequences and additional domains, such as carbohydrate-binding modules, were removed. Any redundant sequences were excluded and the resulting catalytic domain sequences were realigned. A maximum-likelihood phylogenetic tree was estimated by RAxML version 8.2.12 [90] using 47 sequences as inputs on the CIPRES gateway [91] with automatic bootstrapping terminating at 650 bootstrap replicates. The resulting phylogeny was visualized with FigTree (http://tree.bio.ed.ac.uk/software/figtree/).

### DNA cloning

cDNA encoding *Fgr*AAO (GenBank ID XP_011322138) and *Fox*AAO (GenBank ID XP_018246910) without the predicted native signal peptide and including a C-terminal His6 tag-encoding sequence were commercially synthesized in a codon-optimized form. The sequences were cloned into the *P. pastoris* expression vector pPICZα-A using the *Eco*RI and *Xba*I restriction sites flush with the sequence encoding the *S. cerevisiae* α-factor signal peptide by Genescipt. The resulting constructs were transformed into chemically competent *E. coli* DH5α by heat shock.

In both cases, transformants were grown overnight at 37 °C on Luria–Bertani low-salt (LBLS) agar plates and selected against 25 μg mL^−1^ Zeocin (Invitrogen). Surviving colonies were picked and grown overnight at 37 °C in 5 mL LBLS medium with 25 μg mL^−1^ Zeocin. Plasmids from the overnight cultures were extracted using a commercial mini-prep kit (Geneaid, New Taipei City, Taiwan). The presence of the pPICZα-A-*Fgr*AAO and pPICZα-A-*Fox*AAO constructs in positive clones was checked by agarose (1%; w/v) gel electrophoresis and the correct insertion of both genes into the corresponding vectors was verified by DNA sequencing. Transformation of constructs into *P. pastoris* X33 was performed by digesting 6.5–14 μg of plasmid DNA for 4 h at 37 °C with *Pme*I (New England Biolabs). The linearized plasmid was purified using an ion exchange column (Omega). The resulting digested plasmids were transformed into electrocompetent *P. pastoris* X33 cells prepared on the same day [92].

Transformants were grown on yeast extract peptone dextrose (YPDS) agar plates for 2 days at 30 °C and selected against 100 or 500 μg mL^−1^ Zeocin. Four colonies selected from 500 μg mL^−1^ Zeocin plates were inoculated into 15 mL sterile conical tubes containing 2 mL of YPD and grown for 6 h at 30 °C shaking at 200 rpm. At OD ~ 0.15, 200 μL of the growing culture was transferred to 10 mL of buffered complex glycerol medium (BMGY) and grown in a shaking incubator overnight at 30 °C and 200 rpm. Cells were then pelleted by centrifugation at 3000 g﻿ for 10 min at 25 °C, the BMGY medium was discarded and replaced with 4 mL of buffered complex methanol medium (BMMY) containing 0.5% (v/v) methanol. The cultures were shaken at 200 rpm over 3 days at either 25 or 16 °C with regular feeding of 0.5% methanol every 24 h to ensure continued protein expression. Secreted proteins were separated from the cells by centrifugation at 4000 rpm for 10 min and protein production was monitored using SDS-PAGE. The clone yielding the highest amount of protein was retained for large-scale production.

### Large-scale protein production

Single colonies of *P. pastoris* X33 expressing clones were individually streaked onto agar plates containing 500 μg mL^-1^ $\mathrm{\mu }$ of the antibiotic Zeocin and grown for 2 days in the dark in a 30 °C incubator. Precultures containing 4 mL of YPD and 500 μg mL^−1^ Zeocin were inoculated using a single colony and shaken at 30 °C at 250 rpm for 10 h. Biomass production was initiated by the addition of the preculture into 1 L of BMGY media shaken in 4 L beveled flasks at 30 °C at 250 rpm overnight. Once the BMGY cultures reached an OD_600_ of 6–12, the cells were harvested by centrifugation at 3000*g* for 15 min at room temperature. The cells were then resuspended using 400 mL of BMMY media supplemented with 0.5 mM copper sulfate containing 3% methanol and transferred to 1 L flasks with a foam cap. The flasks were shaken at 250 rpm at 16 °C for three days. The cultures were fed 1% (v/v) methanol every 24 h to maintain continued expression of the recombinant protein. On the last day of methanol induction, the desired proteins were separated from the cells by centrifugation at 3000*g* for 30 min at 4 °C. The supernatant was quickly decanted, filtered through 0.45 μm membrane and stored at 4 °C until purification.

### Protein purification

The pH of the liquid medium which contained *Fgr*AAO or *Fox*AAO was raised before purification to 7.5–8.0 by the dropwise addition of 1 M NaOH with stirring. The medium was the filtered through a 0.45 μm membrane and allowed to equilibrate for at least 12 h at 4 °C. The supernatant was allowed to pass through a 5 mL pre-packed Ni–NTA column, pre-equilibrated with 50 mM sodium phosphate buffer at pH 7.5 with 300 mM NaCl with 10 mM imidazole at 5 mL/min. The column was washed with 5 column volumes of equilibration buffer containing 50 mM sodium phosphate buffer at pH 7.5 with 300 mM NaCl and 10 mM imidazole at 4.5 mL/min. Proteins were eluted with a linear gradient of 2% to 100% of 500 mM imidazole in a 50 mM sodium phosphate buffer with 300 mM NaCl at 5 mL/min. The total elution volume was 125 mL collected in 1 mL fractions. Both *Fgr*AAO and *Fox*AAO eluted at ~ 140 mM imidazole. The proteins were then concentrated using a 30,000 MWCO Vivaspin centrifugal concentrator and 0.5–1 mL was loaded onto a Superdex 75 size-exclusion column pre-equilibrated with 50 mM sodium phosphate buffer at pH 7.5 at 1 mL/min. A total volume of 200 mL of equilibration buffer was used through the column at 1 mL/min. *Fox*AAO eluted at 102 mL as one singular peak, while *Fgr*AAO eluted as two peaks at 72 and 104 mL. SDS-PAGE was performed using pre-cast 4–20% (w/v) polyacrylamide gel in the presence of 2% (w/v) SDS under reducing conditions. Proteins were visualized using Coomassie blue R-250. Protein concentrations were determined by measuring A_280_. The extinction coefficients were calculated using the ProtParam tool on the ExPASy server.

### Analytical protein deglycosylation

The presence of protein glycosylation on *Fgr*AAO and *Fox*AAO was assessed by treatment with N-glycosidase F from *Flavobacterium meningosepticum* (PNGaseF, New England Biolabs). Deglycosylation experiments were performed under denaturing condition by adding 3 μg of protein to 10X Glycoprotein Denaturing Buffer and heated for 10 min at 100 °C. The samples were subsequently diluted to 20 μL with GlycoBuffer 2 and tertigol-type NP-400 detergent. Finally, 1 μL of PNGaseF was added to the sample and incubated for 1 h at 37 °C. Changes in protein mobility was assessed by SDS-PAGE stained with Coomassie blue R-250.

### pH and temperature profile

Enzyme activity across a wide range of pH values was determined using phosphate citrate (pH 4.0–7.0), sodium phosphate (pH 5.5–8.5) and glycine–NaOH (pH 8.5–11.0) buffers. Enzyme activity was measured using the HRP–2,2’-azinobis(3-ethylbenzthiazoline-6-sulfonic acid) (ABTS)-coupled assay with 300 mM of galactose at 25 °C [64]. The optimum temperature was determined using the same coupled assay using 50 mM sodium phosphate buffer at pH 7.5 with 1 μg mL^−1^ as the final enzyme concentration and 300 mM galactose as the substrate at temperatures ranging from 15 °C to 80 °C.

### Temperature stability

Temperature stability of both *Fgr*AAO and *Fox*AAO was determined by diluting the stock protein in 50 mM sodium phosphate buffer at pH 7.5 to obtain a final protein concentration of 0.2 mg mL^−1^ for both proteins studied. The diluted protein was then pre-incubated in a thermocycler at 30 °C, 39 °C, 49 °C and 60 °C. Samples were taken out at different time interval and the activity of the proteins was measured using the HRP-ABTS coupled assay [64] with 300 mM galactose as the substrate at 30 °C. 1 μg mL^−1^ was the final enzyme concentration in the reactions.

### Enzyme kinetics

The colorimetric HRP-ABTS coupled assay was used to determine the kinetics of the enzymatic oxidation of substrates. The oxidation of the alcohol group on the substrates by AA5 enzymes consumes 1 equivalent of O_2_ and produces 1 equivalent of H_2_O_2_. The oxidation of ABTS (*λ*_max_ = 420 nm, *ε* = 36,000 M^−1^ cm^−1^) is catalyzed by the enzymes HRP using 2 equivalents of H_2_O_2_ [64]. This assay was optimized for sensitivity and linearity.

The activity of *Fgr*AAO and *Fox*AAO was surveyed on a variety of substrates using this coupled assay comprising 50 mM sodium phosphate buffer at pH 7.5, 0.46 mM ABTS and 30 U/mL of HRP at 25 °C. The initial substrate screen included carbohydrates, polyols, diols and primary alcohol substrates at 300 mM, polysaccharides at 2.5 mg mL^−1^, benzyl alcohol and galactitol at 30 mM, methyl glyoxal, aryl alcohols and furans at 5 mM and 10 mM for secondary alcohols. One unit of AA5 enzyme activity was defined as the amount of enzyme required to oxidize 2 μmol of ABTS per minute, which is equivalent to the consumption of 1 μmol of oxygen per minute.

To determine Michaelis–Menten parameters of *Fgr*AAO and *Fox*AAO, different concentrations of substrates were used over the range of 10–2500 mM for galactose, 10–4000 mM for fructose, 5–3000 mM for mannose, 25–7000 mM for glycerol, 10–250 mM for benzyl alcohol, 0.5–13 mM for cinnamyl alcohol, 1–200 mM for *p*-anisyl alcohol for *Fgr*AAO and 1–100 mM for *Fox*AAO, 0.25–100 mM for *m*-anisyl alcohol for *Fgr*AAO and 0.25–50 mM for *Fox*AAO, 0.25–10 mM for veratryl alcohol, 1–80 mM for HMF, 0.25–20 mM for HMFCA and 0.1–40 mM for DFF for *Fgr*AAO and 0.5–20 mM for *Fox*AAO. The reactions were performed at 35 ^o^C in 50 mM sodium phosphate buffer at pH 7.5 with 0.46 mM ABTS, 30 U/mL of HRP and using 2.1 to 28 pmol of purified *Fgr*AAO and *Fox*AAO. Data were fit with the Michaelis − Menten equation using OriginPro software (OriginLab 9.55).

### Computational docking studies

Molecular docking simulations were performed using the CHIMERA software from UCSF Resource for Biocomputing, Visualization, and Informatics [93]. The *Fgr*GalOx crystal structure (PDB 1GOF) and a Phyre model of *Fgr*AAO were used to generate the receptors for simulations. Ligands were either extracted from other PDB files or build from components extracted from other PDB files. Ligands and receptors were first prepared for docking in chimera by adding hydrogens and assigning proper protonation states. The docking simulation itself was performed using Autodock VINA, run within CHIMERA, with the AMBER03 force field [94]. Appropriate simulation cells were defined for the respective docking simulations. For docking of galactose and mannose, a 7 Å cell with the copper atom bordering the z-coordinate edge was chosen. Galactose gave a reasonable binding pose with both *Fgr*GalOx and *Fgr*AAO, while mannose lowest energy structures produced the sugar in unproductive binding positions. For the docking of methyl α/β-fructofuranosides and methyl β-fructopyranoside, a 6 × 7 × 7 Å cell was selected with the copper atom bordering the z-coordinate edge. Reasonable functional binding poses were calculated for methyl β-fructofuranosides and methyl β-fructopyranoside, and an unproductive for catalytic turnover binding structure was calculated for methyl α-fructofuranosides. The created receptor–ligand complex structures were further processed using the PyMOL software from Schrodinger LLC involving the identification of ligand–receptor interaction and for the determination of distances.

### Synthesis of methyl α/β-d-fructofuranosides and methyl β-d-fructopyranoside

The 1-O-methyl d-fructose derivatives were prepared according to a modified procedure [82]. To a solution of D-fructose (1.00 g, 0.111 mol) in MeOH (50 mL) 50 μl of acetyl chloride (AcCl) was added at RT and stirred for 25 h. The reaction was neutralized with Dowex 66 Free Base resin, filtered, washed with MeOH and concentrated. Half the crude mixture was purified by anion exchange chromatography (Dowex 1 × 8 200–400, ^−^OH form; distilled water as the mobile phase), which yielded three products: methyl α-d-fructofuranoside (0.193 g, 39%, R_f_ 0.58 [6:4:1, EtOAc-*i*-PrOH-H_2_O]), methyl β-d-fructofuranoside (0.064 g, 13%, R_f_ 0.46), methyl β-d-fructopyranoside (0.084 g, 17%, R_f_ 0.34) and a mixture of methyl α/β-d-fructofuranoside (0.004 g, 0.8%) and methyl β-d-fructofuranoside/ β-d-fructopyranoside (0.141 g, 29%) all as clear gels, giving a total purification yield of 99%.

Methyl α-d-fructofuranoside: ^1^H NMR (400 MHz, D_2_O): *δ* = 3.22 (s, 3H, H_7_), 3.68 (dd, *J* = 32.6, 12.3 Hz, 4H overlapped with H_6_, H_1_), 3.72 (m, 4H overlapped with H_1_, H_6_), 3.85 (m, 1H, H_4_), 4.05 (t, *J* = 7.8 Hz, 1H, H_5_), 4.17 (d, *J* = 8.2 Hz, 1H, H_3_). ^13^C{^1^H} NMR (100.6 MHz, D_2_O): δ 49.5 (C_7_), 60.5 (C_1_), 63.3 (C_6_), 75.7 (C_5_), 77.6 (C_3_), 82.0 (C_4_), 104.4 (C_2_)

 Me﻿thyl β-d-fructofuranoside: ^1^HNMR (400 MHz, D_2_O): δ 3.32 (s, 3H, H_7_), 3.73 (dd, *J* = 49.7, 12.8 Hz, 4H overlapped with H_6_, H_1_), 3.75 (dd, *J* = 52.6, 12.9 Hz, 4H overlapped with H_1_, H_6_), 3.96 (m, 2H, H_4_ and H_5_), 4.10 (m, 1H, H_3_). ^13^C{^1^H} NMR (100.6 MHz, D_2_O): δ = 48.8 (C_7_), 58.4 (C_1_), 61.9 (C_6_), 77.9 (C_5_), 80.7 (C_3_), 83.8 (C_4_), 108.9 (C_2_). 

Methyl β-d-fructopyranoside: ^1^HNMR (400 MHz, D_2_O): δ = 3.29 (s, 3H, H_7_), 3.76 (dd, *J* = 31.4, 13.0 Hz, 4H overlapped with H_1_, H_6_), 3.77 (s, 4H overlapped with H_6_, H_1_), 3.85 (m, 1H, H_4_), 3.92 (d, *J* = 10.0 Hz, 1H, H_3_), 3.97 (m, 1H, H_5_). ^13^C{^1^H} NMR (100.6 MHz, D_2_O): δ 49.1 (C_7_), 61.6 (C_1_), 65.2 (C_6_), 68.6 (C_3_), 69.3 (C_5_), 70.0 (C_4_), 101.2 (C_2_).

### Enzyme product analysis

#### Oxidation of methyl α/β-d-fructofuranosides and methyl β-d-fructopyranoside

Reactions containing 20 mg of substrate (methyl α/β-d-fructofuranosides and methyl β-d-fructopyranoside) and 1 mg mL^−1^ of both catalase and HRP were initiated by the addition of 300 μg of purified *Fgr*AAO and *Fox*AAO in a final volume of 1 mL (50 mM sodium phosphate buffer, pH 7.5). Reactions were stirred at 400 rpm at room temperature for 16 h, at which time the enzymes were removed by ultrafiltration (10 kDa cut-off Centricon-Millipore, Billerica, MA, USA). The products were collected, frozen in liquid nitrogen and lyophilized for 4 days. The resulting powders were resuspended in D_2_O and a preliminary NMR analysis was conducted. The samples were subsequently lyophilized for 24 h and a second reaction was performed under the previously mentioned conditions with 500 μg of purified enzyme added. The reaction was stirred for 25 h and the same work-up procedure was followed.

In this and subsequent product analyses, NMR spectra were acquired on a Bruker AV III HD 400 MHz spectrometer equipped with a BBFO smart probe. ^1^H and ^13^C spectra were calibrated using an internal standard of acetone (0.34 M; 2.22 ppm and 30.89 ppm, respectively). Peak integration values were used to determine the extent of substrate  conversion to product(s).

### Oxidation of 2,5-anhydromannitol and 2,5-anhydroglucitol

Reactions containing 20 mg of substrate (2,5-anhydro-d-mannitol and 2,5-anhydro-d-glucitol) and 1 mg mL^−1^ of both catalase and HRP (dissolved in D_2_O) were initiated by the addition of 700 μg of purified *Fgr*AAO in a final volume of 1 mL (50 mM sodium phosphate buffer, pH 7.5 previously lyophilized and resuspended in D_2_O). Reactions were stirred at 400 rpm at room temperature for 24 h, at which time the enzymes were removed by ultrafiltration (5 kDa cut-off Centricon-Millipore, Billerica, MA, USA).

### Oxidation of raffinose

Reactions containing 20 mg of raffinose and 1 mg mL^−1^ of both catalase and HRP were initiated by the addition of 800 μg of purified *Fgr*AAO in a final volume of 1 mL (50 mM sodium phosphate buffer, pH 7.5). A control reaction without *Fgr*AAO was performed using the same protocol. Reactions were stirred at 400 rpm at room temperature for 25 h, at which time the enzymes were removed by ultrafiltration (5 kDa cut-off Centricon-Millipore, Billerica, MA, USA). The products were collected, frozen in liquid nitrogen and lyophilized for 3 days. The resulting powders were resuspended in D_2_O for NMR analysis.

### Oxidation of HMF, DFF, HMFCA, FDCA and furfural

Reactions containing 10 mM of substrate (HMF, DFF, HMFCA, FDCA and furfural) and 1 mg mL^−1^ of both catalase and HRP were initiated by the addition of 60 μg of purified *Fgr*AAO and *Fox*AAO in a final volume of 1 mL (50 mM sodium phosphate buffer, pH 7.5). For each reaction a negative control was performed with identical conditions omitting the purified enzyme. Reactions were stirred at 400 rpm at room temperature for 16.5 h, at which time the enzymes were removed by ultrafiltration (5 kDa cut-off Centricon-Millipore, Billerica, MA, USA). D_2_O was added to the filtrate to a final composition of 10% (v/v). ^1^H NMR spectra were collected with water suppression (4.7 ppm) using a standard pre-saturation pulse sequence. Chemical shifts were calibrated to the internal HOD peak (4.7 ppm). Standards of all substrates were used to identify distinct chemical shifts for each molecule.

### Oxidation of glycerol

Reaction containing 0.54 M glycerol and 1 mg mL^−1^ of both catalase and HRP were initiated by the addition of 800 μg of purified *Fgr*GalOx, *Cgr*AlcOx, *Fgr*AAO and *Fox*AAO in a final volume of 1 mL (50 mM sodium phosphate buffer, pH 7.0–8.0). Reactions were left at room temperature for 24 h, at which time the enzymes were removed by ultrafiltration (5 kDa cut-off Centricon-Millipore, Billerica, MA, USA). 20 mg of 2,4-dinitrophenyl hydrazine was added to the Eppendorf and the reaction mixture was incubated in a heat block at 50 ℃ for 6 h. A TLC plate was used to check formation of desired product (*R*_f_ = 0.45 100% EtOAc). Subsequently, the solution was purified via preparatory TLC and the desired hydrazone was mechanically isolated and dissolved in MeOH. The solution was then filtered and concentrated. The composition of the purified glyceraldehyde-hydrazones was analyzed on HPLC (3 μL injection, Chiracel® IA-3). Eluents used for HPLC methods, water with 0.1% formic acid (A) and methanol (B) were LC–MS grade (Optima, Fisher). For separation between l-and d-glyceraldehyde-hydrazones an isocratic method using 60% A, 40% B with a flow rate of 0.65 mL/min was used with a 12 min stop time with UV detection at 360 nm. l-glyceraldehyde-hydrazone eluted at 2.30 min and d-glyceraldehyde-hydrazone eluted at 2.76 min. ESI mass spectra were also collected in positive mode scan for *m*/*z* 95–500 running at 0.8 s/cycle drying gas = 5.0 L/min, nebulizer pressure = 50 psi, gas temperature = 300 °C, capillary voltage = 4000 V.

## Supplementary Information

**Additional file 1:** Supplementary Tables S1-S8 and Supplementary Figures S1-S24.

## Data Availability

All data generated or analyzed during this study are included in this published article and its supplementary information files. All nucleotide sequence, protein sequence, and protein structural information used in this work was extracted from existing accessions in public databases, e.g., GenBank and the Protein Data Bank.
