# Supplementary material for: Two Fusarium copper radical oxidases with high activity on aryl alcohols
Source: Biotechnol Biofuels. 2021 Jun 16;14:138. doi: 10.1186/s13068-021-01984-0 (PMC8207647; doi:10.1186/s13068-021-01984-0)
Supplement: Supplementary file 1 — Additional file 1: Supplementary Tables S1-S8 and Supplementary Figures S1-S24. [file 13068_2021_1984_MOESM1_ESM.pdf]

Supporting Tables

Table S1. Pairwise sequence ID/similarity matrix between *Fgr*AAO and *Fox*AAO and other AA5_2 enzymes. Sequence similarity is highlighted in turquoise and sequence identity is highlighted in blue.

	1	2	3	4	5	6	7	8	9	10
1. *Fgr*AAO (XP_011322138)		91.3	58.0	55.4	54.7	78.2	79.1	54.8	42.2	35.9
2. *Fox*AAO (XP_018246910)	95.1		57.3	54.7	53.9	82.8	84.2	54.0	41.2	35.9
3. *Fgr*GalOx (AAO95371)	72.1	70.6		77.7	90.0	59.2	59.1	76.9	37.6	38.8
4. *Fox*GalOx (AHA90705)	68.1	67	86.9		81.3	60.7	60.6	94.9	36.7	39.5
5. *Fsa*GalOx (AIR07394)	67.7	66.4	92.6	91.0		59.9	59.4	80.2	35.4	39.2
6. *Fsu*AA5 (ADG08187)	81.7	84.7	71.9	76.1	74.7		96.9	60.6	35.8	36.8
7. *Fve*AA5 (ADG08188)	82.3	85.4	72.0	75.3	74.4	98.4		60.3	35.8	36.8
8. *Fsu*GalOx (AJE27923)	68.6	67.3	86.5	97.5	90.6	76.1	75.9		36.2	39.3
9. *Cgr*AlcOx (EFQ30446)	57.2	57.1	52.1	50.5	49.2	50.2	50.1	50.7		33.4
10. *Cgr*AAO (EFQ27661)	51.1	50.9	54.4	56.8	55.4	54.1	54.3	56.1	47.3	

Table S2. Initial activity screens* of *Fgr*AAO and *Fox*AAO

	Substrate	Specific Activity (μmol.min^{-1}.mg^{-1})	
		***Fgr*AAO**	***Fox*AAO**
**Carbohydrates**	D-Galactose (300mM)	2.99 ±0.06	2.51 ±0.10
	D-Lactose (300mM)	2.59 ±0.03	2.29 ±0.42
	Raffinose (300mM)	1.41 ±0.04	1.24 ±0.23
	D-Mannose (300mM)	9.40 ±0.37	5.68 ±0.25
	Melibiose (300mM)	2.00 ±0.02	0.60 ±0.04
	D-Fructose (300mM)	3.00 ±0.07	3.33 ±0.12
	D-Xylose (300mM)	0.30 ±0.03	0.09 ±0.07
	D-Glucose (300mM)	0.15 ±0.01	0.09 ±0.00
	Sucrose (300mM)	0.22 ±0.01	0.25 ±0.00
	D-Ribose (300mM)	n.m.§	n.m.§
	L-Arabinose (300mM)	n.m.§	n.m.§
	Xyloglucan (2.5 mg.ml^{-1})	0.01 ±0.00	n.m.§
	Carab Galactomannan (2.5 mg.ml^{-1})	0.09 ±0.02	0.04 ±0.01
	Levan (2.5 mg.ml^{-1})	0.01 ±0.00	n.m.§
	Inulin (2.5 mg.ml^{-1})	n.m.§	n.m.§
	Fructo Oligos (2.5 mg.ml^{-1})	0.03 ±0.00	n.m.§
	Beta Mannan (2.5 mg.ml^{-1})	n.m.§	n.m.§
**Polyols**	Glycerol (300mM)	1.53 ±0.02	1.24 ±0.02
	Sorbitol (300mM)	0.09 ±0.00	0.07 ±0.00
	Galactitol (300mM)	0.04 ±0.00	0.03 ±0.00
	Mannitol (300mM)	0.11 ±0.00	0.07 ±0.00
**Diols**	1,3-Propanediol (300mM)	0.26 ±0.00	0.20 ±0.00
	1,2-Propanediol (300mM)	0.23 ±0.00	0.15 ±0.00
	1,4-Butanediol (300mM)	0.34 ±0.00	0.25 ±0.01
**Aldehyde**	Methyl Glyoxal (5mM)	0.25 ±0.04	0.07 ±0.01
**Primary alcohols**	Ethanol (300mM)	0.06 ±0.00	0.04 ±0.00
	Methanol (300mM)	0.05 ±0.00	0.03 ±0.00
	1-Butanol (300mM)	0.09 ±0.00	0.05 ±0.00
	Hexanol (300mM)	0.03 ±0.00	0.08 ±0.00
	2-Phenylethanol (1mM)	0.01 ±0.00	n.m.§
**Secondary alcohols**	1-Phenylethanol (1mM)	n.m.§	n.m.§
**Aryl alcohols**	Benzyl alcohol (5mM)	1.23 ±0.03	1.23 ±0.02
	Cinnamyl alcohol (5mM)	4.90 ±0.13	5.00 ±0.08
	4-methoxybenzyl alcohol (5mM)	0.89 ±0.02	0.98 ±0.02
	3-methoxybenzyl alcohol (5mM)	12.21 ±0.09	16.41 ±0.25
	Coniferyl alcohol (5mM)	n.m.§	n.m.§
	Veratryl alcohol (5mM)	20.13 ±0.42	25.19 ±0.89
	4-hydroxybenzyl alcohol (5mM)	1.05 ±0.09	0.45 ±0.10
	Vanillyl alcohol (5mM)	0.01 ±0.00	n.m.§
**Furans**	HMF (5mM)	3.00 ±0.09	3.46 ±0.13
	DFF (5mM)	0.91 ±0.04	0.98 ±0.18
	HMFCA (5mM)	1.82 ±0.07	2.64 ±0.35
	FFCA (5mM)	n.m.§	n.m.§

*Measurements were performed in triplicate at 25 ˚C in 50 mM sodium phosphate buffer, pH 7.5, using the HRP/ABTS assay. Activities were monitored using concentrations indicated within parentheses for each substrate.

§No activity detected with a specific activity limit of detection of 9×10^{-4} $\mu mol.min^{-1}.mg^{-1}$ using 1 nmole of purified enzyme.

Table S3. Comparison of catalytic parameters of *Fgr*AAO and *Fox*AAO with other enzymes acting on galactose, fructose and mannose*

Enzyme	*Galactose*			*Fructose*			*Mannose*			References
	**K_M (mM)**	**k_{cat} (s^{-1})**	**k_{cat}/K_M ($M^{-1}.s^{-1}$)**	**K_M (mM)**	**k_{cat} (s^{-1})**	**k_{cat}/K_M ($M^{-1}.s^{-1}$)**	**K_M (mM)**	**k_{cat} (s^{-1})**	**k_{cat}/K_M ($M^{-1}.s^{-1}$)**	
*Fgr*AAO	1700 ± 150	21 ± 1.0	**12**	460 ± 91	9.7 ± 0.55	**21**	450 ± 74	36 ± 1.2	**79**	This work
*Fox*AAO	1600 ± 150	23 ± 1.2	**14**	510 ± 82	16 ± 0.72	**31**	620 ± 57	19 ± 0.35	**31**	This work
*Fgr*GalOx	102 ±6.4	1059 ±18.9	**10400 ±680**	2.48 ±0.512	22.8 ±3.57	**9.2 ±2.4**	ND	ND	**ND**	[1]
R330K[a]	895 ±85.9	208 ±10.8	**232 ±25**	1.81 ±0.111	80.4 ±5.01	**75.1 ±9.1**	ND	ND	**ND**	[1]
M_1[a]	43 ±2	1376 ±35	**32 000 ±1700**	ND	ND	**ND**	ND	ND	**ND**	[2]
M_3[b]	54 ±7	1800 ±400	**31 ±7**	ND	ND	**ND**	ND	ND	**ND**	[3]
Des 3-2[a]	ND	ND	**1100 ±300**	ND	ND	**ND**	ND	ND	**ND**	[4]
H_1[b]	ND	ND	**ND**	ND	ND	**ND**	ND	ND	**35 ± 3**	[5]
C383N[a]	390 ±38	410 ±17	**1100 ±150**	ND	ND	**ND**	ND	ND	**ND**	[6]
C383S, Y436H, V494A[a]	19 ±2	1301 ±46	**68500 ±7600**	ND	ND	**ND**	ND	ND	**ND**	[7]
V494A[a]	53 ±8	1119 ±66	**21100 ±3400**	ND	ND	**ND**	ND	ND	**ND**	[7]
*Cgr*AAO	ND	ND	**13.1 ±0.8**	ND	ND	**ND**	ND	ND	**ND**	[8]

* When comparing catalytic constants, please note that different assay temperatures, generally in the range 25 ˚C - 35 ˚C, may have been used for each enzyme, as described in the original publication cited. ND = not determined.

[a] WT *Fgr*GalOx background; [b] *Fgr*GalOx M_1 background. M_1 = S10P, M70V, P136, G195E, V494A, N353D; M_3 = W290F, R330K and Q406T, Des 3-2 = Q326E, Y329K and R330K, H_1 = R330K

Table S4. Comparison of catalytic parameters of *Fgr*AAO and *Fox*AAO with other enzymes acting on benzyl alcohol and other aryl alcohols*

Enzyme	*Benzyl alcohol*			*Cinnamyl alcohol*			References
	K_M (mM)	k_{cat} (s^{-1})	k_{cat}/K_M ($M^{-1}.s^{-1}$)	K_M (mM)	k_{cat} (s^{-1})	k_{cat}/K_M ($M^{-1}.s^{-1}$)	
*Fgr*AAO	86 ± 12	37 ± 2.8	**430**	ND	ND	3000	This work
*Fox*AAO	30 ± 19	31 ± 4.2	**1000**	ND	ND	3200	This work
*Fgr*GalOx	ND	ND	**424 ±2**	ND	ND	ND	[9]
*Cgr*AlcOx	10.69 ±0.04	94 ±1	**140000**	0.06 ±0.003	93 ±1	1600000	[10]
*Cgr*AAO	27 ±0.9	54.5 ±0.6	**2020 ± 70**	ND	ND	ND	[8]
	3-methoxybenzyl alcohol			*4-methoxybenzyl alcohol*			References
	K_M (mM)	k_{cat} (s^{-1})	k_{cat}/K_M ($M^{-1}.s^{-1}$)	K_M (mM)	k_{cat} (s^{-1})	k_{cat}/K_M ($M^{-1}.s^{-1}$)	
*Fgr*AAO	6.5 ± 1.5	52 ± 1.5	**8000**	59 ±7.2	32 ±1.8	**540**	This work
*Fox*AAO	6.7 ± 0.59	34 ± 1.7	**5100**	51 ± 12	34 ± 2.9	**290**	This work
*Fgr*GalOx	ND	ND	**4172 ±14**	ND	ND	**449 ±2**	[9]
*Cgr*AlcOx	ND	ND	**ND**	ND	ND	**ND**	[10]
*Cgr*AAO	21 ±0.8	140 ±2	**6600 ± 300**	24 ±1.3	48 ±1	**2000 ± 120**	[8]

* When comparing catalytic constants, please note that different assay temperatures, generally in the range 25 ˚C - 35 ˚C, may have been used for each enzyme, as described in the original publication cited. ND = not determined.

Table S5. Comparison of catalytic parameters of *Fgr*AAO and *Fox*AAO with other enzymes acting on HMF and its derivatives*

Enzyme	*HMF*			*DFF*			*HMFCA*			*FFCA*			References
	K_M (mM)	k_{cat} (s^{-1})	k_{cat}/K_M ($M^{-1}.s^{-1}$)	K_M (mM)	k_{cat} (s^{-1})	k_{cat}/K_M ($M^{-1}.s^{-1}$)	K_M (mM)	k_{cat} (s^{-1})	k_{cat}/K_M ($M^{-1}.s^{-1}$)	K_M (mM)	k_{cat} (s^{-1})	k_{cat}/K_M ($M^{-1}.s^{-1}$)	
*Fgr*AAO	14 ± 2.6	29 ± 1.7	**2100**	10 ± 2.9	3.6 ± 0.27	**360**	4.4 ± 0.90	3.8 ± 0.33	**860**	ND	ND	**ND**	This work
*Fox*AAO	17 ± 4.9	26 ± 2.6	**1500**	3.7 ± 1.0	1.4 ± 0.13	**380**	3.1 ± 0.31	5.2 ± 0.14	**1700**	ND	ND	**ND**	This work
*Cgr*AAO	6.5 ±0.3	126 ± 1.5	**1940 ± 90**	ND	ND	**ND**	26.9 ±3	28.3 ±1.3	**1100 ± 100**	ND	ND	**ND**	[8]
Bacterial HMFO	1.4	9.9	**7100**	1.7	1.6	**940**	73	8.5	**120**	ND	ND	**<10**	[11]
*Per*AAO	1.6 ± 0.2	0.33 ± 0.01	**220 ±42**	3.3 ± 0.2	0.52 ± 0.01	**158 ± 9.2**	ND	ND	**ND**	ND	ND	**ND**	[12]
*Mt*GLOX	20.2 ± 9.0	15.9	**982**	ND	ND	**ND**	ND	ND	**ND**	ND	ND	**ND**	[13]
*Pci*GLOX1	15.66 ± 2.35	1.59 ± 0.12	**101.66 ± 0.01**	4.38 ± 0.1	0.54 ± 0.24	**124.39 ± 0.01**	ND	ND	**ND**	0.85 ± 0.14	0.03 ± 0.01	**38.55 ± 0.01**	[14]
*Pci*GLOX2	5.87 ± 2.04	0.56 ± 0.09	**96.04 ±0.01**	0.21 ± 0.04	4.80 ± 0.24	**23400 ± 100**	ND	ND	**ND**	1.40 ± 0.39	2.02 ± 0.03	**1400 ± 10**	[14]
*Pci*GLOX3	6.35 ± 1.32	0.75 ± 0.07	**118.35 ± 0.01**	8 ±0.05	1.28 ± 0.09	**7300 ± 10**	ND	ND	**ND**	0.61 ± 0.58	0.04 ± 0.01	**72.03 ± 0.01**	[14]

* When comparing catalytic constants, please note that different assay temperatures, generally in the range 25 ˚C - 35 ˚C, may have been used for each enzyme, as described in the original publication cited. ND = not determined.

Table S6. Assignments of ^{1}H chemical shifts (ppm) of substrates and products of *Fgr*AAO oxidation of 1-O-Methyl D-fructose as determined by 1D and 2D NMR experiments recorded at 400MHz in D_2O. The peaks are referenced to internal acetone (2.22 ppm).

Compound	H-1	H-3	H-4	H-5	H-6	-OMe
**3**	3.77 (S)	3.93 (D)	3.87 - 3.84 (M)	3.82 (M)	3.76 (DD)	3.28 (S)
**8**	5.17 (S)	4.09 (D)	3.87 - 3.85 (M)	3.97 (M)	3.77 (S)	3.37 (S)
**2**	3.68 (DD)	4.17 (D)	3.87 -3.83 (M)	4.05 (T)	3.72 (DD)	3.32 (S)
**7**	3.70 (DD)	4.17 (S)	3.64 (S)	4.17 (S)	4.95 (D)	3.30 (S)
**1**	3.72 (DD)	4.10 (M)	3.96 (M)	3.96 (M)	3.75 (DD)	3.32 (S)

Table S7. Assignments of ^{13}C chemical shifts (ppm) of compounds substrates and products of *Fgr*AAO oxidation of 1-O-Methyl D-fructose as determined by 1D and 2D NMR experiments recorded at 400MHz in D_2O. The peaks are referenced to internal acetone (30.89 ppm).

Compound	C-1	C-2	C-3	C-4	C-5	C-6	-OMe
**3**	61.4	101.0	68.9	70.2	69.6	64.4	49.1
**8**	90.0	99.7	68.5	70.3	69.4	64.7	49.8
**2**	60.3	104.4	77.4	81.6	75.6	63.3	49.6
**7**	59.7	104.7	77.7	83.2	76.9	91.6	49.3
**1**	58.3	108.9	80.7	83.9	78.0	61.9	48.8

Table S8. Assignments of ^{1}H and ^{13}C chemical shifts (ppm) of *Fgr*AAO oxidation of raffinose as determined by 1D and 2D NMR experiments recorded at 400MHz in D_2O. The peaks are referenced to internal acetone (2.22 ppm).

Ring System ^{1}H	δ (ppm)	Multiplicity	Coupling Constant (J) (Hz)	Ring System ^{13}C	δ (ppm)
**Galactose (Product)**				**Galactose (Product)**	
H_1	3.55 or 3.64	M	n.a.	C_1	n.a.
H_2	3.55 or 3.64	M	n.a.	C_2	n.a.
H_3	4.54	DD	2.98; 8.02	C_3	66.41
H_4	5.27	D	2.54	C_4	99.96
H_5	3.89	M	n.a.	C_5	70.54
H_6	6.19	D	3.00	C_6	125.11
**Galactose (Substrate)**				**Galactose (Substrate)**	
H_1	4.99	D	3.63	C_1	99.09
H_2	3.83	M	n.a.	C_2	69.10
H_3	3.89	M	n.a.	C_3	70.07
H_4	4.00	D	n.a.	C_4	69.82
H_5	3.95	T	6.28	C_5	71.64
H_6/H'_6	3.76	M	n.a.	C_6	61.72
**Glucose (Product)**				**Glucose**	
H_1	5.39	D	3.91	C_1	92.70
H_2	3.55	M	n.a.	C_2	n.a.
H_3	3.76	M	n.a.	C_3	73.22
H_4	3.55	M	n.a.	C_4	69.98
H_5	4.00	D	n.a.	C_5	71.98
H_6/H'_6	4.05; 3.89	M; M	n.a.	C_6	67.94
**Glucose (Substrate)**				**Glucose (Substrate)**	
H_1	5.42	D	3.83	C_1	92.73
H_2	3.55	M	n.a.	C_2	71.58
H_3	3.76	M	n.a.	C_3	73.27
H_4	3.55	M	n.a.	C_4	70.02
H_5	4.05	M	n.a.	C_5	72.02
H_6/H'_6	4.05; 3.70	M; M	n.a.	C_6	66.50
**Fructose (Product)**				**Fructose (Product)**	
H_1/H'_1	3.70	M	n.a.	C_1	70.21
H_3	4.14	M	n.a.	C_2	104.39
H_4	3.55	M	n.a.	C_3	69.34
H_5	3.64	M	n.a.	C_4	74.67
H_6	5.12	D	7.36	C_5	73.40
				C_6	89.09
**Fructose (Substrate)**				**Fructose (Substrate)**	
H_1/H'_1	3.64	M	n.a.	C_1	62.01
H_3	4.21	M	n.a.	C_2	104.43
H_4	4.05	M	n.a.	C_3	76.95
H_5	3.89	M	n.a.	C_4	74.60
H_6/H'_6	3.83; 3.76	M; M	n.a.	C_5	81.97
				C_6	63.09

Supporting Figures

```
FgrAAO       --GVGKWGPTLDFPVIPVAGAVEPVSGKVVIWSAYRYDAFQGTNPRGGFTLTSIWDPKTN
FoxAAO       AKGLGKWGPTLDFPVIPVAGAVEPVSGKVVIWSAYRYDAFQGTTPRGGFTLTSIWDPKTN
FgrGalOx     --GLGRWGPTIDLPIVPAAAAIEPTSGRVLMWSSYRNDAFGGSP--GGITLTSSWDPSTG
CgrAlcOx     --NVGKWGPMVKFPVVPVAVALVPETGNLLVWSSG--WPNRWTTAGNGKTYTSLYNVNTG

FgrAAO       VISNRNVTNNKHDMFCPGISMDGEGQIVVTGGNDAKKTTILNP---NGE-WVPGPDMQIA
FoxAAO       VISNRNVSNNHHDMFCPGISMDGEGQIVVTGGNDAKKTTILMP---DGN-WVPGPDMQIA
FgrGalOx     IVSDRTVTVTKHDMFCPGISMDGNGQIVVTGGNDAKKTSLYDS---SSDSWIPGPDMQVA
CgrAlcOx     NISDAIVQNTQHDMFCPGTSLDADGRIIVTGGSSAAKTSVLDFKKGESSPWTPLSNMQIS

FgrAAO       RGYQSSATTSDGRVFTIGGSWSGPRGGKNGEIYDPKARTWTSLPKCLVGPMLTKDKEGVY
FoxAAO       RGYQSSATCSDGRVFTIGGSWSGPRGGKNGEIYDPKAKTWTSLPKCLVGPMLTKDKEGVY
FgrGalOx     RGYQSSATMSDGRVFTIGGSWSGGVFEKNGEVYSPSSKTWTSLPNAKVNPMLTADKQGLY
CgrAlcOx     RGYQSSCTTSEGKIFVIGGSFSG-AGTRNGEVYDPKANTWTKLAGCPVKPLVMQRG---M

FgrAAO       KADNHAWLFGWKKGSVFQAGPSTAMNWYYTTRGTQGDTKAAGTRRKNGRVDPDSMNGNCV
FoxAAO       KADNHAWLFGWKKNSVFQAGPSTAMNWYYTTRGTQGDTKAAGTRRKNGRIDPDSMNGNVA
FgrGalOx     RSDNHAWLFGWKKGSVFQAGPSTAMNWYYTSG--SGDVKSAGKRQSNRGVAPDAMCGNAV
CgrAlcOx     FPDSHAWLWSWKNGSVLQAGPSKKMNWYDTKG------TGSNTPAGLRGTDEDSMCGVSV

FgrAAO       MYDALDGKILTYGGATSYQQAPATANAHVLAIAEPGAIAQTYLVGNNGAGNYARVFHTSV
FoxAAO       MFDALNGKILTFGGATSYQQAPATANAHVLTIDEPGAIAQTALVGNNGAGIHARVFATSV
FgrGalOx     MYDAVKGKILTFGGSPDYQDSDATTNAHIITLGEPGTSPNTVFASNG--LYFARTFHTSV
CgrAlcOx     MYDAVAGKIFTYGGGKGYTGYDSTSNAHILTLGEPGQAVQVQKLANG---KYNRGFANAV

FgrAAO       VLPDGNVFITGGQSYSNPFTDTNAQLTPEMYIPTTHEFKTQQPNTIPRTYHSMSLLLPDA
FoxAAO       ILPDGNVFITGGQSYSDPFTDTNAQLEPEMFISSSNTFTKQQTNTIPRTYHSMSLLLPDA
FgrGalOx     VLPDGSTFITGGQRRGIPFEDSTPVFTPEIYVPEQDTFYKQNPNSIVRVYHSISLLLPDG
CgrAlcOx     VMPDGKIWVVGGMQKMWLFSDTTPQLTPELFDPATGSFTPTTPHTVPRNYHSTALLMADA

FgrAAO       TVFNGGGGLCGS-CSSNHFDAQIYTPQYLLDGN-GNFATRPKITAVSATTAKIGSTITVT
FoxAAO       TVFNGGGGLCGG-CKTNHFDAQIFTPQYLLDGN-GNLATRPKITAVSATTAKVGSTITVT
FgrGalOx     RVFNGGGGLCGD-CTTNHFDAQIFTPNYLYNSN-GNLATRPKITRTSTQSVKVGGRITIS
CgrAlcOx     TIWSGGGGLCGANCKENHFDGQFWSPPYLFEADGVTPAKRPVIQSLSDTAVRAGAPITIT

FgrAAO       ANSA-IKSASLIRYGTATHTVNTDQRRIPLALTGAG-TNKYSFKIPNDSGIALPGYWMLF
FoxAAO       ANSA-IKSASLIRYGTATHVVNTDQRRIPLALTGAG-TNKYSFKIPNDSGIALPGYWMLF
FgrGalOx     TDSS-ISKASLIRYGTATHTVNTDQRRIPLTLTNNG-GNSYSFQVPSDSGVALPGYWMLF
CgrAlcOx     MQDAGAYTFSMIRVSATTHTVNTDQRRIPLDGQDGGDGKSFTVNVPNDYGVAIPGYYMLF

FgrAAO       VLNNAGVPSVASTIKVTV
FoxAAO       VLNNAGVPSVASTIKVTI
FgrGalOx     VMNSAGVPSVASTIRVTQ
CgrAlcOx     AMNEAGVPCVAQFFKVTL
```

Figure S1. Sequence alignment of catalytic domains of *Fusarium graminearum* AAO (*Fgr*AAO), *Fusarium oxysporum* AAO (*Fox*AAO), *Fusarium graminearum* GalOx (*Fgr*GalOx) and *Colletotrichum graminicola* AlcOx (*Cgr*AlcOx). Predictive signal peptides and N-terminal modules have been removed. Conserved active-site catalytic residues and residues involved in substate recognition and stability are highlighted in green and yellow, respectively.

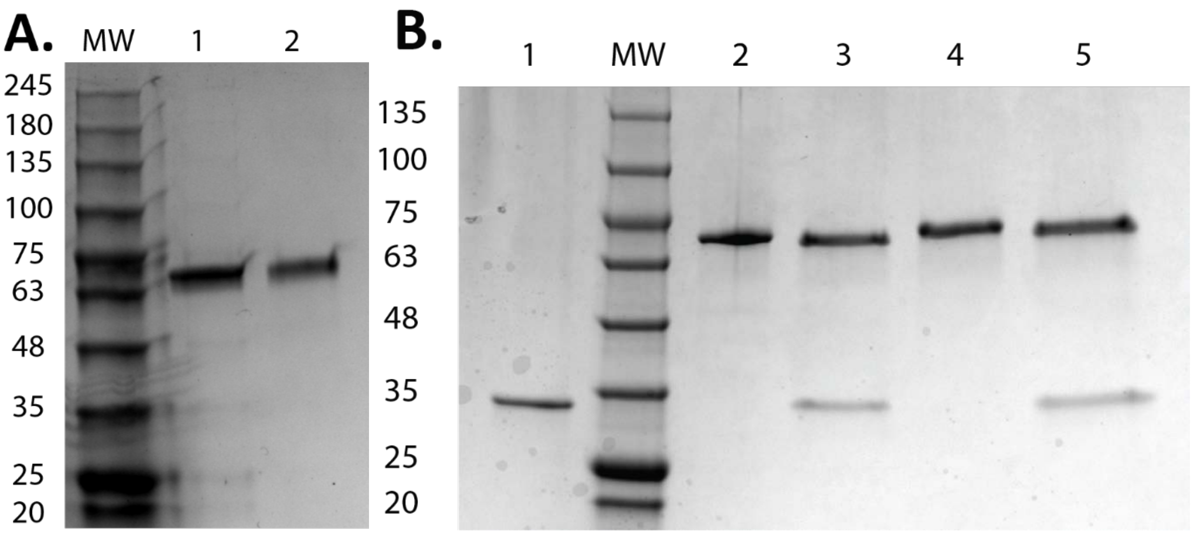

Figure S2. SDS-PAGE of *Fgr*AAO and *Fox*AAO and N-deglycosylation study. A. Aliquot of purified *Fgr*AAO (4 µg) (lane 1) and purified *Fox*AAO (4 µg) (lane 2). B. *Fgr*AAO and *Fox*AAO (3 µg 1 to 6) were N-deglycosylated under denaturing conditions with PNGaseF. 1: PNGaseF, 2: native *Fgr*AAO (3 µg), 3: *Fgr*AAO (3 µg) + pNGaseF, 4: native *Fox*AAO (3 µg), 5: *Fox*AAO (3 µg) + pNGaseF. All gels were stained by Coomassie blue. MW = molecular weight markers, as indicated.

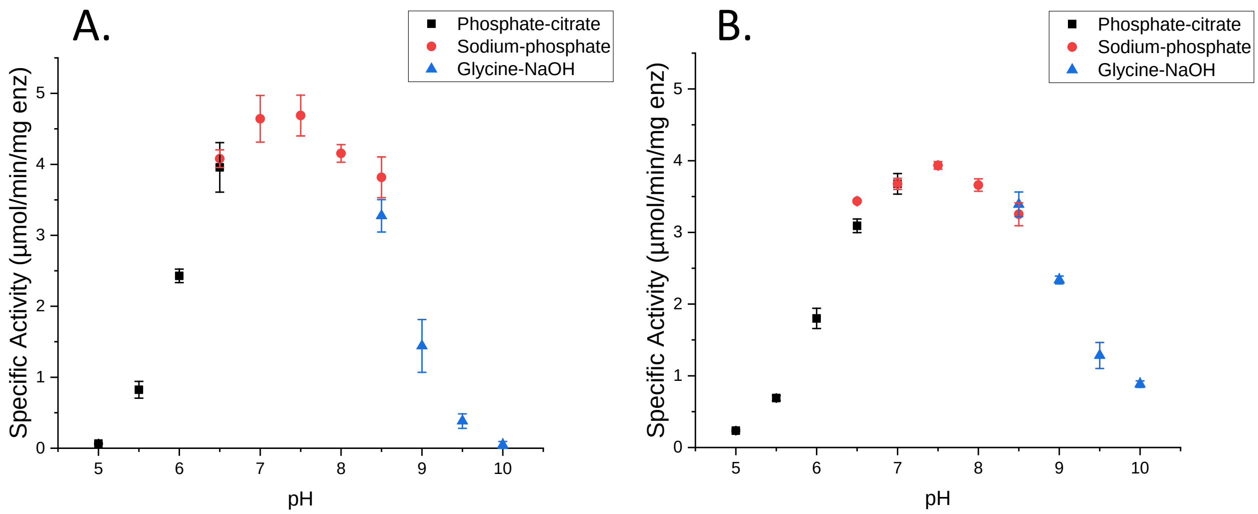

Figure S3. pH-rate profiles. A. *Fgr*AAO; B. *Fox*AAO. pH-rate profiles were determined using the coupled HRP-ABTS assay with 1M galactose as the substrate at each pH value. Black squares for phosphate citrate buffer, red circles for sodium phosphate buffer and blue triangles for glycine NaOH buffer. Error bars represent standard deviations over three replicates.

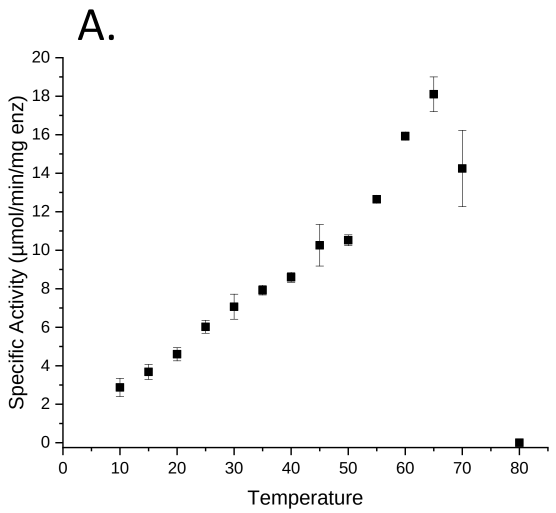

Figure S4. Temperature-rate profiles. A. *Fgr*AAO; B. *Fox*AAO. Temperature-rate profiles were determined using the coupled HRP-ABTS assay. 50 mM sodium phosphate, pH 7.5 and 1M galactose were incubated at each temperature for at least 10 min prior to the addition of HRP, ABTS and purified enzyme. Error bars represent standard deviations over three replicates.

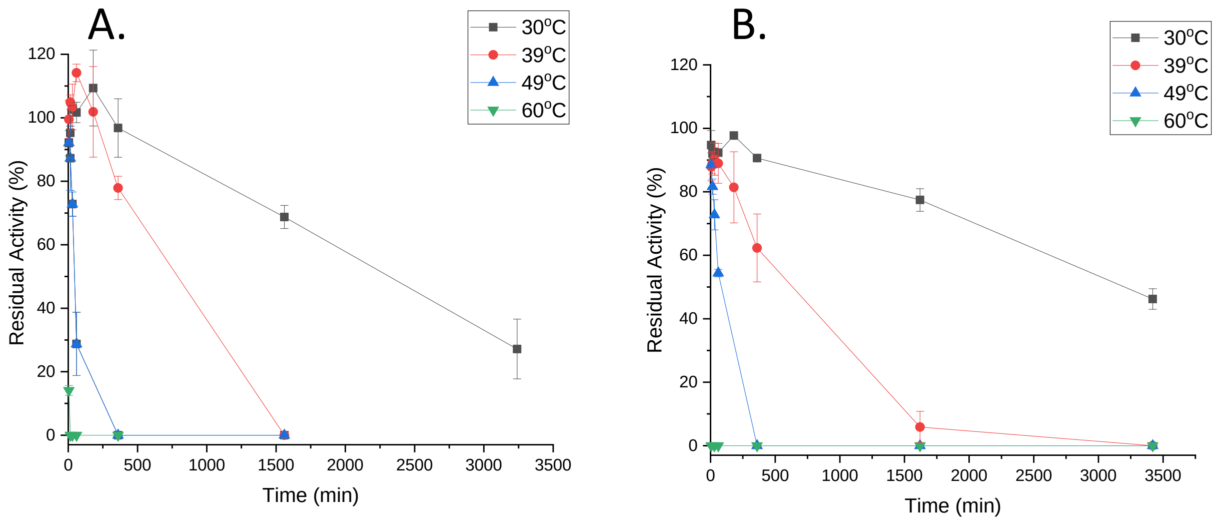

Figure S5. Temperature stability. A. *Fgr*AAO; B. *Fox*AAO. Activity values were determined using the coupled HRP-ABTS assay with 1 M galactose as the substrate. The enzyme was pre-incubated at each temperature, maintained by a gradient thermocycler: 30°C (black square), 39°C (red circle), 49°C (blue triangle), 60°C (green triangle). The reactions were performed at 30°C. Error bars represent standard deviations over duplicate measurements.

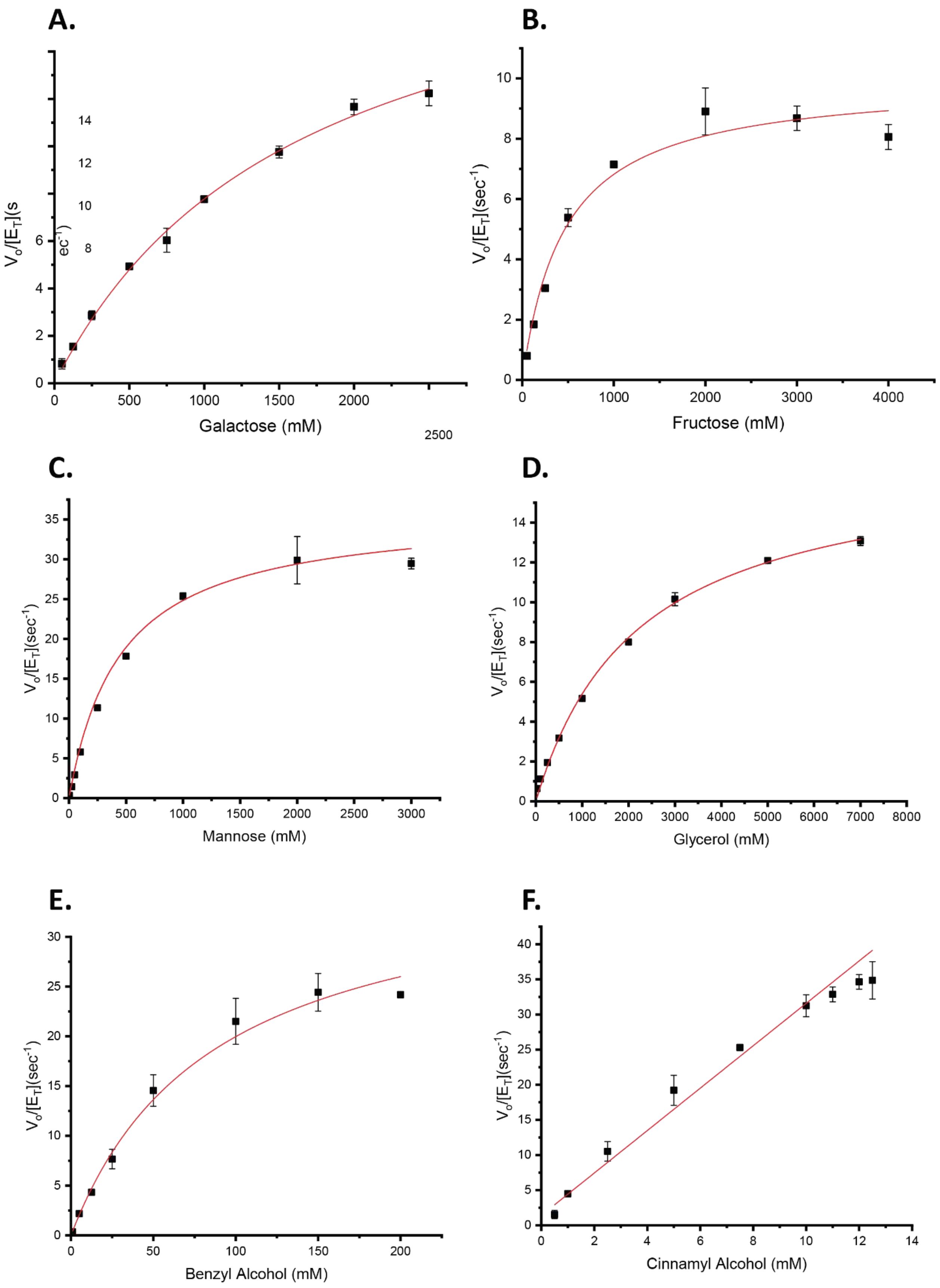

A.
Vo/[ET](s
ec-1)
0
2
4
6
8
10
12
14
0
500
1000
1500
2000
2500
Galactose (mM)
B.
Vo/[ET](sec-1)
0
2
4
6
8
10
0
1000
2000
3000
4000
Fructose (mM)
C.
Vo/[ET](sec-1)
0
5
10
15
20
25
30
35
0
500
1000
1500
2000
2500
3000
Mannose (mM)
D.
Vo/[ET](sec-1)
0
2
4
6
8
10
12
14
0
1000
2000
3000
4000
5000
6000
7000
8000
Glycerol (mM)
E.
Vo/[ET](sec-1)
0
5
10
15
20
25
30
0
50
100
150
200
Benzyl Alcohol (mM)
F.
Vo/[ET](sec-1)
0
5
10
15
20
25
30
35
40
0
2
4
6
8
10
12
14
Cinnamyl Alcohol (mM)

G.

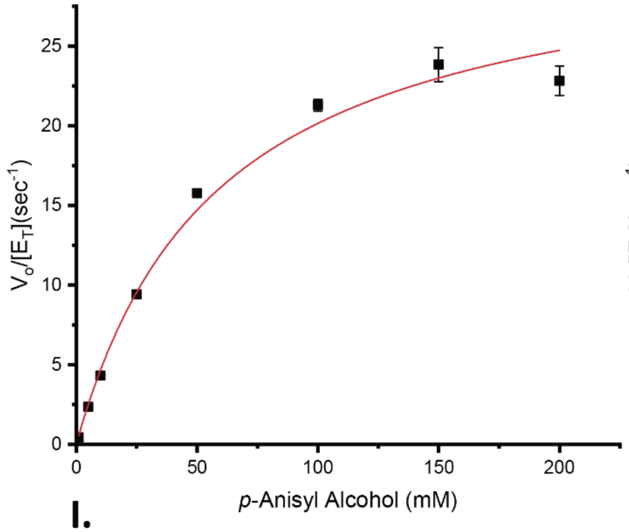

$V_o/[E_T](sec^{-1})$
0
5
10
15
20
25
0
50
100
150
200
p-Anisyl Alcohol (mM)

H.

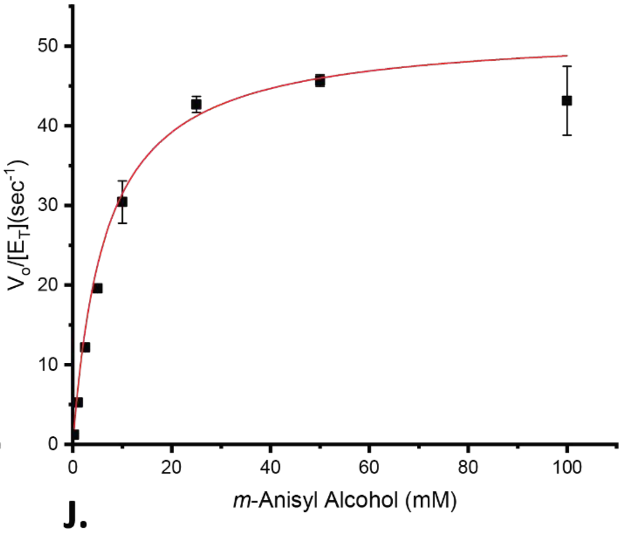

$V_o/[E_T](sec^{-1})$
0
10
20
30
40
50
0
20
40
60
80
100
m-Anisyl Alcohol (mM)

I.

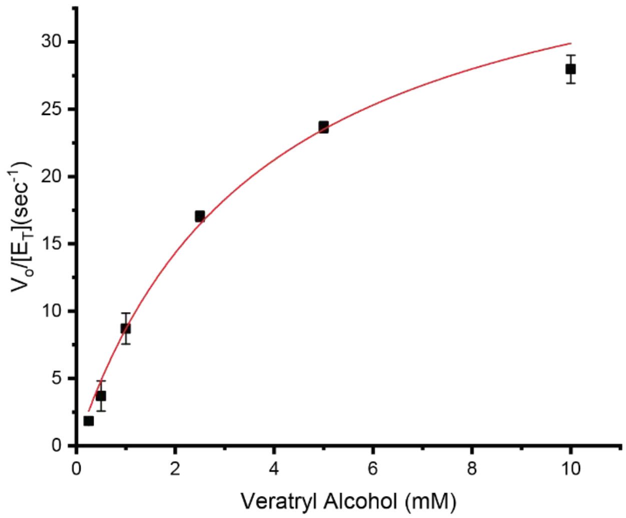

$V_o/[E_T](sec^{-1})$
0
5
10
15
20
25
30
0
2
4
6
8
10
Veratryl Alcohol (mM)

J.

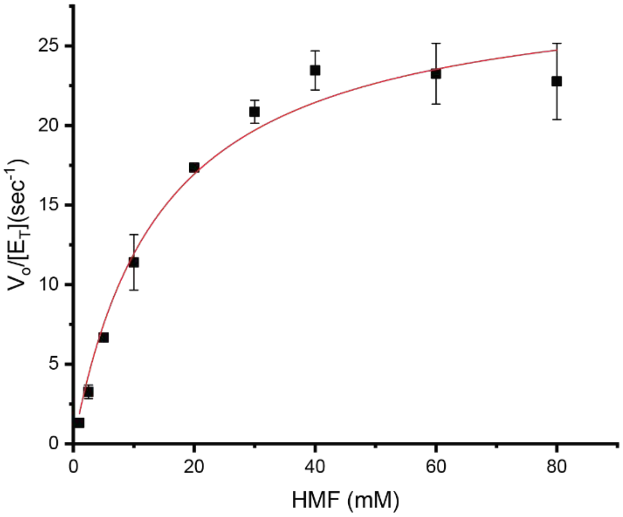

$V_o/[E_T](sec^{-1})$
0
5
10
15
20
25
0
20
40
60
80
HMF (mM)

K.

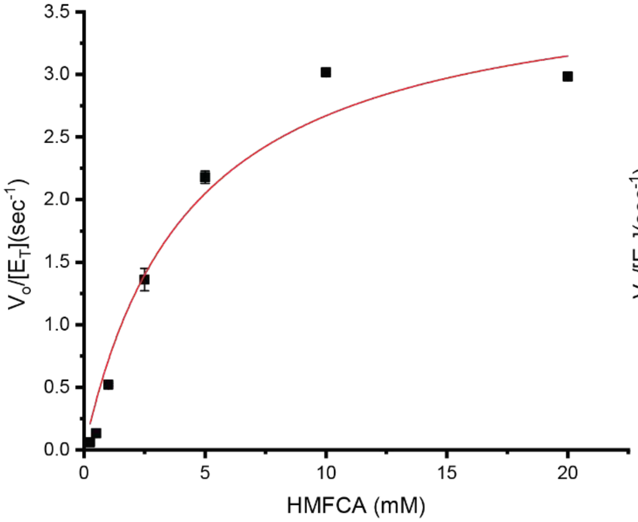

$V_o/[E_T](sec^{-1})$
0.0
0.5
1.0
1.5
2.0
2.5
3.0
3.5
0
5
10
15
20
HMFCA (mM)

L.

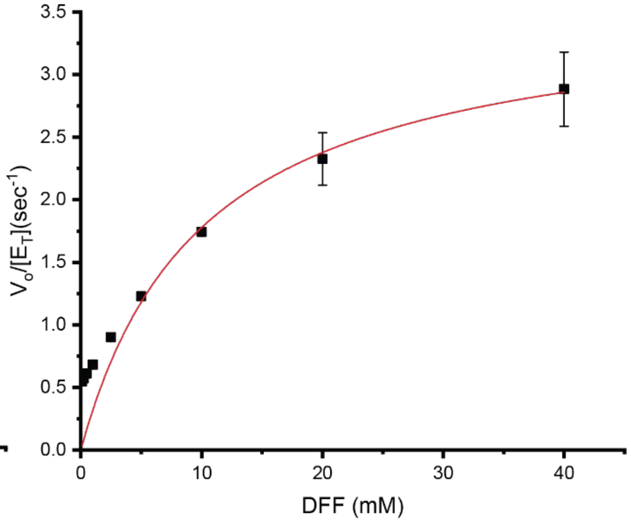

$V_o/[E_T](sec^{-1})$
0.0
0.5
1.0
1.5
2.0
2.5
3.0
3.5
0
10
20
30
40
DFF (mM)

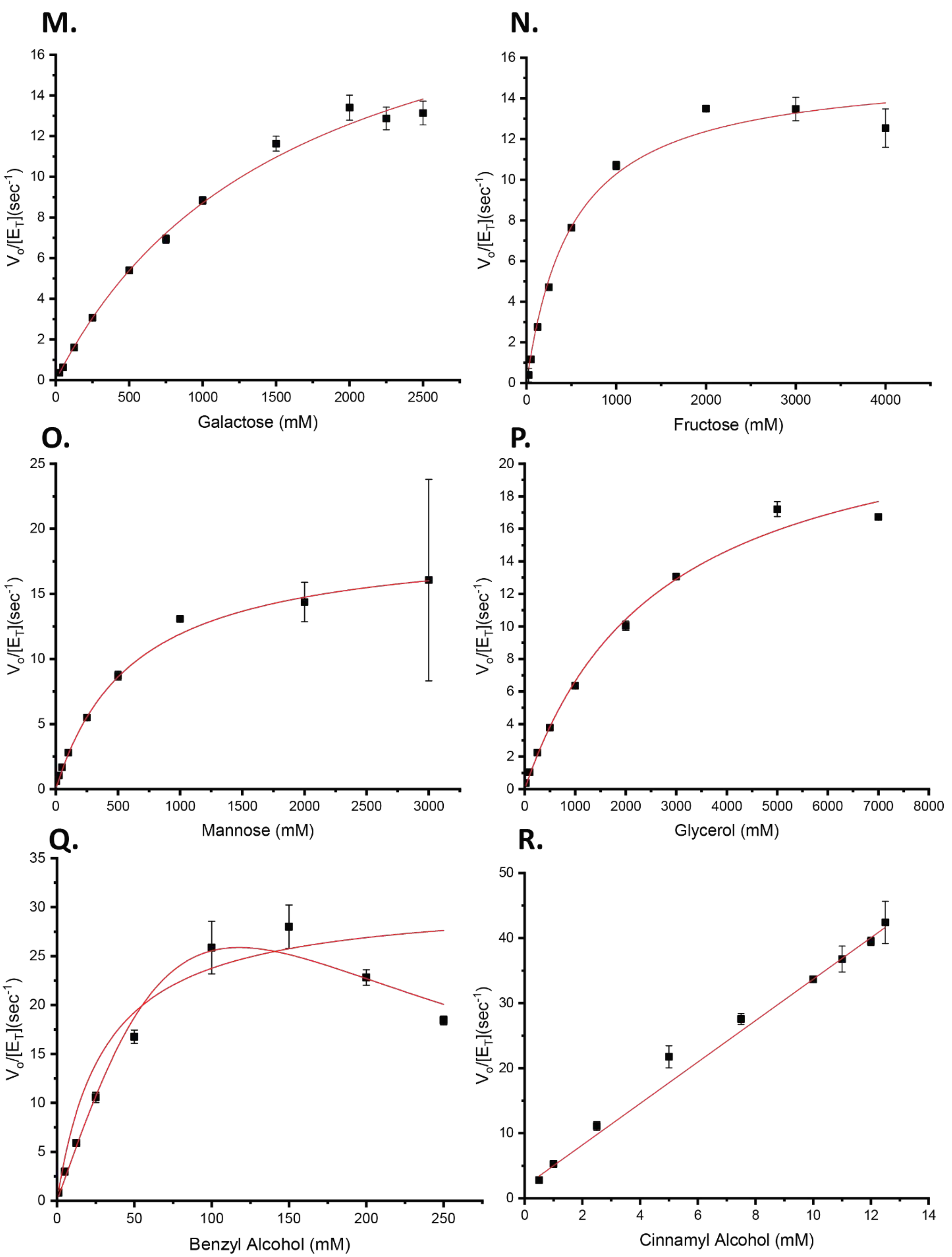
M.
16
14
12
10
8
6
4
2
0
0
500
1000
1500
2000
2500
Galactose (mM)
V_o/[E_T](sec^-1)
N.
16
14
12
10
8
6
4
2
0
0
1000
2000
3000
4000
Fructose (mM)
V_o/[E_T](sec^-1)
O.
25
20
15
10
5
0
0
500
1000
1500
2000
2500
3000
Mannose (mM)
V_o/[E_T](sec^-1)
P.
20
18
16
14
12
10
8
6
4
2
0
0
1000
2000
3000
4000
5000
6000
7000
8000
Glycerol (mM)
V_o/[E_T](sec^-1)
Q.
35
30
25
20
15
10
5
0
0
50
100
150
200
250
Benzyl Alcohol (mM)
V_o/[E_T](sec^-1)
R.
50
40
30
20
10
0
0
2
4
6
8
10
12
14
Cinnamyl Alcohol (mM)
V_o/[E_T](sec^-1)

S.

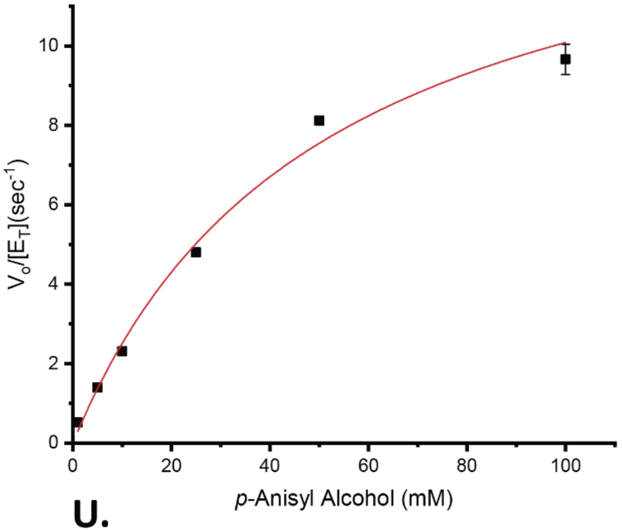
$V_o/[E_T](sec^{-1})$
p-Anisyl Alcohol (mM)

T.

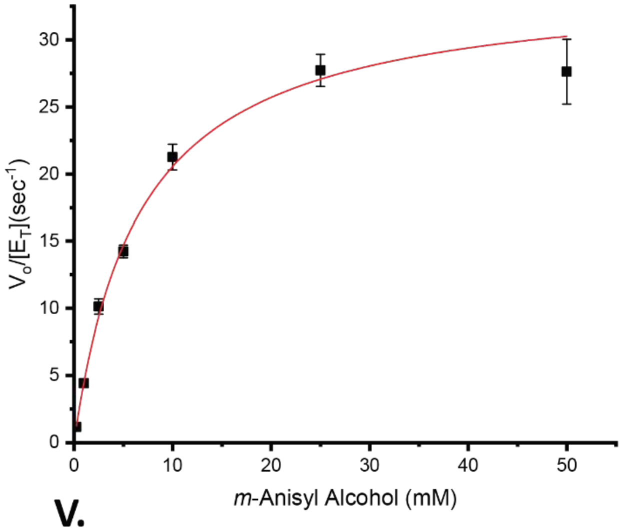
$V_o/[E_T](sec^{-1})$
m-Anisyl Alcohol (mM)

U.

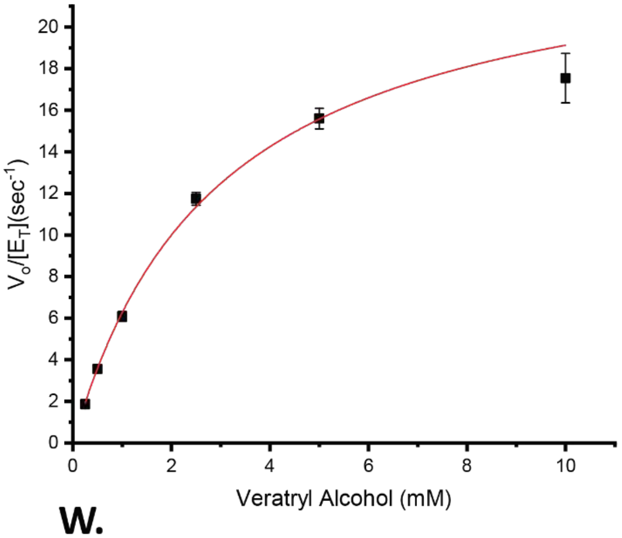
$V_o/[E_T](sec^{-1})$
Veratryl Alcohol (mM)

V.

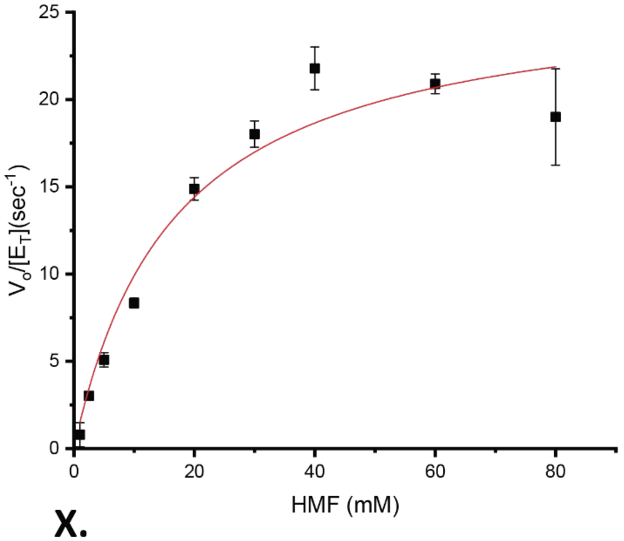
$V_o/[E_T](sec^{-1})$
HMF (mM)

W.

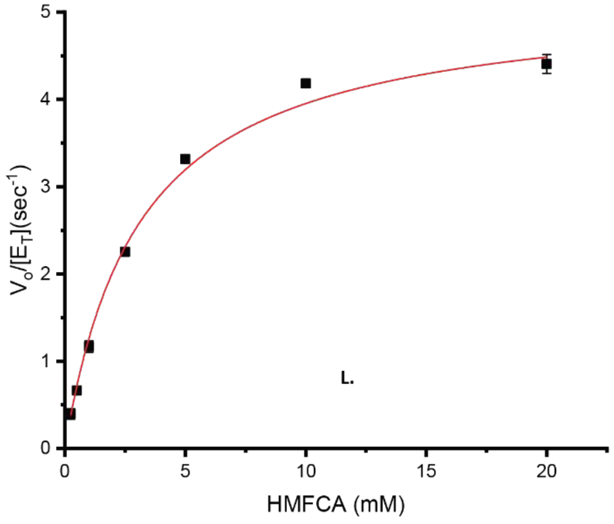
$V_o/[E_T](sec^{-1})$
L.
HMFCA (mM)

X.

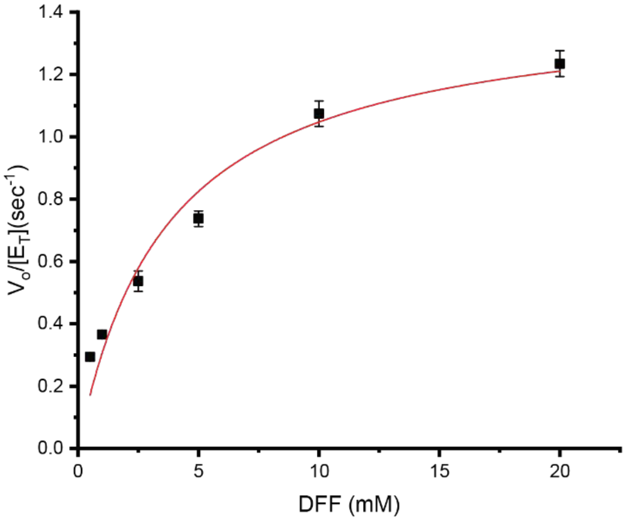
$V_o/[E_T](sec^{-1})$
DFF (mM)

Figure S6. Initial-rate kinetics. Panels A-L: *Fgr*AAO, panels M-X: *Fox*AAO. Individual substrates are indicated in the x-axis labels. Values were measured in triplicate at each substrate concentration. Error bars represent the standard deviation from the mean values. The individual k_{cat} and K_M values were determined by performing a non-linear fitting analysis of the standard Michaelis-Menten equation to the data using OriginLab 9.55. For cinnamyl alcohol, which did not display saturation kinetics for either enzyme due to solubility limitations, k_{cat}/K_M values were obtained from the slope of linear fittings. Benzyl alcohol with *Fox*AAO (panel Q) displayed apparent substrate inhibition. The data was fit both with the classical Michaelis-Menten equation and the substrate inhibition variant, $V_o/[E]_T = k_{cat}[S]/(K_M + [S] + [S]_2/K_{is})$. Due to high errors on the individual constants k_{cat}, K_M, and K_{is} in the latter case, k_{cat} and K_M values from the former are reported in Table 1.

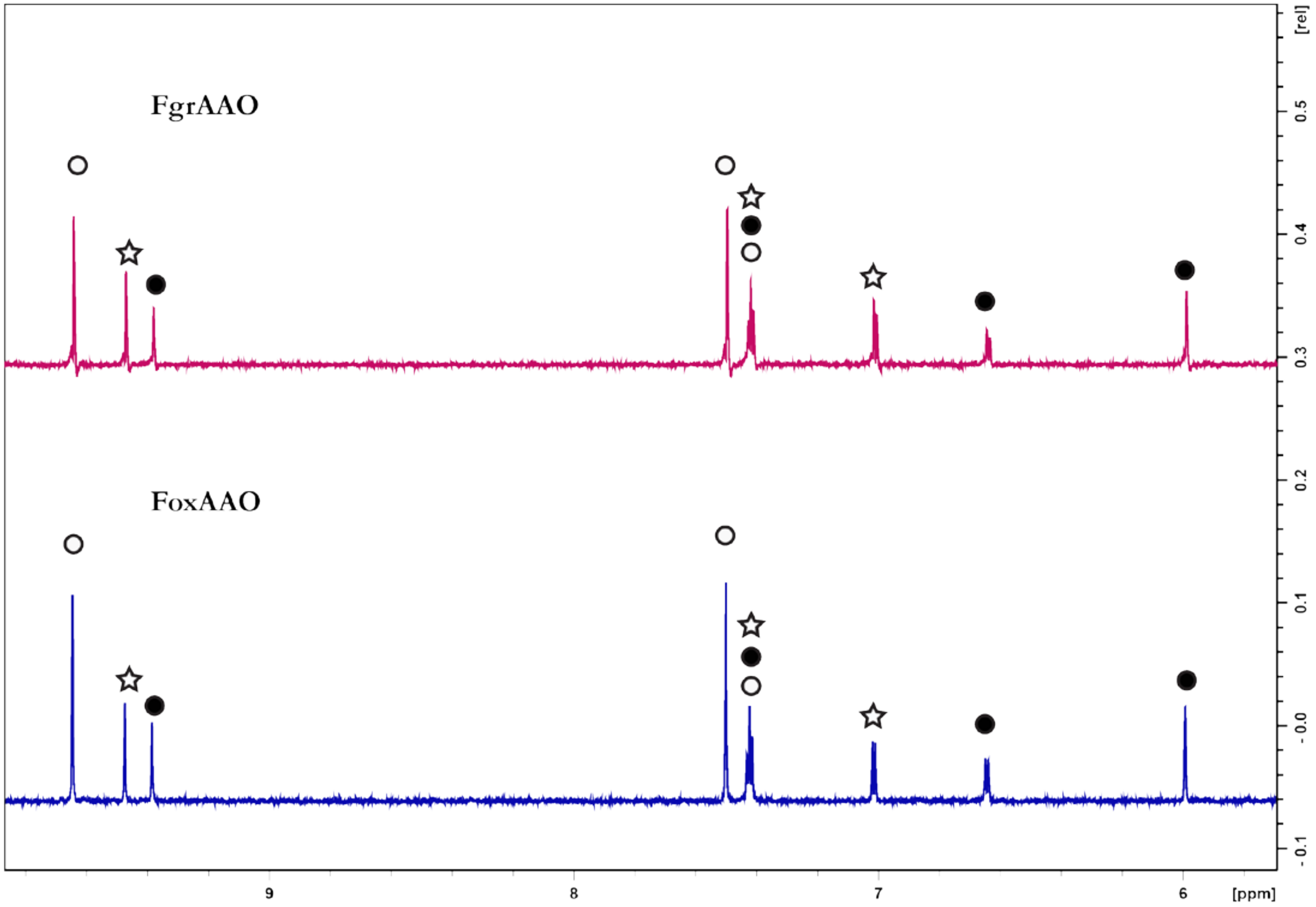

Figure S7. ^{1}H-NMR analysis of the oxidation of HMF by *Fgr*AAO and *Fox*AAO. A. *Fgr*AAO; B. *Fox*AAO. ^{1}H NMR spectra (400 MHz, 1:9 D_2O:phosphate buffer, 50 mM, pH 7.5) of reaction product profiles after 16.5 h incubation with *Fgr*AAO (top, pink) and *Fox*AAO (bottom, blue) in the presence of catalase and HRP for HMF at 10 mM showing full conversion to DFF (○) and the hydrated form, DFFhyd(●), as well as FFCA (☆). See Figure 4 for full chemical structures.

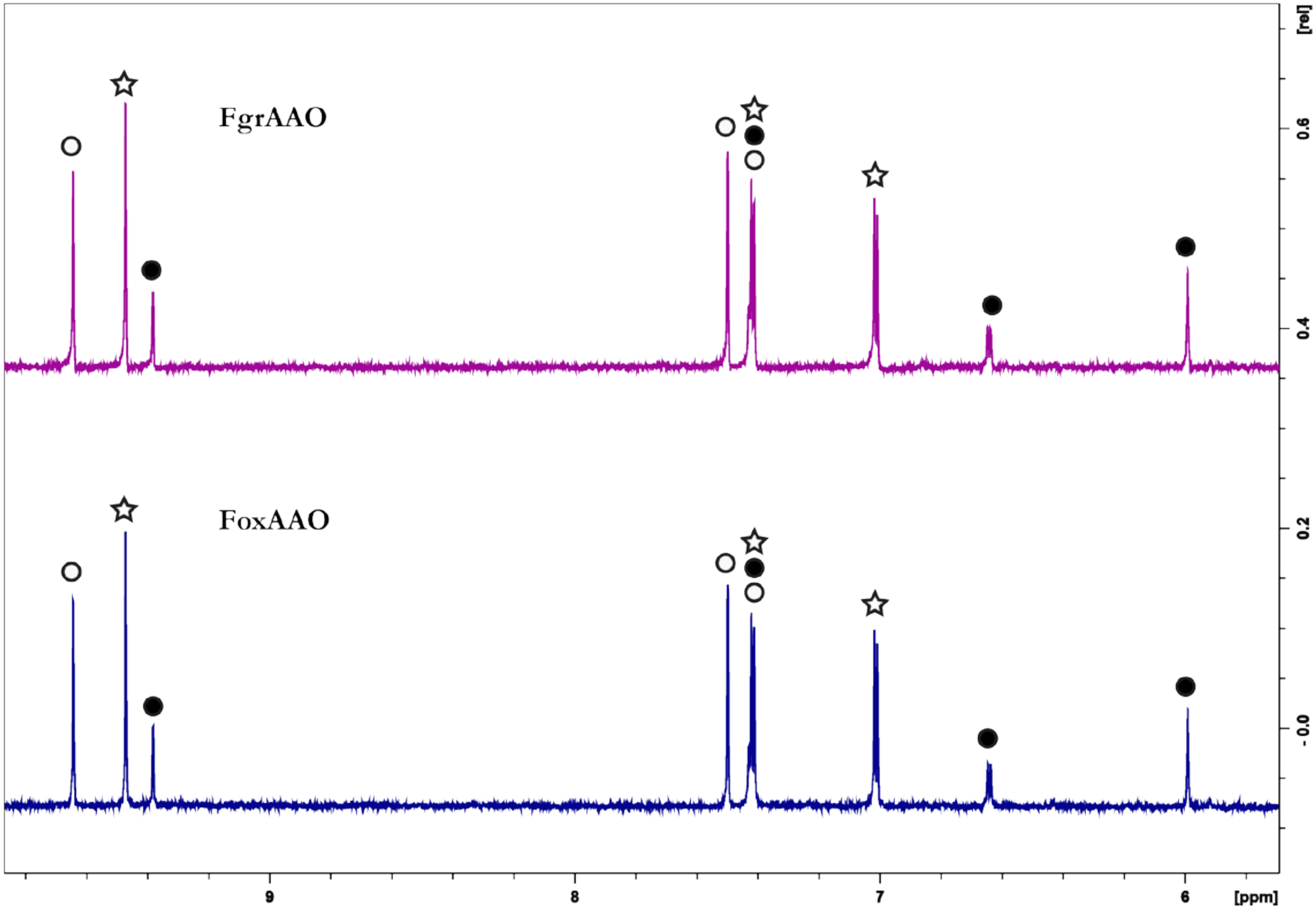

Figure S8. ^{1}H-NMR analysis of the oxidation of DFF by *Fgr*AAO and *Fox*AAO. A. *Fgr*AAO; B. *Fox*AAO. ^{1}H NMR spectra (400 MHz, 1:9 D_2O:phosphate buffer, 50 mM, pH 7.5) of reaction product profiles after 16.5 h incubation with *Fgr*AAO (top, pink) and *Fox*AAO (bottom, blue) in the presence of catalase and HRP for DFF (○ and ●) at 10 mM showing partial conversion to FFCA (☆). See Figure 4 for full chemical structures.

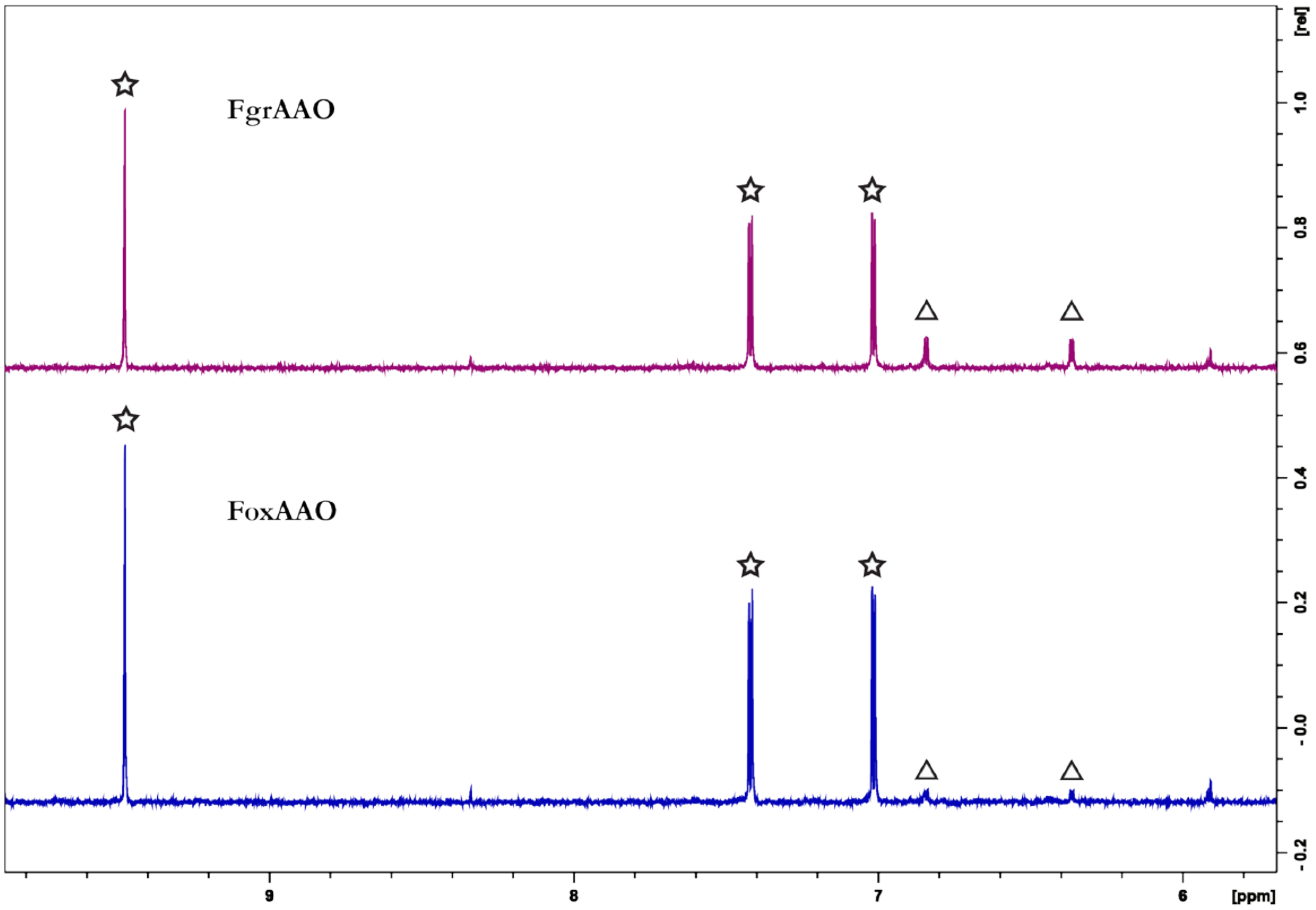

Figure S9. ^{1}H-NMR analysis of the oxidation of HMFCA by *Fgr*AAO and *Fox*AAO. A. *Fgr*AAO; B. *Fox*AAO. ^{1}H NMR spectra (400 MHz, 1:9 D2O:phosphate buffer, 50 mM, pH 7.5) of reaction product profiles after 16.5 h incubation with *Fgr*AAO (top, pink) and *Fox*AAO (bottom, blue) in the presence of catalase and HRP for HMFCA (△) at 10 mM showing almost full conversion to FFCA (☆). See Figure 4 for full chemical structures.

A.

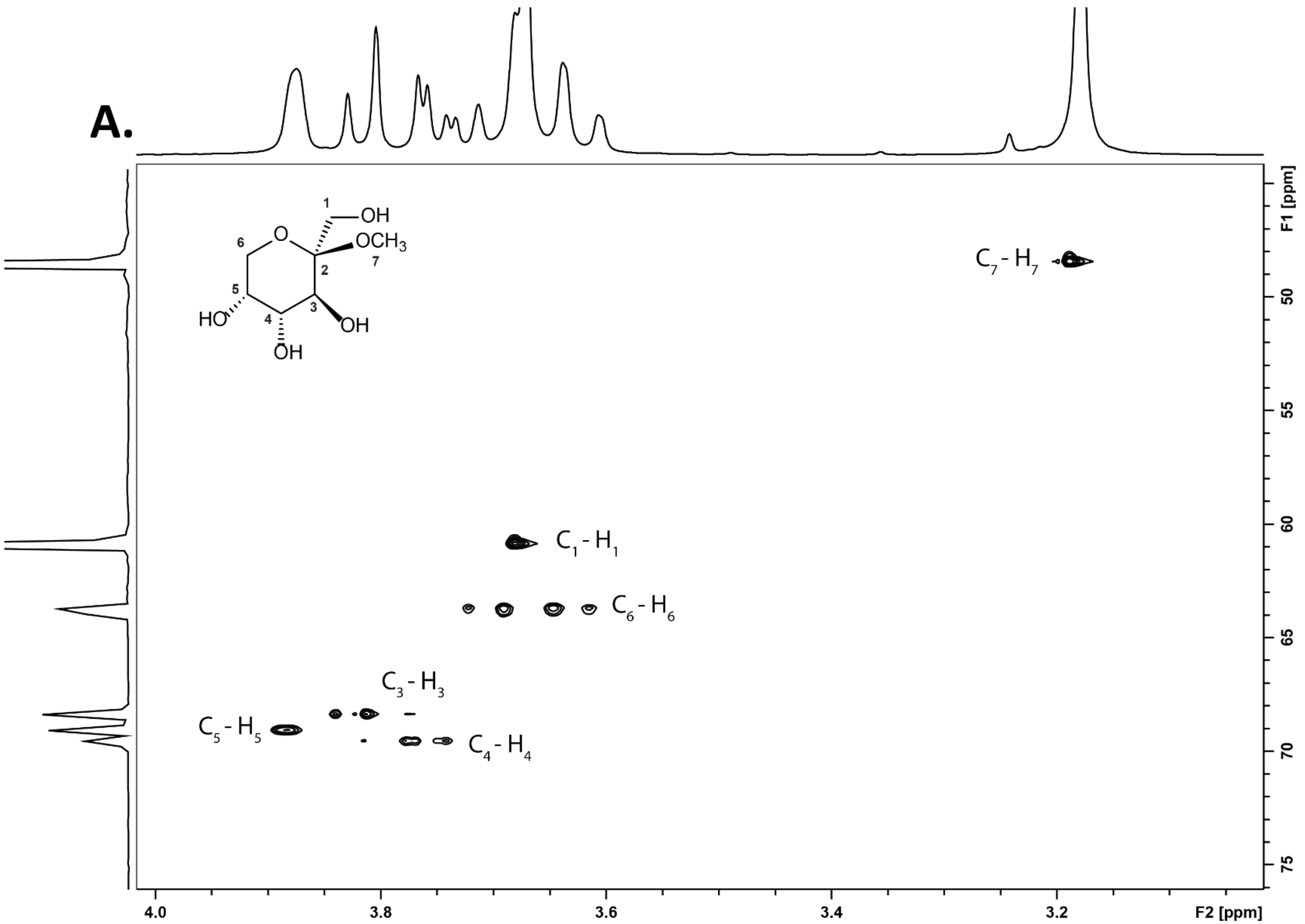

1
OH
O
OCH3
6
7
2
5
3
4
HO
OH
OH
C7 - H7
C1 - H1
C6 - H6
C3 - H3
C5 - H5
C4 - H4
F1 [ppm]
50
55
60
65
70
75
4.0
3.8
3.6
3.4
3.2
F2 [ppm]

B.

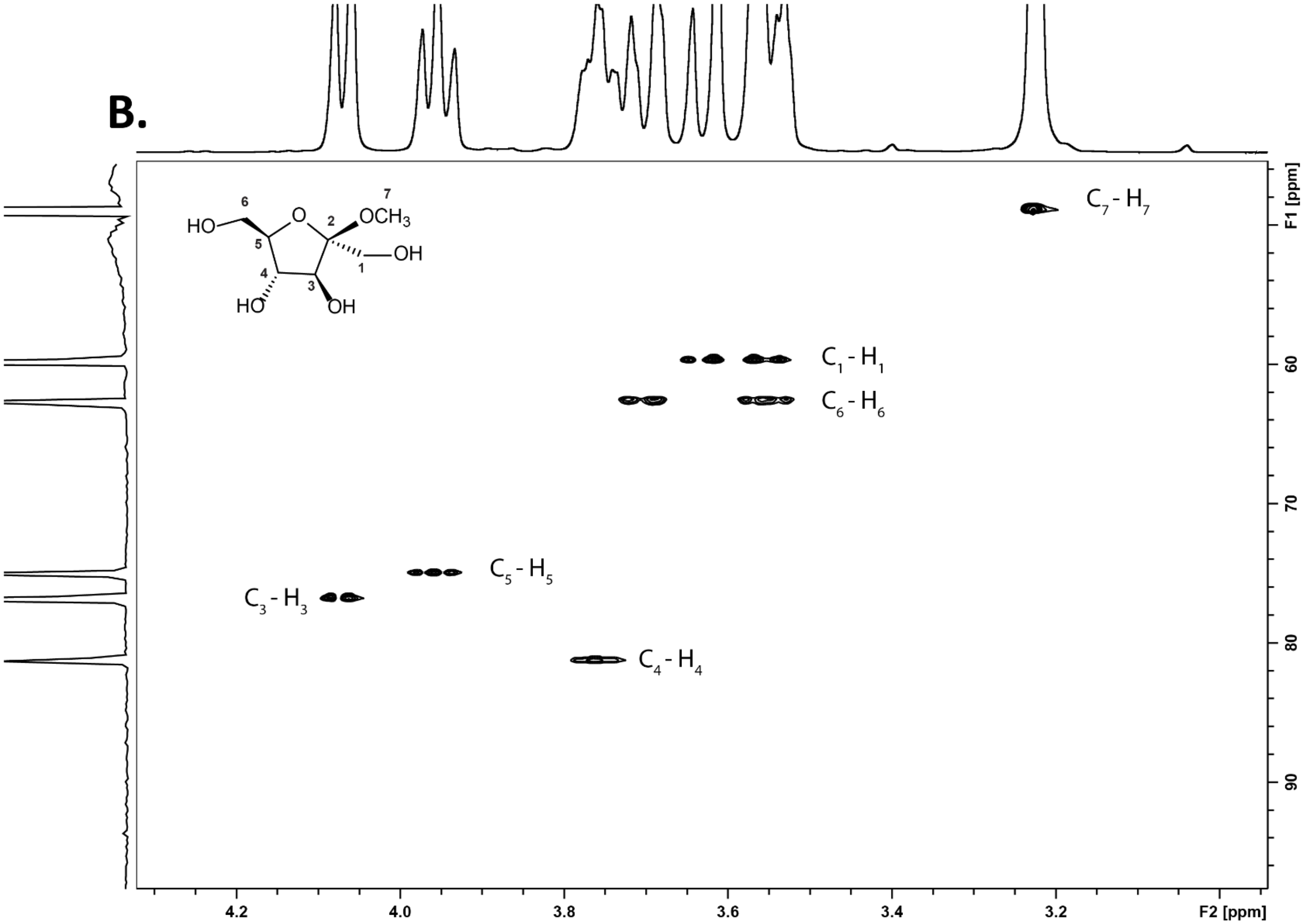
6
7
HO
O
2
OCH3
5
1
OH
4
3
HO
OH
C7 - H7
C1 - H1
C6 - H6
C5 - H5
C3 - H3
C4 - H4
F1 [ppm]
60
70
80
90
4.2
4.0
3.8
3.6
3.4
3.2
F2 [ppm]

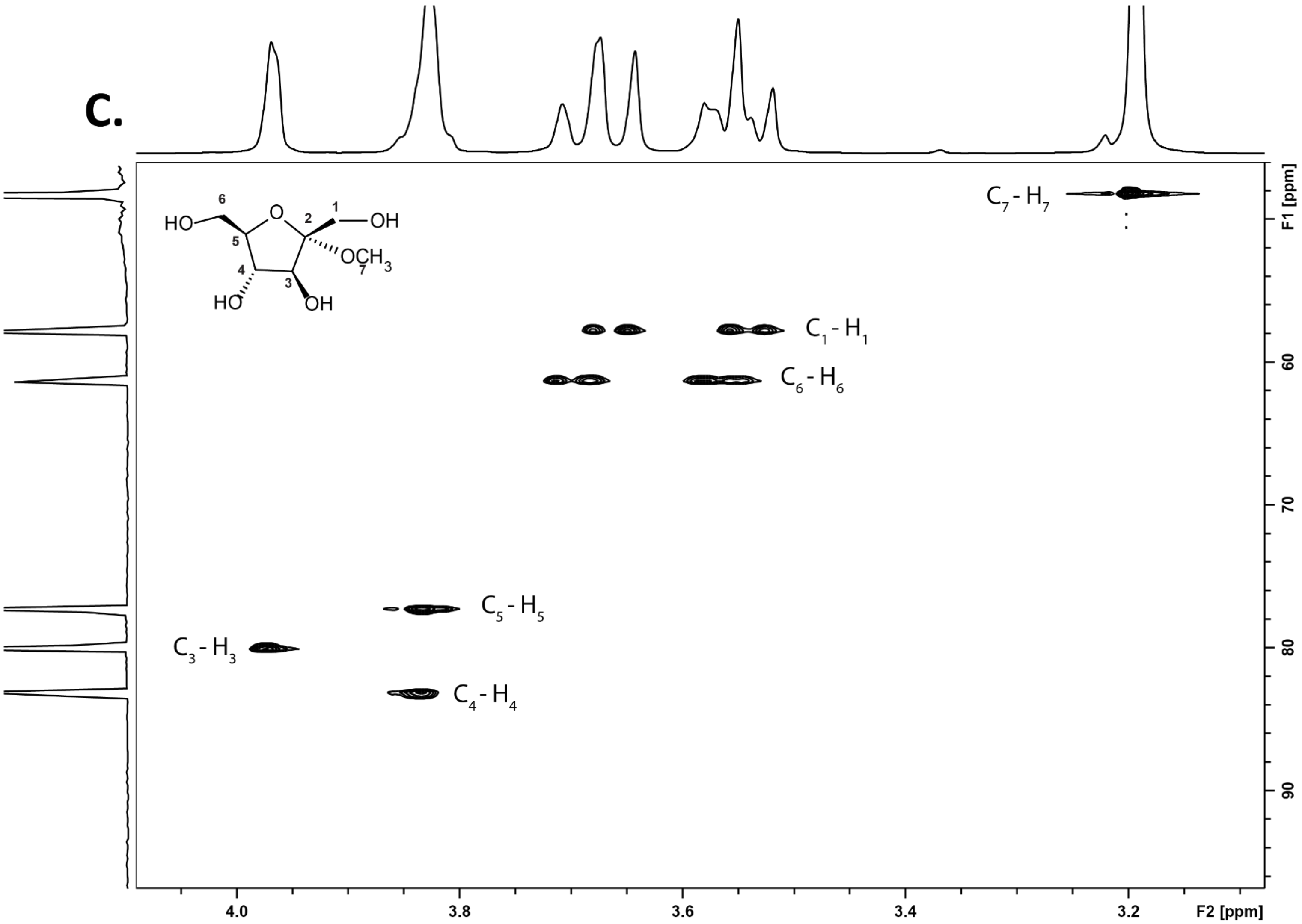

Figure S10. Complete assignment of proton and carbon nuclei from ^{1}H,^{13}C HSQC of fructose derivatives. A. Methyl β-D-fructopyranoside. B. Methyl β-D-fructofuranoside. C. Methyl α -D-fructofuranoside. Each black crosspeak corresponds to a $^{1}J_{C,H}$-coupling interaction from the HSQC experiment.

A.

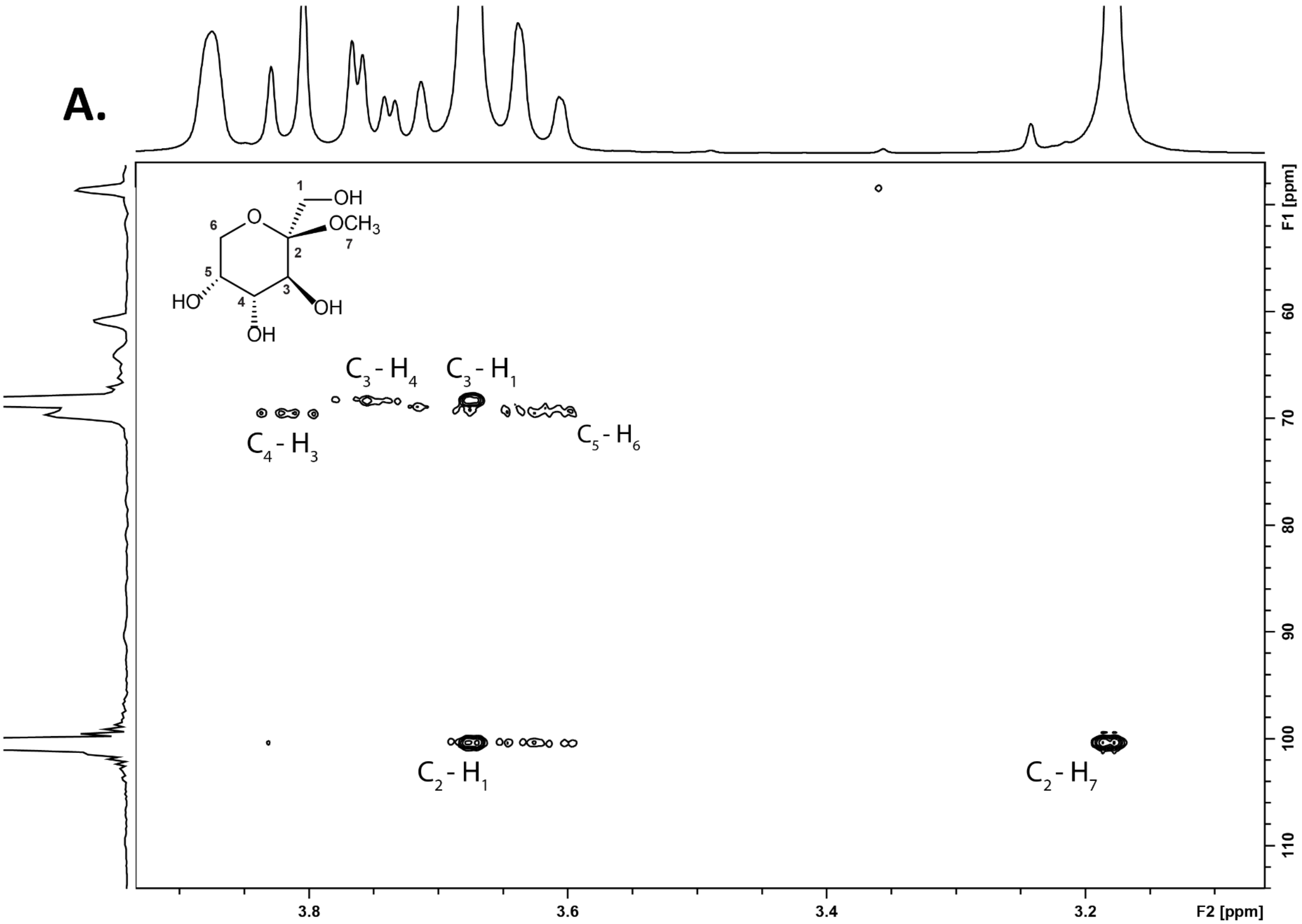

1
OH
O
OCH3
6
7
2
5
3
4
HO
OH
OH
C3 - H4
C3 - H1
C4 - H3
C5 - H6
C2 - H1
C2 - H7
F1 [ppm]
60
70
80
90
100
110
3.8
3.6
3.4
3.2
F2 [ppm]

B.

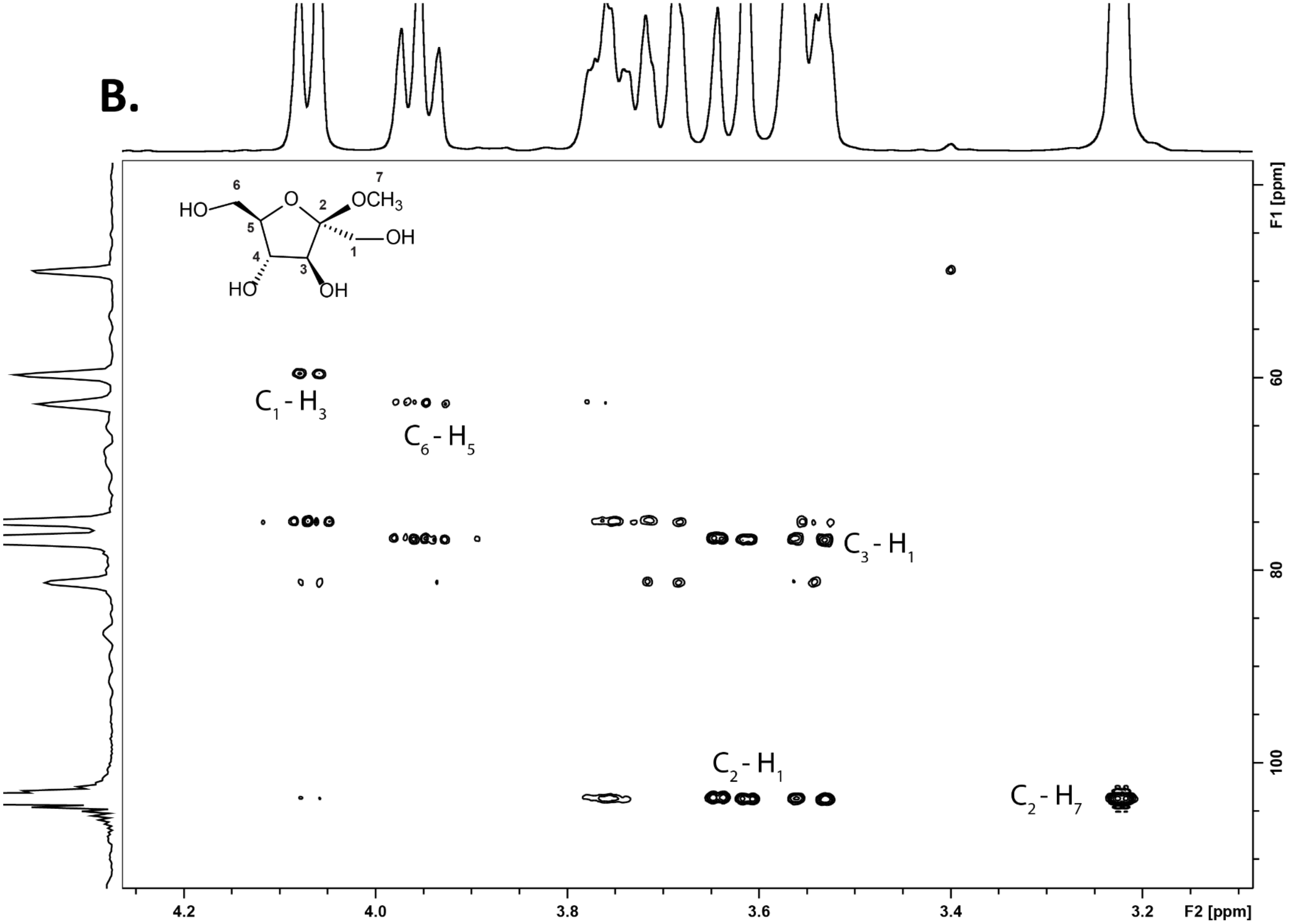
HO
O
OCH3
OH
HO
OH
1
2
3
4
5
6
7
C1 - H3
C6 - H5
C3 - H1
C2 - H1
C2 - H7
F1 [ppm]
60
80
100
4.2
4.0
3.8
3.6
3.4
3.2
F2 [ppm]

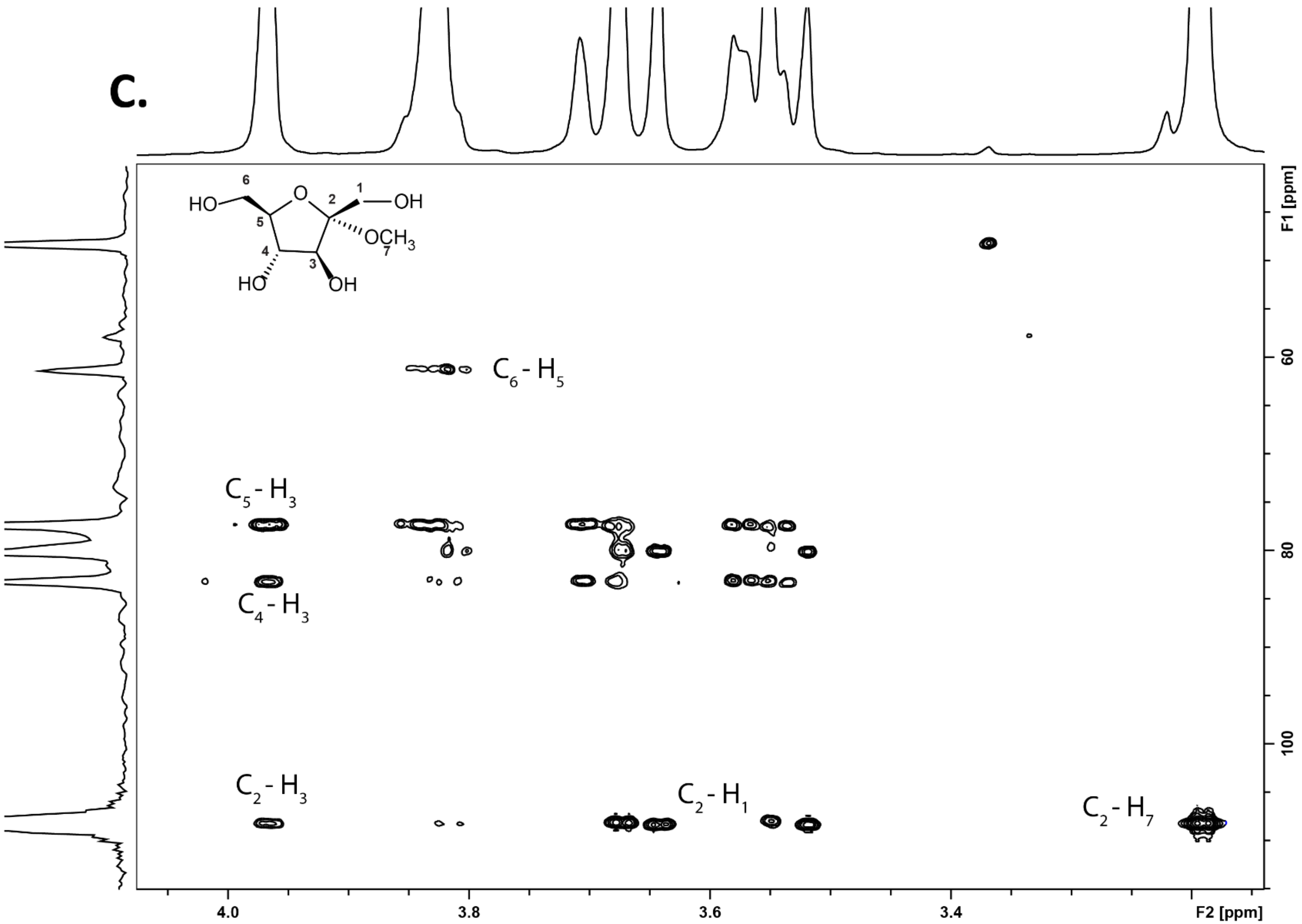

Figure S11. Selective assignment of proton and carbon nuclei from ^{1}H,^{13}C HMBC of fructose derivatives. A. Methyl β-D-fructopyranoside. B. Methyl β-D-fructofuranoside. C. Methyl α-D-fructofuranoside. Each black crosspeak corresponds to a $^{2}J_{C,H}$- or $^{3}J_{C,H}$ -coupling interaction from the HMBC experiment.

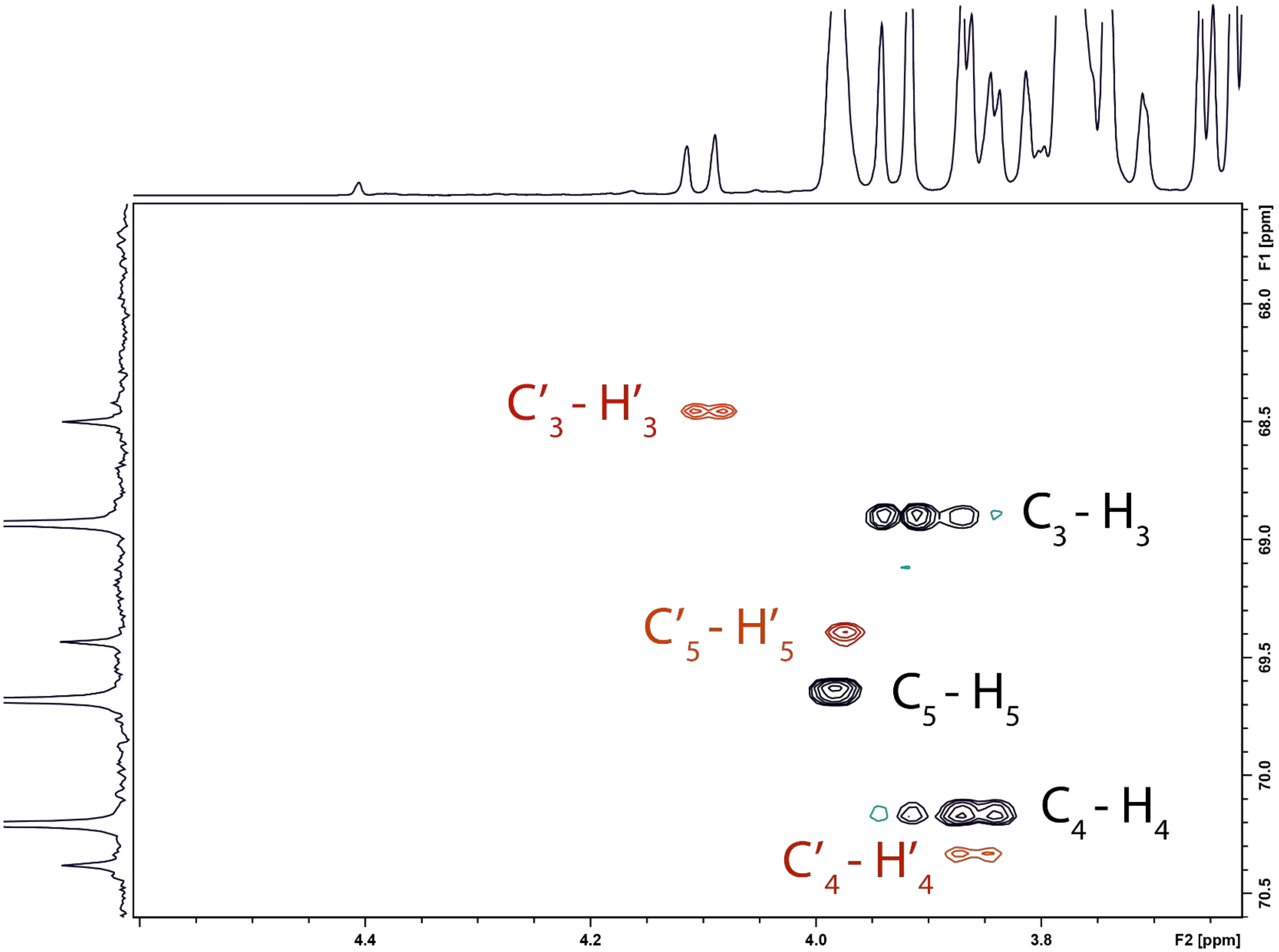

Figure S12. Complete analysis of oxidation reaction components of *Fgr*AAO of methyl β-D-fructopyranose from ^{1}H,^{13}C HSQC zoomed into 3.6-4.6 ppm region. Each black crosspeak corresponds to a $^{1}J_{C,H}$-coupling interaction from the HSQC experiment.

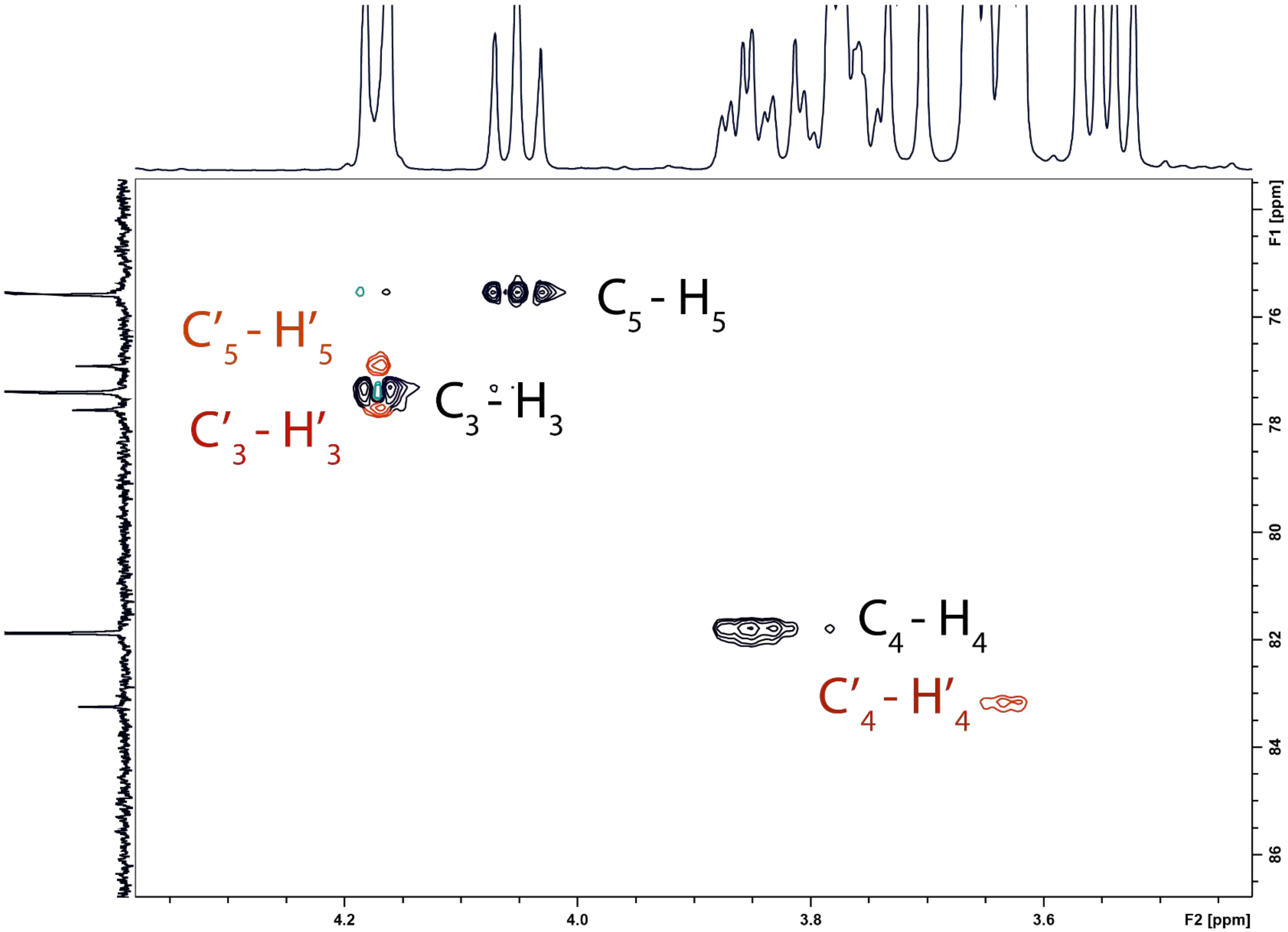

Figure S13. Complete analysis of oxidation reaction components of *Fgr*AAO of methyl β-D-fructofuranose from $^{1}H,^{13}C$ HSQC zoomed into 3.6-4.6 ppm region. Each black crosspeak corresponds to a $^{1}J_{C,H}$-coupling interaction from the HSQC experiment.

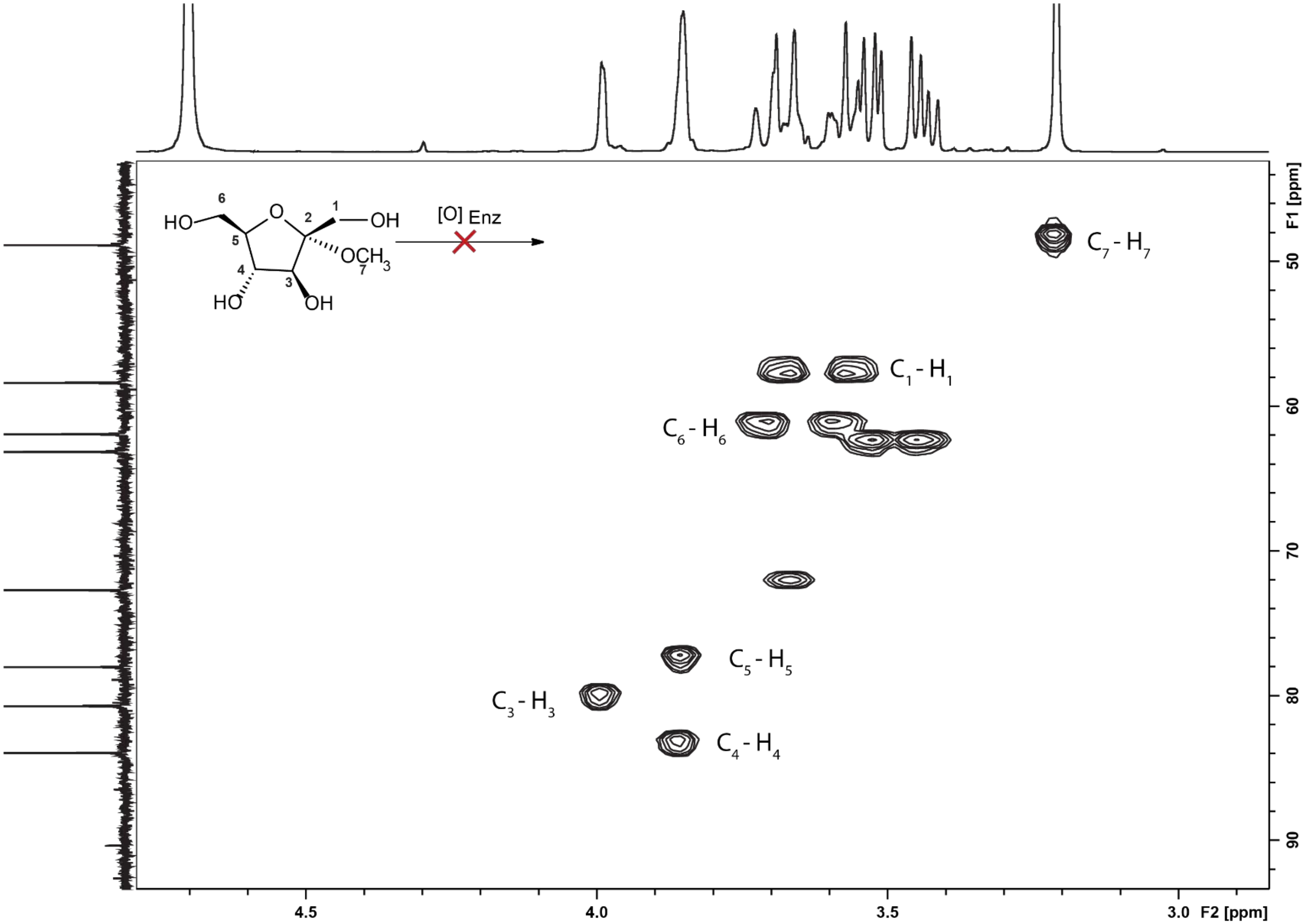

Figure S14. Complete analysis of oxidation reaction components of *Fgr*AAO of methyl α-D-fructofuranoside from ^{1}H,^{13}C HSQC. Each black crosspeak corresponds to a $^{1}J_{C,H}$-coupling interaction from the HSQC experiment. Two carbon peaks at 63.1 ppm and 72.7 ppm have been attributed to an impurity that is present in either the HRP, catalase or buffer solutions.

A.

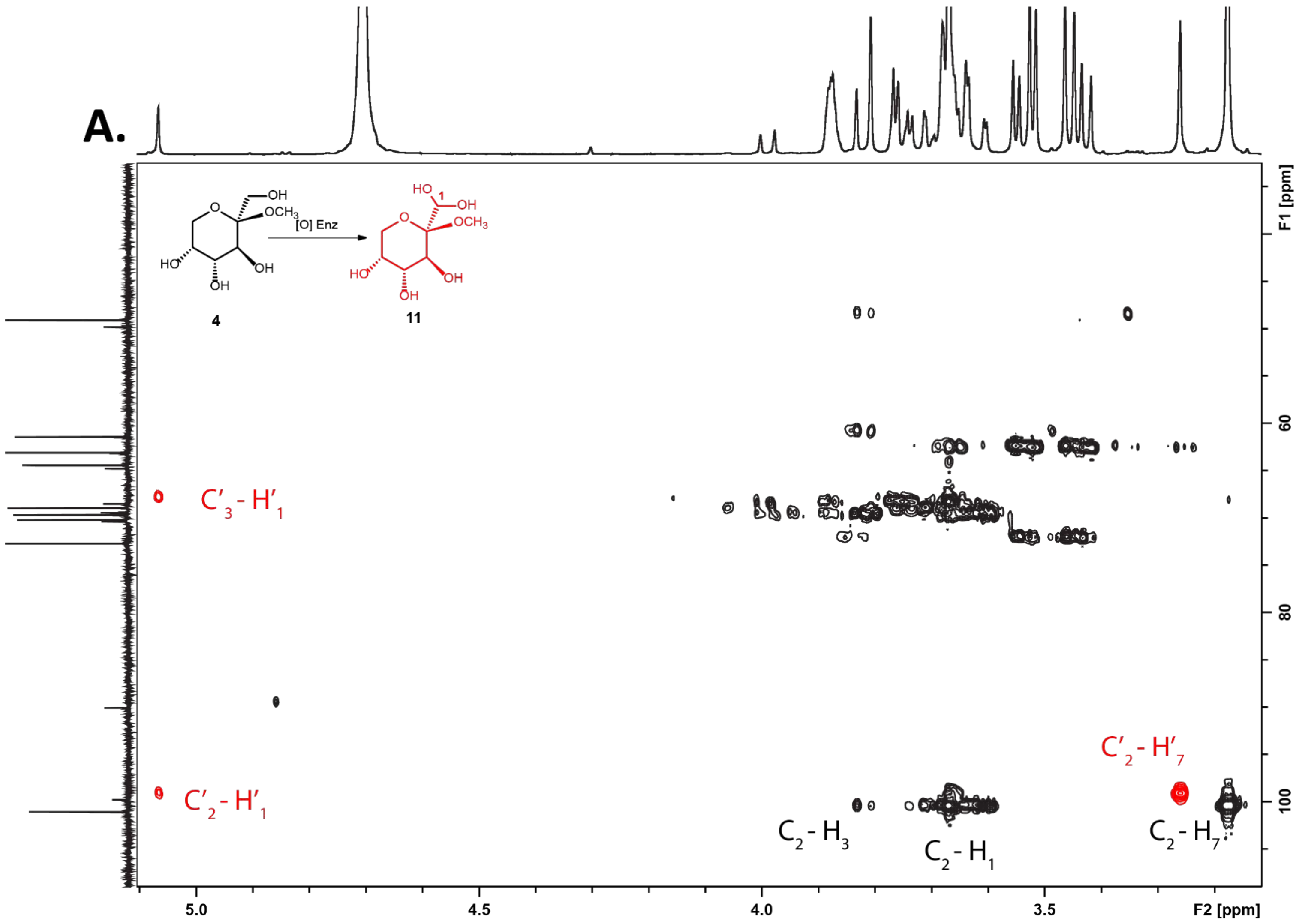

HO
1
OH
OCH3
[O] Enz
O
HO
OH
OH
4
11
C′3 - H′1
C′2 - H′1
C′2 - H′7
C2 - H3
C2 - H1
C2 - H7
5.0
4.5
4.0
3.5
F2 [ppm]
F1 [ppm]
60
80
100

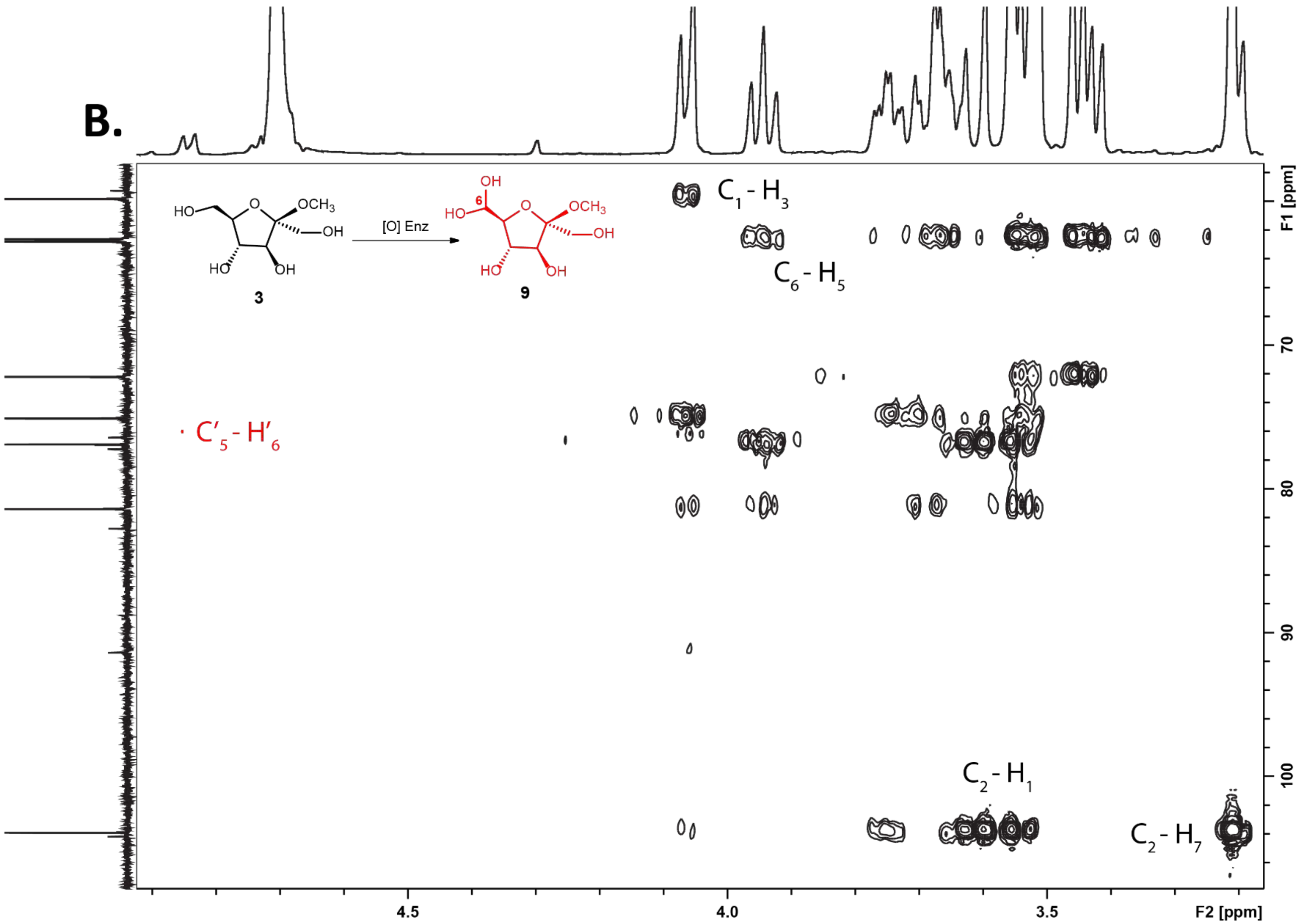

B.
HO
O
OCH3
OH
HO
OH
3
[O] Enz
OH
6
HO
O
OCH3
OH
HO
OH
9
C1 - H3
C6 - H5
C'5 - H'6
C2 - H1
C2 - H7
F1 [ppm]
70
80
90
100
4.5
4.0
3.5
F2 [ppm]

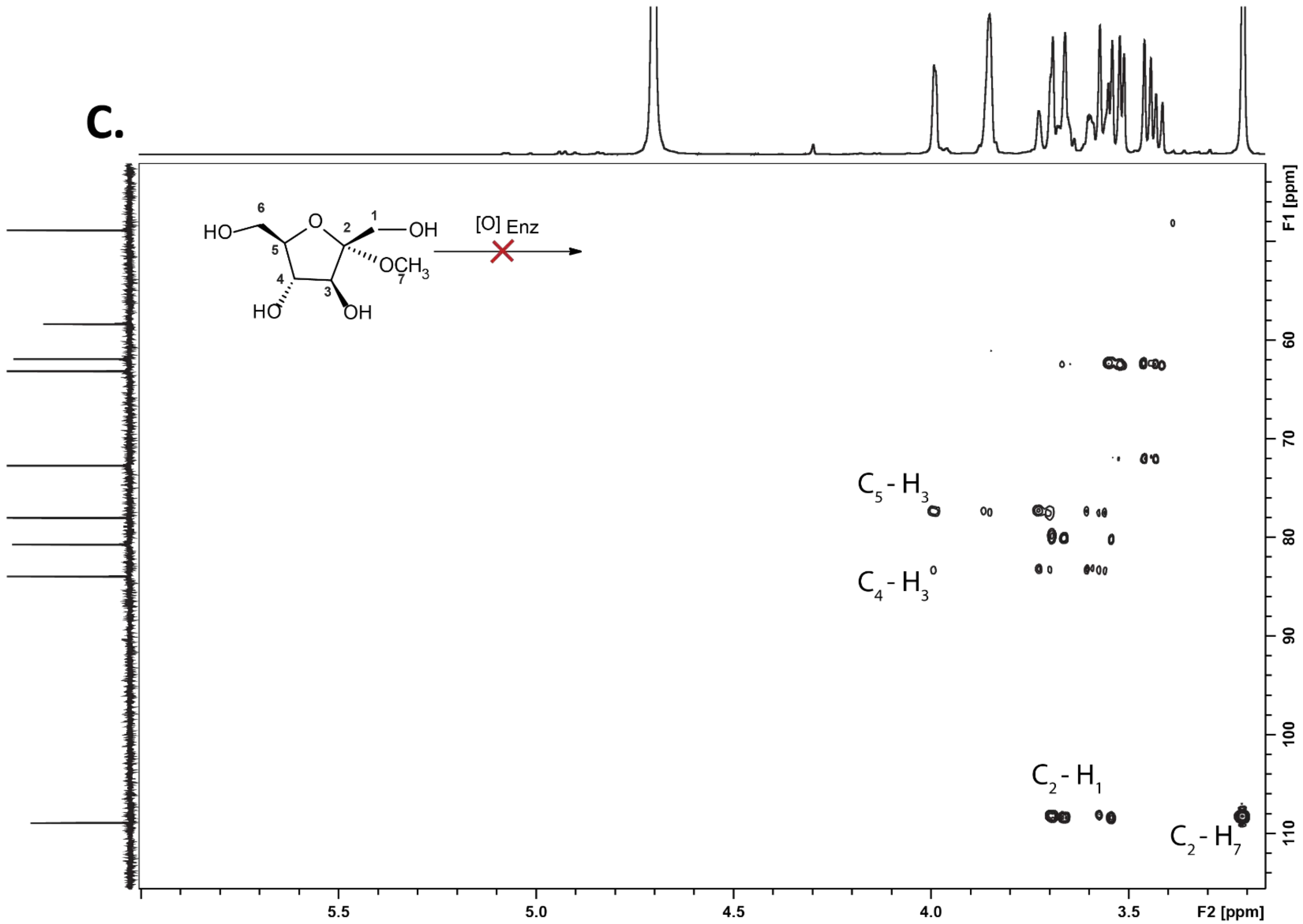

Figure S15. Selective assignment of proton and carbon nuclei from $^{1}H,^{13}C$ HMBC of oxidation reaction components of *Fgr*AAO with fructose derivatives. A. Methyl β-D-fructopyranoside. B. Methyl β-D-fructofuranoside. C. Methyl α-D-fructofuranoside. Each black crosspeak corresponds to a $^{2}J_{C,H}$- or $^{3}J_{C,H}$ -coupling interaction from the HMBC experiment. Two carbon peaks at 63.1 ppm and 72.7 ppm have been attributed to an impurity that is present in either the HRP, catalase or buffer solutions.

A.

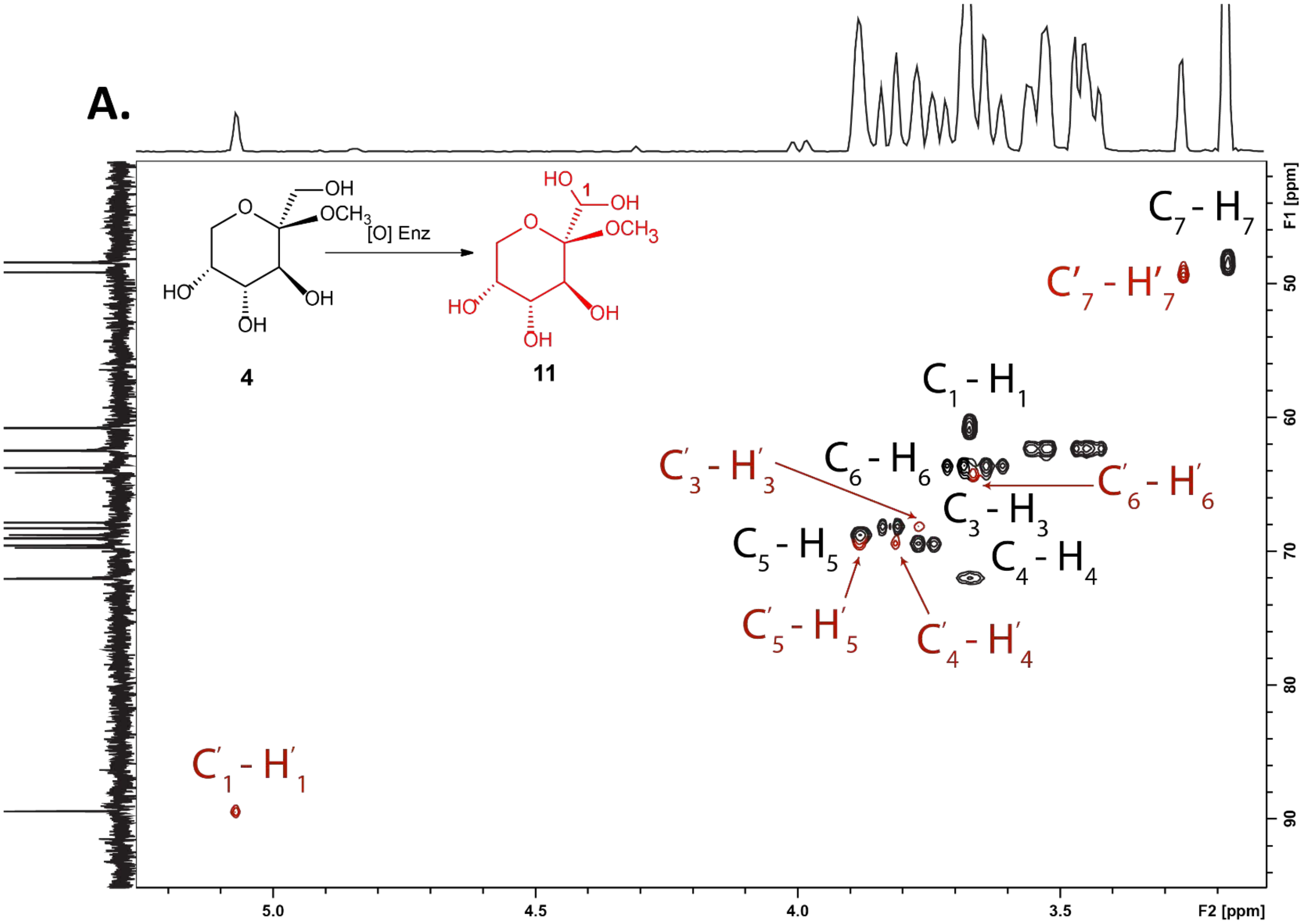

OH
OCH3
[O] Enz
HO
OH
OH
4
HO
1
OH
OCH3
HO
OH
OH
11
C7 - H7
C'7 - H'7
C1 - H1
C'3 - H'3
C6 - H6
C'6 - H'6
C3 - H3
C5 - H5
C4 - H4
C'5 - H'5
C'4 - H'4
C'1 - H'1
F1 [ppm]
50
60
70
80
90
5.0
4.5
4.0
3.5
F2 [ppm]

B.

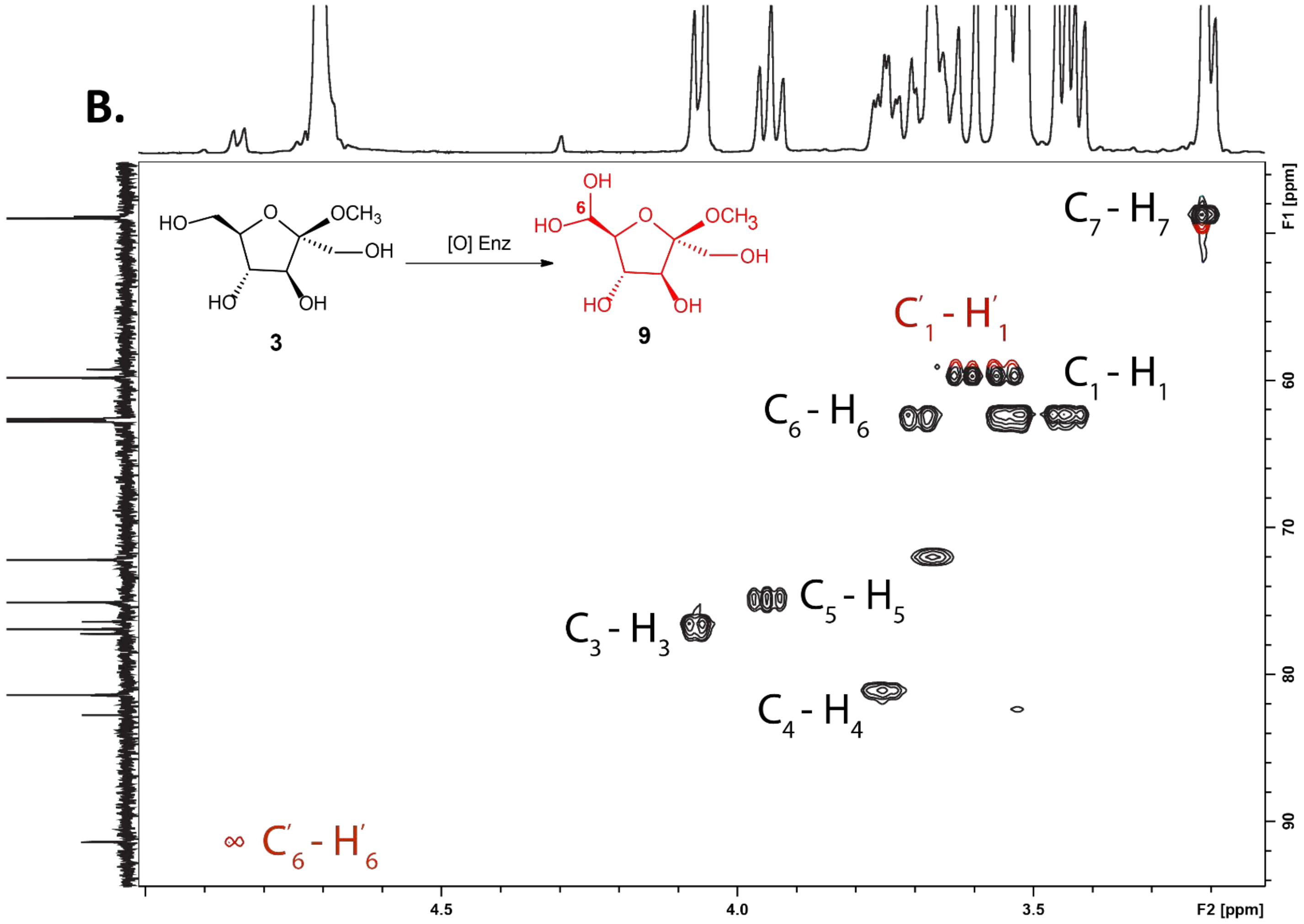

HO
O
OCH3
OH
HO
OH
3
[O] Enz
OH
6
HO
O
OCH3
OH
HO
OH
9
C7 - H7
C'1 - H'1
C1 - H1
C6 - H6
C5 - H5
C3 - H3
C4 - H4
C'6 - H'6
F1 [ppm]
60
70
80
90
4.5
4.0
3.5
F2 [ppm]

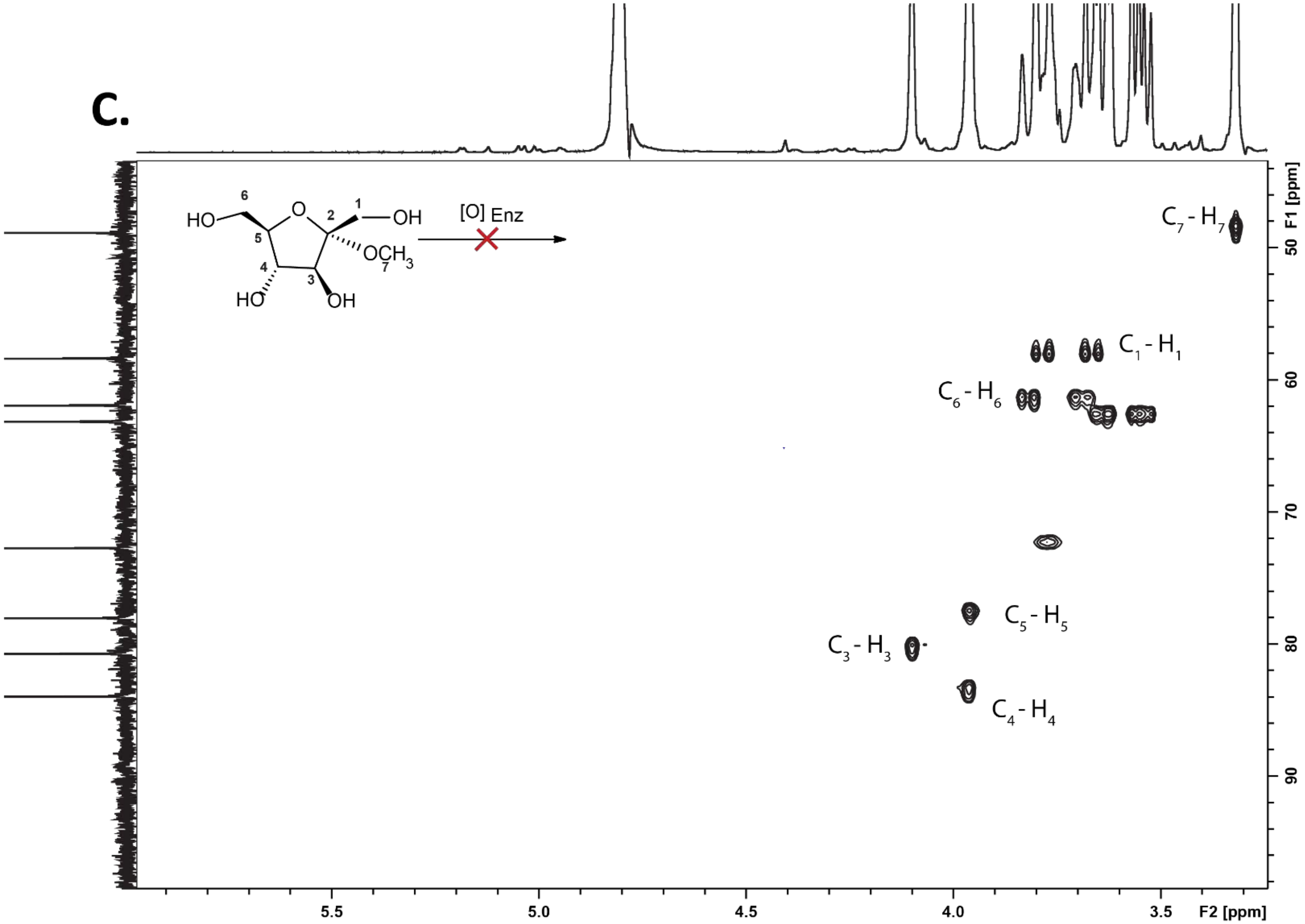

Figure S16. Complete analysis of oxidation reaction components of *Fox*AAO of fructose derivatives from ^{1}H,^{13}C HSQC. A. Methyl β-D-fructopyranoside. B. Methyl β-D-fructofuranoside. C. Methyl α -D-fructofuranoside. Each black crosspeak corresponds to a $^{1}J_{C,H}$-coupling interaction from the HSQC experiment. Two carbon peaks at 63.1 ppm and 72.7 ppm have been attributed to an impurity that is present in either the HRP, catalase or buffer solutions.

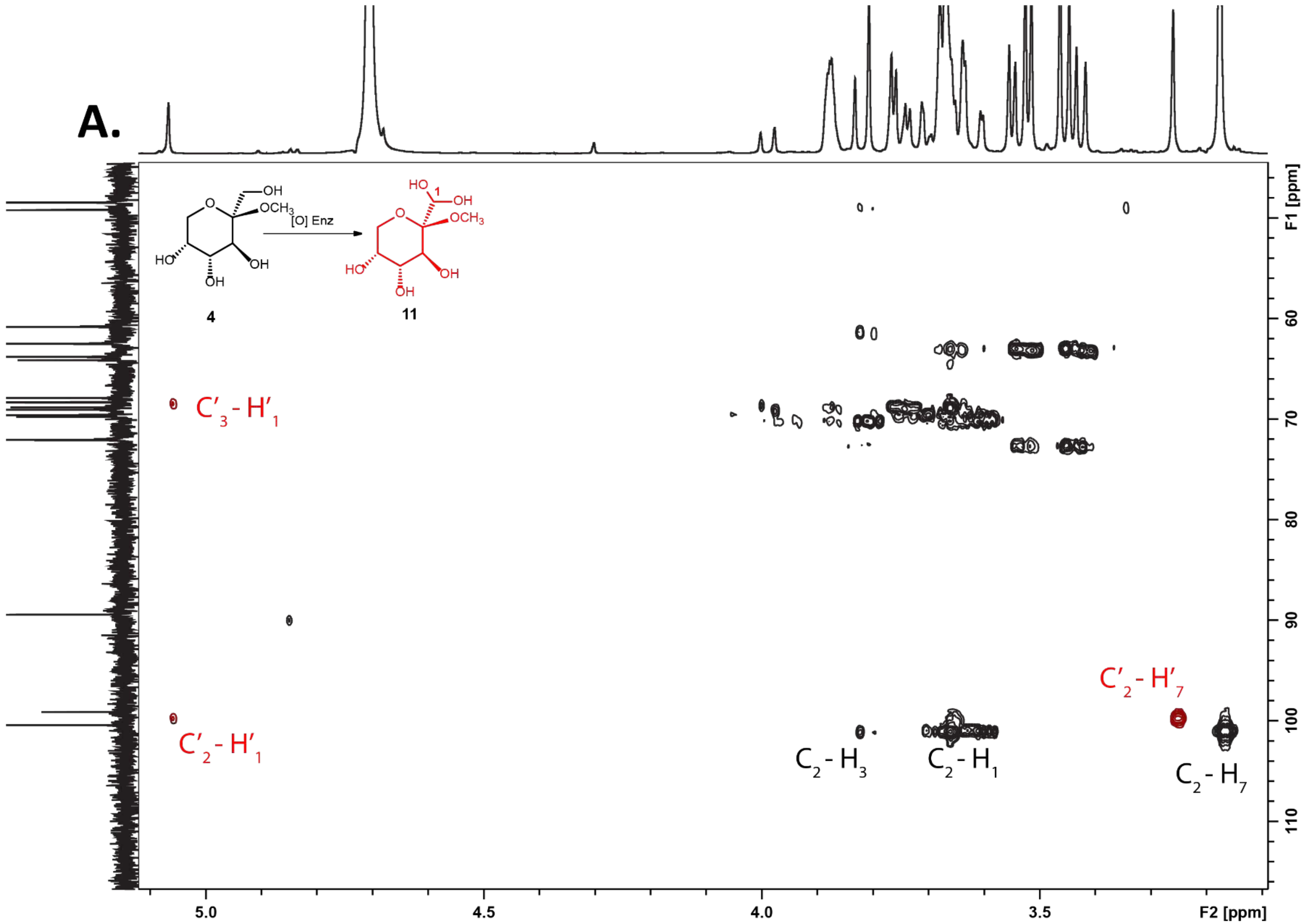
A.
4
11
[O] Enz
C'3 - H'1
C'2 - H'1
C'2 - H'7
C2 - H3
C2 - H1
C2 - H7
F1 [ppm]
F2 [ppm]

B.

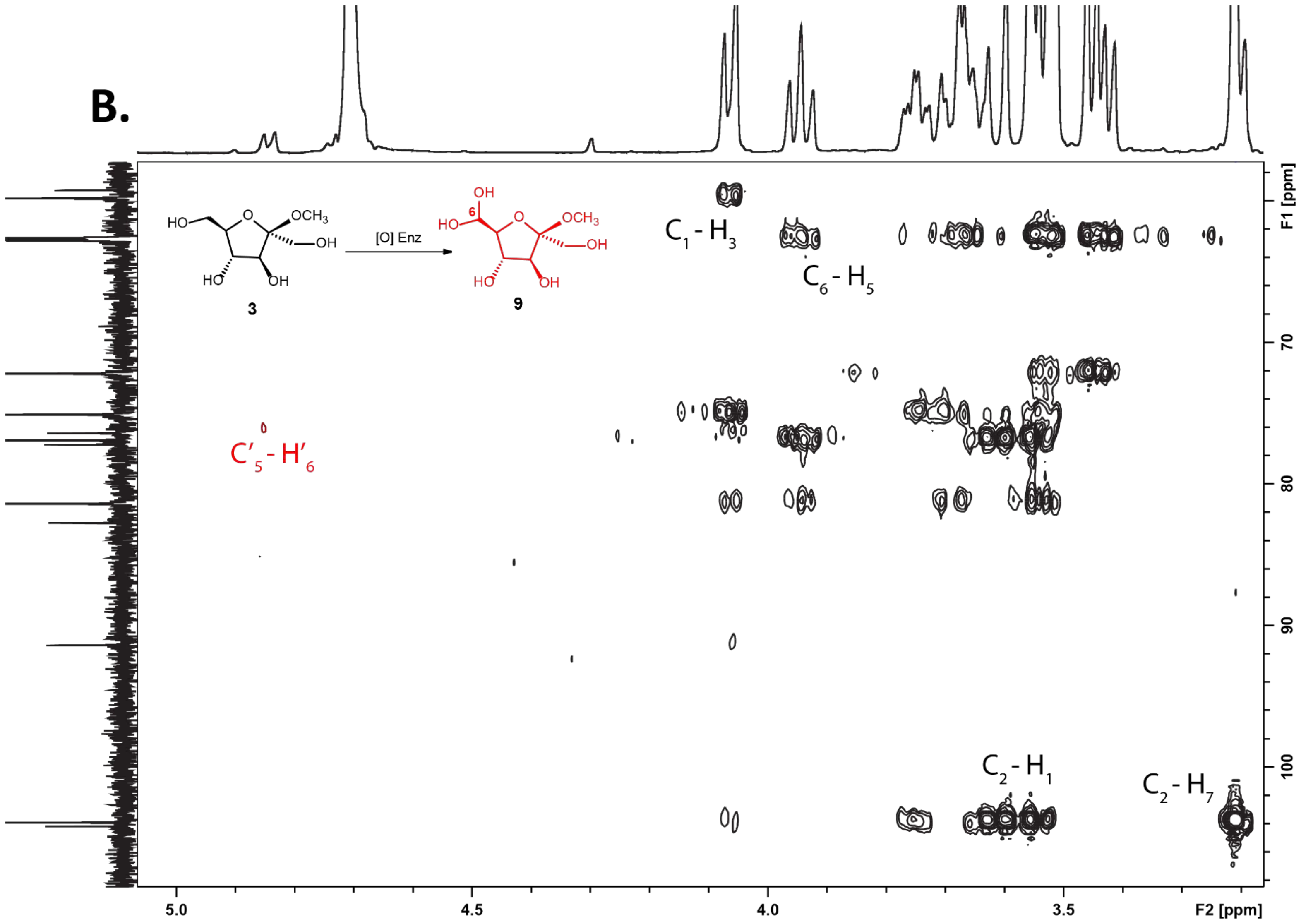

HO
O
OCH3
OH
HO
OH
3
[O] Enz
OH
6
HO
O
OCH3
OH
HO
OH
9
C1 - H3
C6 - H5
C'5 - H'6
C2 - H1
C2 - H7
F1 [ppm]
70
80
90
100
5.0
4.5
4.0
3.5
F2 [ppm]

C.

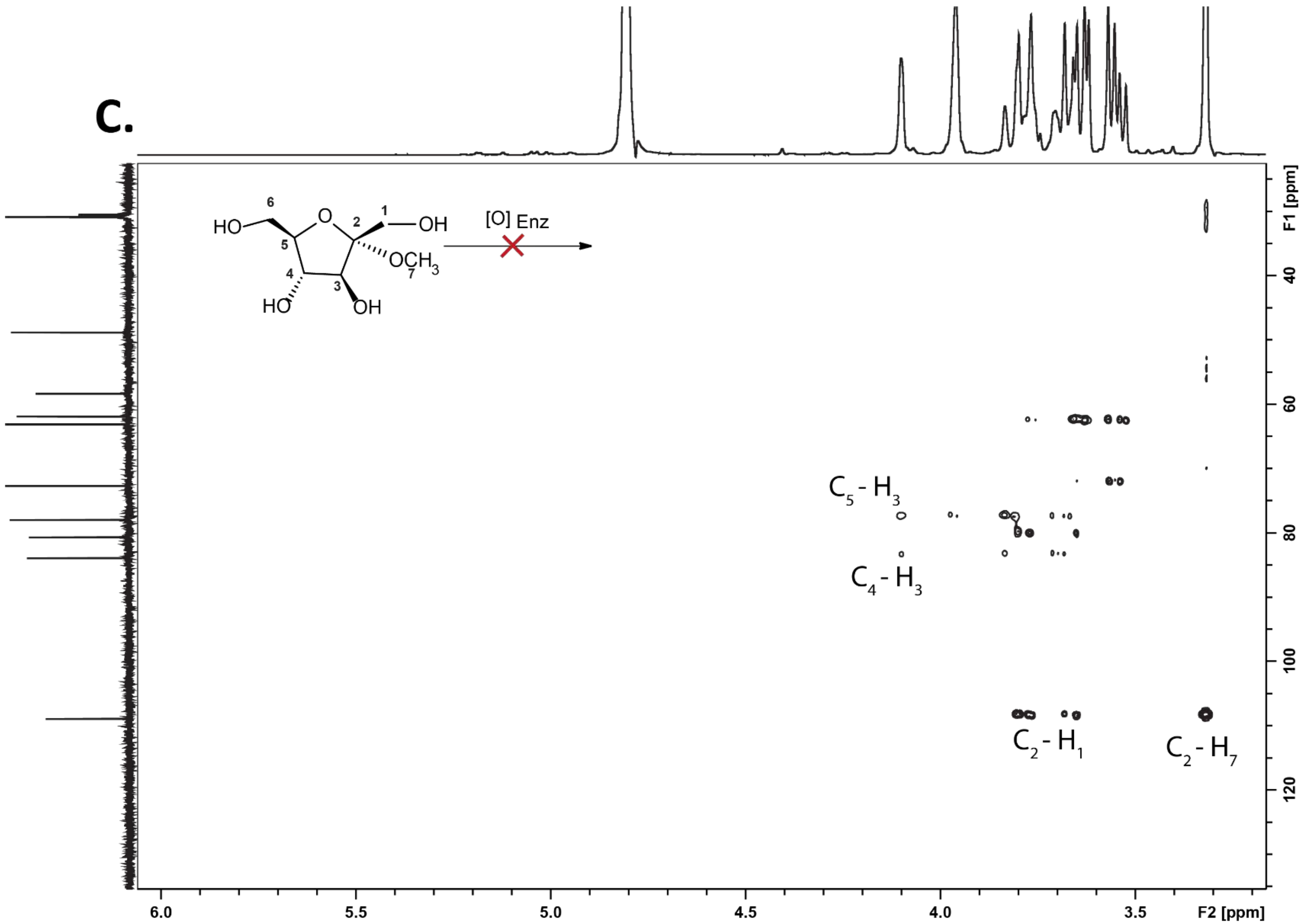

Figure S17. Selective assignment of proton and carbon nuclei from ^{1}H,^{13}C HMBC of oxidation reaction components of *Fox*AAO with fructose derivatives. A. Methyl β-D-fructopyranoside. B. Methyl β-D-fructofuranoside. C. Methyl α-D-fructofuranoside. Each black crosspeak corresponds to a $^{2}J_{C,H}$- or $^{3}J_{C,H}$ -coupling interaction from the HMBC experiment. Two carbon peaks at 63.1 ppm and 72.7 ppm have been attributed to an impurity that is present in either the HRP, catalase or buffer solutions.

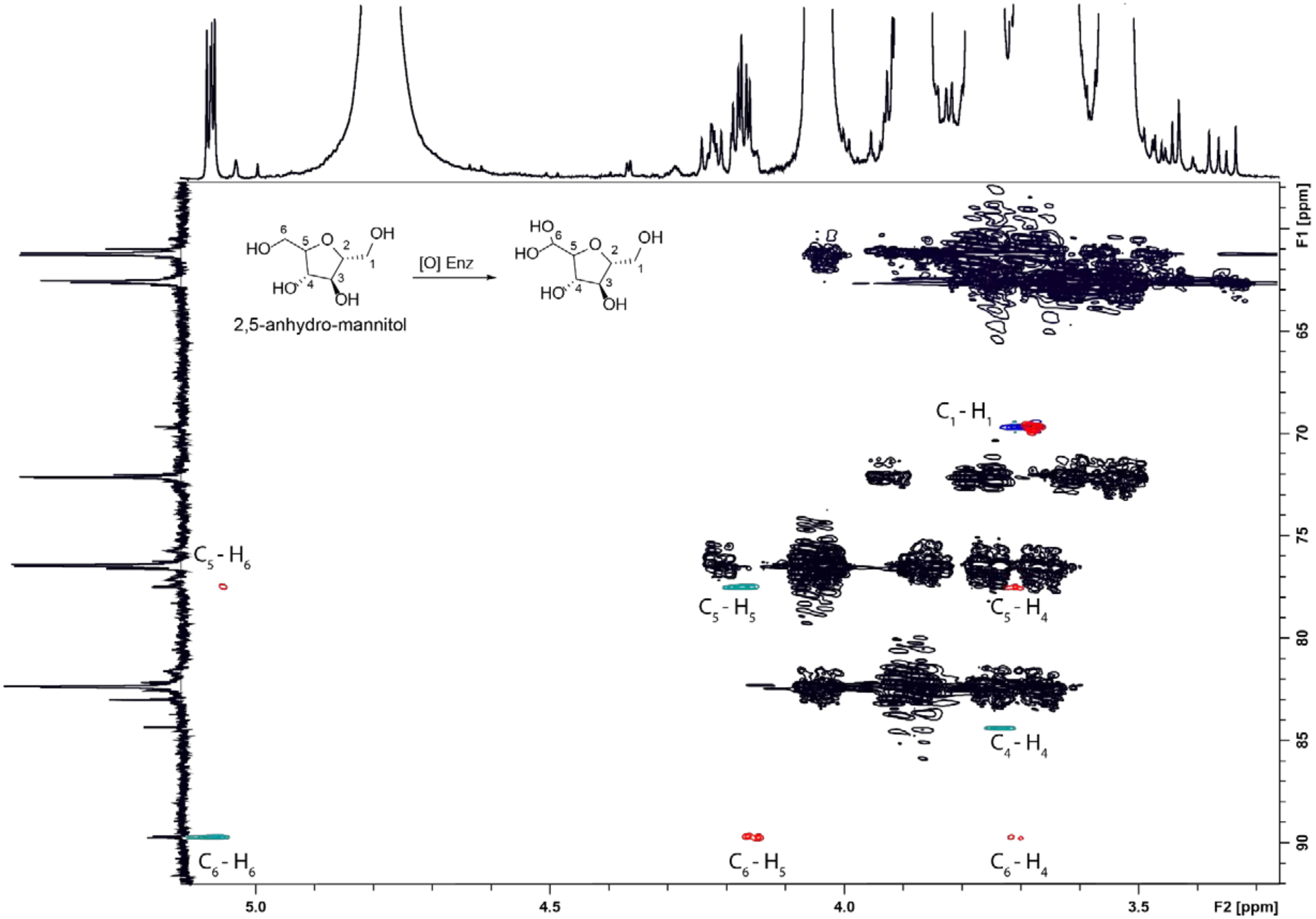

Figure S18. Selective assignment of proton and carbon nuclei from ^{1}H,^{13}C HSQC and HMBC of oxidation product *Fgr*AAO with 2,5-anhydro-mannitol. Each teal and blue crosspeak corresponds to a $^{1}J_{C,H}$-coupling interaction from the HSQC experiment while each red crosspeak corresponds to a $^{2}J_{C,H}$- or $^{3}J_{C,H}$ -coupling interaction from the HMBC experiment. Two carbon peaks at 63.1 ppm and 72.7 ppm have been attributed to an impurity that is present in either the HRP, catalase or buffer solutions.

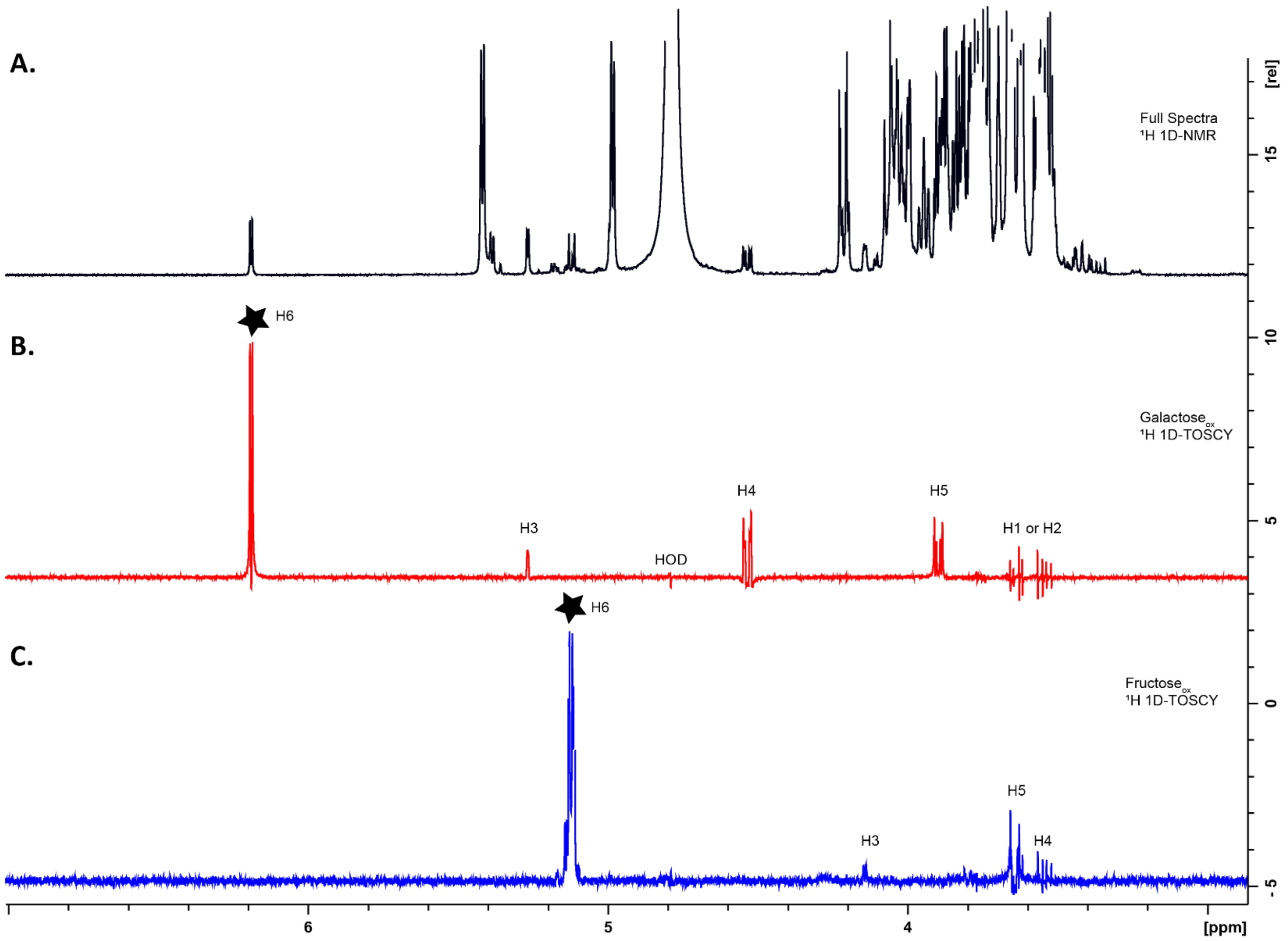

Figure S19. Selective ^{1}H 1D-TOSCY experiment of oxidized raffinose by *Fgr*AAO. A. New proton peaks in the proton region between 5.0-6.5 ppm were targeted based on the full 1D-NMR spectrum. Spectra corresponding to individual spin systems are displayed in various colours with B. the galactose ring of oxidized raffinose in red. C. the fructose ring in oxidized raffinose in blue. Irradiations from ^{1}H 1D-TOSCY experiments are displayed with black stars above corresponding peaks.

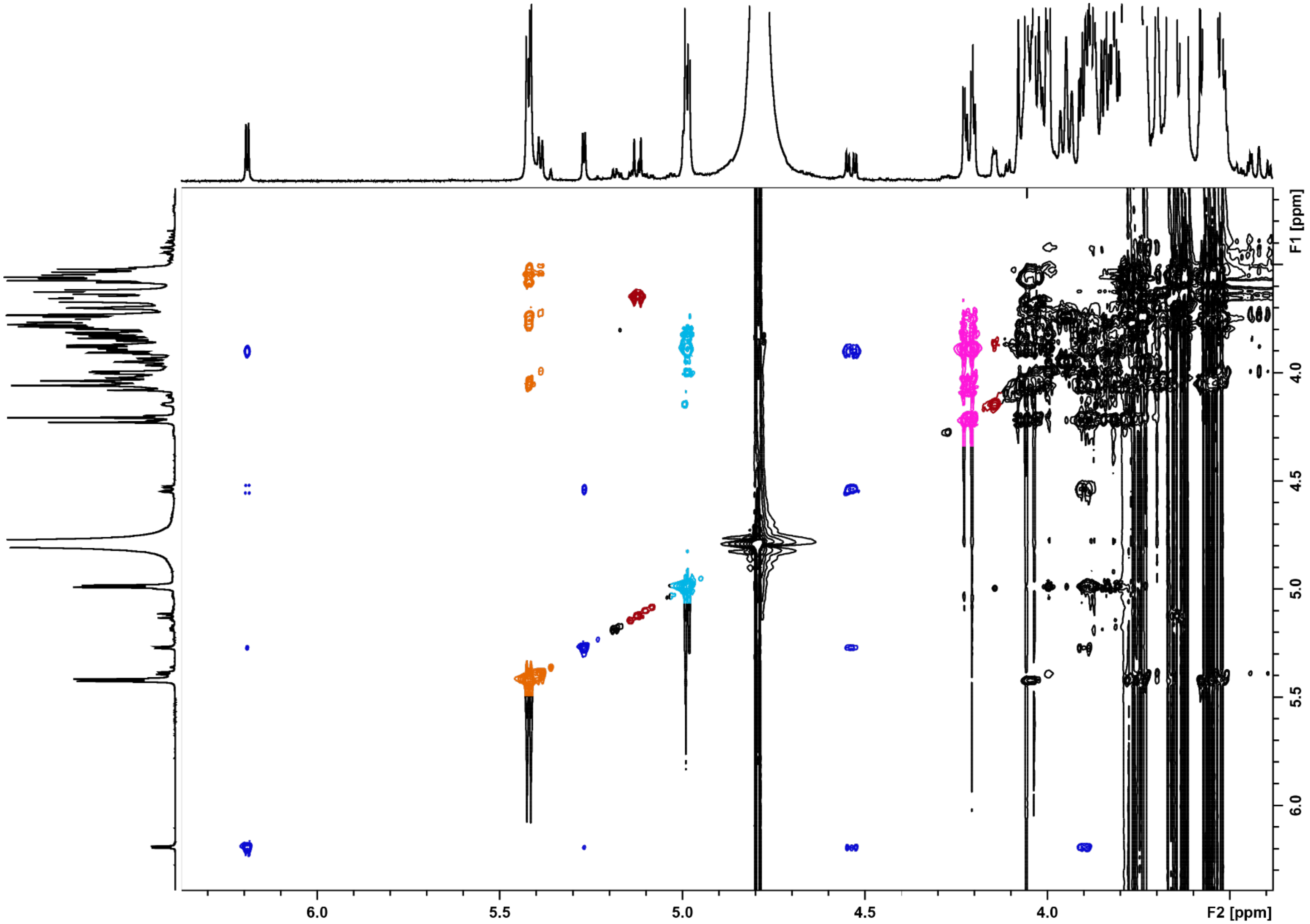

Figure S20. [1]H 2D-TOSCY experiment oxidized raffinose by *Fgr*AAO. Each peak indicates protons that are coupled through magnetization transfer. Selective assignment of crosspeaks originating from the same spin system are shown in matching colours specific to each monosaccharide with oxidized/unoxidized galactose shown in dark blue and light blue respectively; oxidized/unoxidized fructose shown in dark red and magenta respectively; glucose in orange.

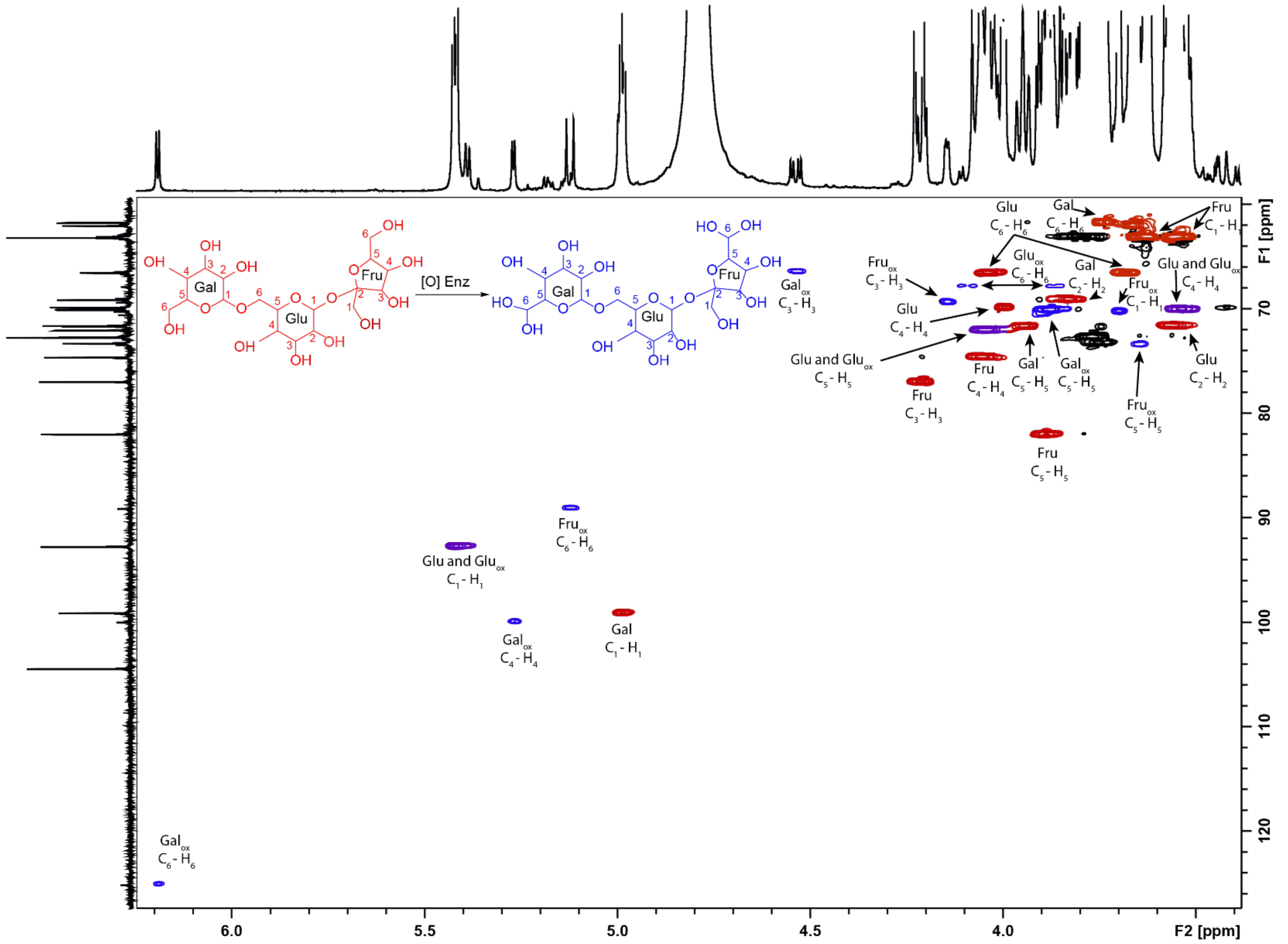

Figure S21. Complete assignment of proton and carbon nuclei from oxidized raffinose by *Fgr*AAO using ^{1}H,^{13}C HSQC experiments. Each crosspeak corresponds to a $^1J_{C,H}$-coupling interaction from the HSQC experiment. Red crosspeaks correspond to unoxidized substrate, blue crosspeaks correspond to oxidized product and purple crosspeaks correspond to signals overlapping from substrate and product. Black crosspeaks are from impurities associated with the buffer, HRP and catalase mixture. The notation stated the sugar ring and its corresponding atoms(s) below it. Abbreviations used: Gal, galactose; Glu, glucose, Fru, fructose; ox, Oxidized.

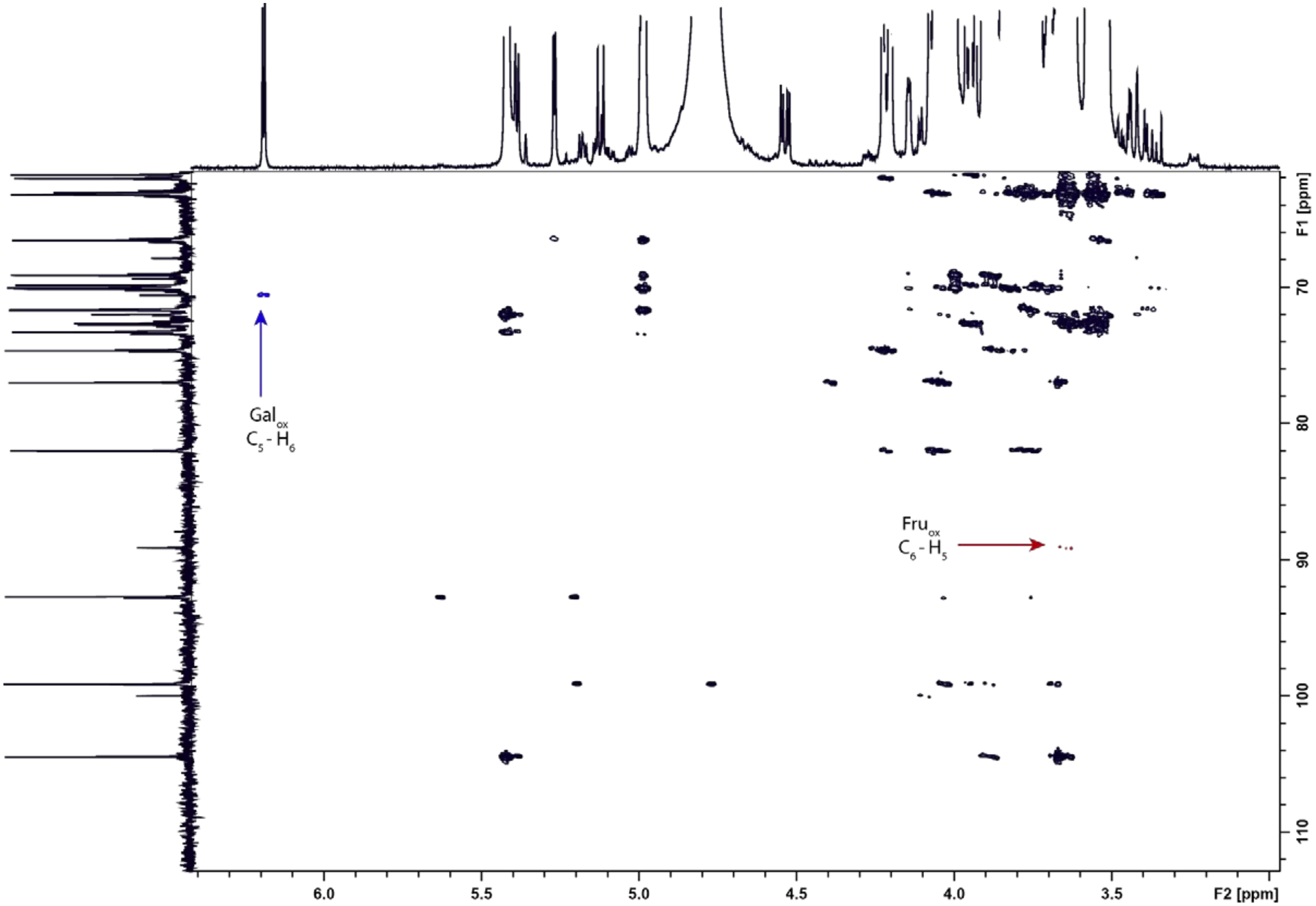

Figure S22. Selective assignment of proton and carbon nuclei from $^{1}H,^{13}C$ HMBC of oxidation reaction components of *Fgr*AAO with raffinose. Each crosspeak corresponds to a $^{2}J_{C,H}$- or $^{3}J_{C,H}$ -coupling interaction from the HMBC experiment. Crosspeaks for C5-H6 on the galactose ring in the product and C6-H5 on the fructose ring in the oxidized product are highlighted in blue and red, respectively. Two carbon peaks at 63.1 ppm and 72.7 ppm have been attributed to an impurity that is present in either the HRP, catalase or buffer solutions.

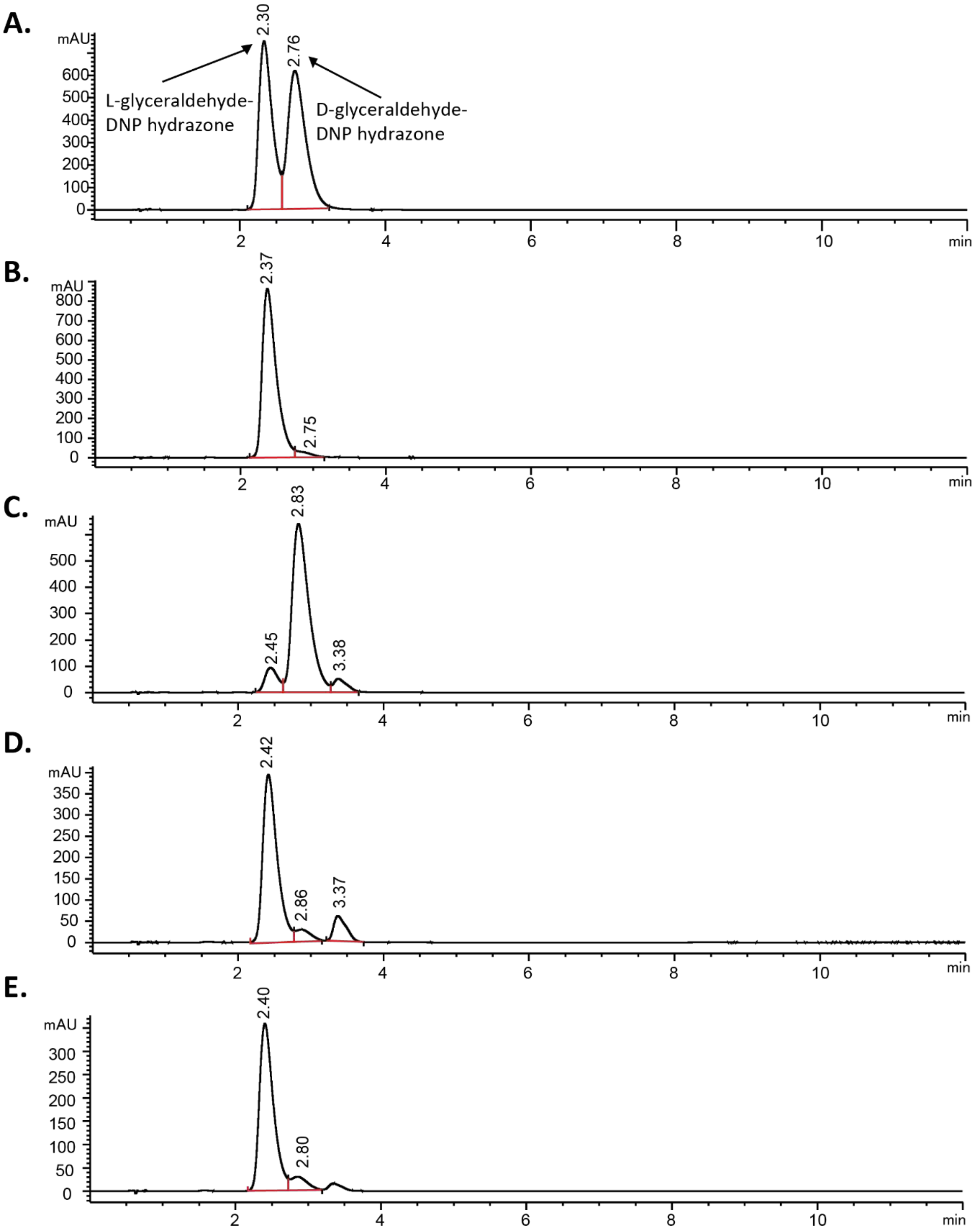

Figure S23. Stereochemistry determination of glycerol oxidation by *Fgr*AAO and *Fox*AAO. A. Chromatogram of *L*/*D*-glyceraldehyde-hydrazone. B. Glyceraldehyde-hydrazone composition after *Fgr*GalOx oxidation of glycerol. C. Glyceraldehyde-hydrazone composition after *Cgr*AlcOx oxidation of glycerol. D. Glyceraldehyde-hydrazone composition after *Fgr*AAO oxidation of glycerol. E. Glyceraldehyde-hydrazone composition after *Fox*AAO oxidation of glycerol. Peaks are labelled with retention times.

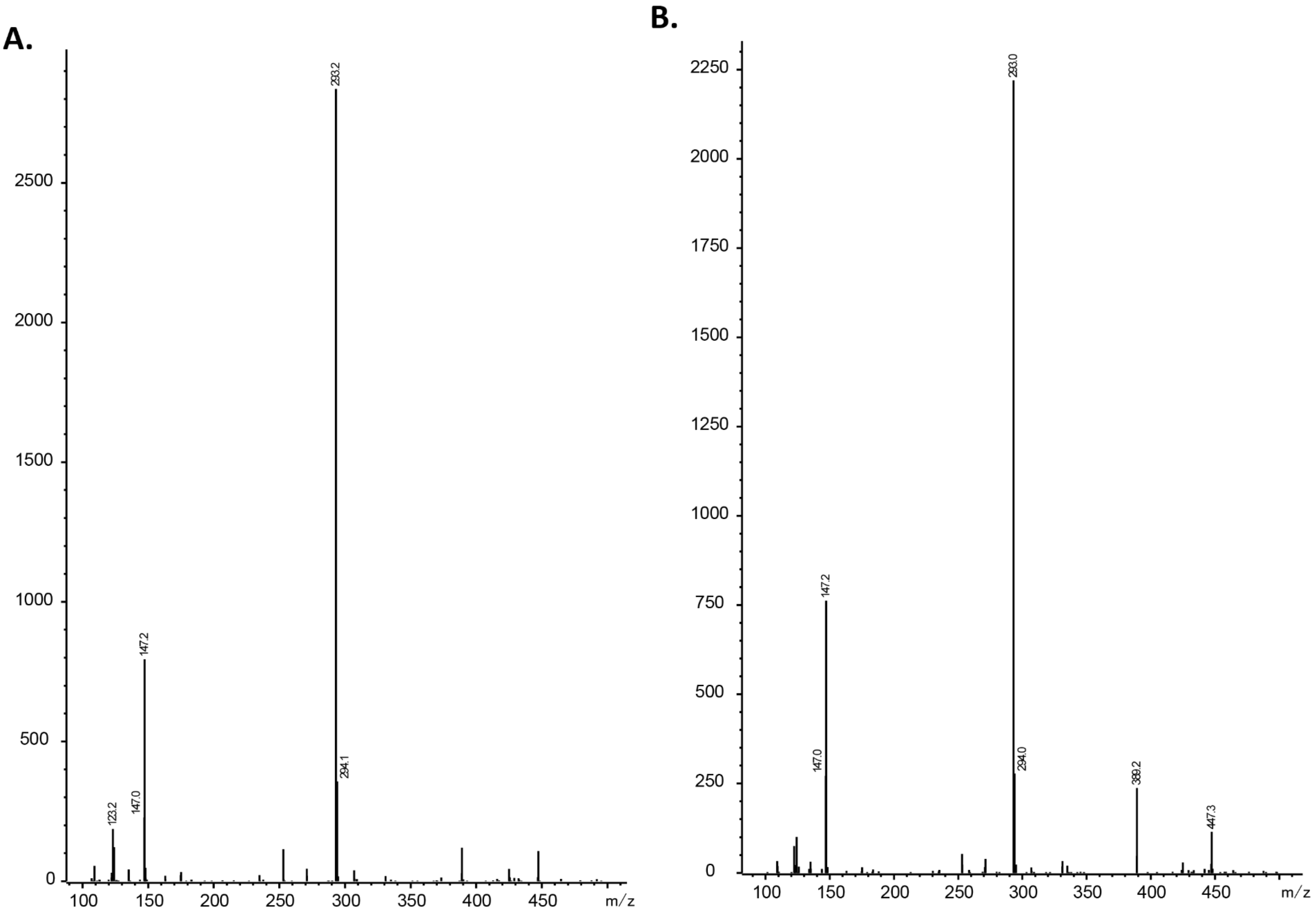

Figure S24. Representative mass spectrum of glycerol oxidation by *Fgr*GalOx and *Cgr*AlcOx. A. L-glyceraldehyde-hydrazone from *Fgr*GalOx oxidation (peak at 2.37, Fig. S23). B. D-glyceraldehyde-hydrazone from *Cgr*AlcOx oxidation (peak at 2.83, Fig. S23).

Supporting References

1. Deacon SE, Mahmoud K, Spooner RK, Firbank SJ, Knowles PF, Phillips SEV, McPherson MJ. Enhanced Fructose Oxidase Activity in a Galactose Oxidase Variant. ChemBioChem. 2004;5(7):972-9.
2. Sun L, Petrounia IP, Yagasaki M, Bandara G, Arnold FH. Expression and stabilization of galactose oxidase in Escherichia coli by directed evolution. Protein Eng, Des Sel. 2001;14(9):699-704.
3. Escalettes F, Turner NJ. Directed Evolution of Galactose Oxidase: Generation of Enantioselective Secondary Alcohol Oxidases. ChemBioChem. 2008;9(6):857-60.
4. Lippow SM, Moon TS, Basu S, Yoon S-H, Li X, Chapman BA, Robison K, Lipovšek D, Prather KLJ. Engineering Enzyme Specificity Using Computational Design of a Defined-Sequence Library. Chem Biol. 2010;17(12):1306-15.
5. Rannes JB, Ioannou A, Willies SC, Grogan G, Behrens C, Flitsch SL, Turner NJ. Glycoprotein Labeling Using Engineered Variants of Galactose Oxidase Obtained by Directed Evolution. J Am Chem Soc. 2011;133(22):8436-9.
6. Deacon SE, McPherson MJ. Enhanced Expression and Purification of Fungal Galactose Oxidase in Escherichia coli and Use for Analysis of a Saturation Mutagenesis Library. ChemBioChem. 2011;12(4):593-601.
7. Wilkinson D, Akumanyi N, Hurtado-Guerrero R, Dawkes H, Knowles PF, Phillips SEV, McPherson MJ. Structural and kinetic studies of a series of mutants of galactose oxidase identified by directed evolution. Protein Eng, Des Sel. 2004;17(2):141-8.
8. Mathieu Y, Offen WA, Forget SM, Ciano L, Viborg AH, Blagova E, Henrissat B, Walton PH, Davies GJ, Brumer H. Discovery of a Fungal Copper Radical Oxidase with High Catalytic Efficiency toward 5-Hydroxymethylfurfural and Benzyl Alcohols for Bioprocessing. ACS Catal. 2020;10(5):3042-58.
9. Whittaker MM, Whittaker JW. Catalytic Reaction Profile for Alcohol Oxidation by Galactose Oxidase. Biochemistry. 2001;40(24):7140-8.
10. Yin D, Urresti S, Lafond M, Johnston EM, Derikvand F, Ciano L, Berrin J-G, Henrissat B, Walton PH, Davies GJ, Brumer H. Structure–function characterization reveals new catalytic diversity in the galactose oxidase and glyoxal oxidase family. Nat Commun. 2015;6(1):10197.
11. Dijkman WP, Fraaije MW. Discovery and Characterization of a 5-Hydroxymethylfurfural Oxidase from Methylovorus sp. Strain MP688. Appl Environ Microbiol. 2014;80(3):1082-90.
12. Carro J, Ferreira P, Rodríguez L, Prieto A, Serrano A, Balcells B, Ardá A, Jiménez-Barbero J, Gutiérrez A, Ullrich R, Hofrichter M, Martínez AT. 5-hydroxymethylfurfural conversion by fungal aryl-alcohol oxidase and unspecific peroxygenase. FEBS J. 2015;282(16):3218-29.
13. Kadowaki MAS, Godoy MOd, Kumagai PS, Costa-Filho AJd, Mort A, Prade RA, Polikarpov I. Characterization of a New Glyoxal Oxidase from the Thermophilic Fungus Myceliophthora thermophila M77: Hydrogen Peroxide Production Retained in 5-Hydroxymethylfurfural Oxidation. Catalysts. 2018;8(10):476.
14. Daou M, Yassine B, Wikee S, Record E, Duprat F, Bertrand E, Faulds CB. Pycnoporus cinnabarinus glyoxal oxidases display differential catalytic efficiencies on 5-hydroxymethylfurfural and its oxidized derivatives. Fungal Biol Biotechnol. 2019;6(1):4.
